# Continuous and discrete phasor analysis of binned or time-gated periodic decays

**DOI:** 10.1063/5.0027834

**Published:** 2021-03-23

**Authors:** Xavier Michalet

**Affiliations:** Department of Chemistry and Biochemistry, 607 Charles E. Young Drive E., Los Angeles, California 90095, USA

## Abstract

The time-resolved analysis of periodically excited luminescence decays by the phasor method in the presence of time-gating or binning is revisited. Analytical expressions for discrete configurations of square gates are derived, and the locus of the phasors of such modified periodic single-exponential decays is compared to the canonical universal semicircle. The effects of instrument response function offset, decay truncation, and gate shape are also discussed. Finally, modified expressions for the phase and modulus lifetimes are provided for some simple cases. A discussion of a modified phasor calibration approach is presented, and an illustration of the new concepts with examples from the literature concludes this work.

## I. INTRODUCTION

The analysis of the temporal dependence of luminescence (fluorescence, phosphorescence, scattering, etc.) is a topic of great interest in many disciplines, ranging from fundamental photophysical studies to biomedical imaging applications.^1–3^ Traditionally, two different approaches have been used to access temporal information: frequency modulation and pulsed excitation. The analysis of the latter has often relied on time-resolved recording of the resulting emission and fitting a decay model to the observed temporal profile.^4–6^ Recently, phasor analysis,^7^ also known as A-B plot^8^ or polar plot^9^ analysis, an alternative approach rooted in the analysis of sinusoidally modulated signals,^10^ has emerged and gained in popularity. It can be applied to signals resulting from periodic pulsed excitation and recorded with a variety of detector types and offers a number of advantages to the user, including an intuitive visualization of luminescence lifetime information in the data and rapid computation.

The phasor analysis of signals recorded with time-correlated single-photon counting (TCSPC) hardware, which precisely time-stamps each photon arrival with respect to the excitation pulse, is well established^11,12^ and relies on the fact that the phasor of the *recorded decay* (after correction of the effects of pile-up and electronic response function) can be considered essentially identical to that of the *emitted signal* up to a rotation and/or dilation in the complex plane. However, theoretical results for systems using either sparse sampling or lower photon arrival time resolution (such as time-gated or integrating detectors) are much more limited.^13–16,39^ In particular, a number of subtleties can arise when the time-gating scheme involves *overlapping gates* or, on the contrary, *non-adjacent gates*. In both quasi-continuous (TCSPC) and discrete (time-gated or integrated) acquisition modalities, cases of partial coverage of the laser period (“truncated” decays) or offset location of the excitation pulse within the recording time window (decay offset) further affect phasor calculation. With the advent of new detectors with diverse gating schemes, and in particular studies bridging the *in vitro* and *in vivo* realms and involving results obtained with diverse technologies and in different conditions,^17^ it appears important to investigate the modifications to standard phasor analysis brought about by this type of data.

This article focuses on the results for the phasor of *periodic single-exponential decays* (PSEDs), with brief mention of their extension to linear combinations of PSEDs or more general decays. The article is organized as follows: Sec. II introduces basic concepts and definitions regarding gated (as well as ungated) decays encountered in the luminescence lifetime experiment involving periodic excitation. The section ends with a short review of different examples of the modified PSEDs used throughout this article. Section III provides a concise reminder of the phasor analysis concepts used in the remainder of this article, in particular the properties of the phasor of convolution products, with special attention to the phasor of decays with finite sampling (which we refer to as “discrete” phasor). The section ends with analytical expressions for the phasor of time-gated PSEDs for the examples introduced in Sec. II and discusses the basic properties of the loci of these phasors (referred to here as “Single-Exponential Phasor Locus” curves or SEPL, pronounced “sepal,” as seems appropriate considering the diversity of shapes adopted by the curves studied in this article). In Sec. IV, the effect of a *decay offset*, which is non-trivial for discrete decays, and, in Sec. V, the effect of *decay truncation* on the phasor of PSEDs is studied. Section VI provides a brief overview of the influence of the *gate profile* (that is, a profile different from the square gate used as an illustration throughout the article) on previous results. Section VII discusses extensions of the standard phase and modulus lifetime definitions for some of the cases discussed in Secs. III–VI.

These elementary results being established, Sec. VIII examines modifications to *phasor calibration* in the different situations described previously, with the goal to map these different situations to a few “canonical” ones, in order to facilitate data interpretation. In particular, we investigate the effect of standard phasor calibration in situations where the SEPL cannot be mapped to a canonical situation, in both gated and ungated cases. The results of Sec. VIII are what may be of most interest to a casual reader, the preceding ones preparing the theoretical ground for it. It is possible to read it without prior knowledge of the preamble to get a gist of the results established in this work.

Section IX, which briefly discusses a few recently published studies in light of the previous results, may help better grasp when their application is important.

**FIG. 1.**
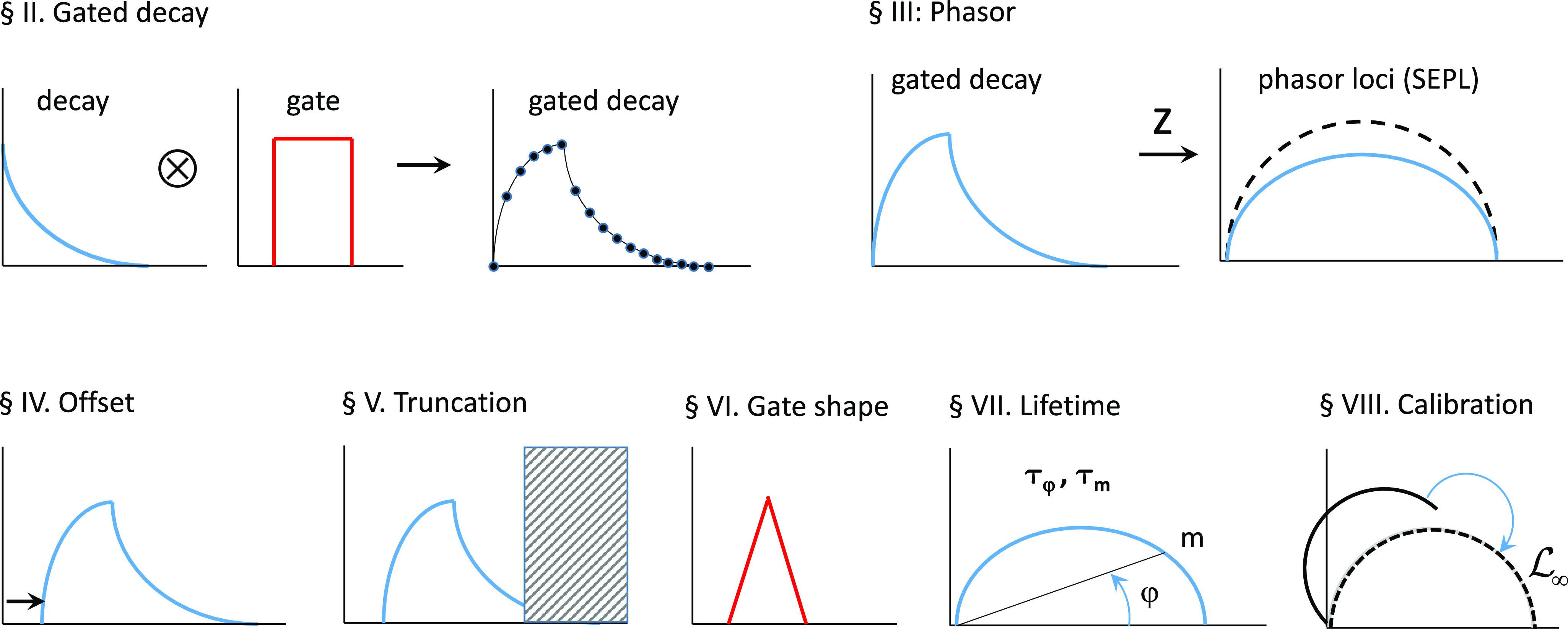
Graphical overview of this article. The general topic of each section is schematically illustrated for easy reference.

A graphical overview of the content of Secs. II–IX is provided in Fig. 1 for reference.

Finally, Sec. X summarizes the concepts introduced in this article and provides general recommendations.

As a final note, let us clarify that this article does not intend to provide either an introduction to phasor analysis or a discussion of its merits and pitfalls, all of which have been discussed in many excellent publications (for instance, Refs. 18 and 19) and are recommended reading. However, it does not assume prior knowledge about any analytical aspects of phasor analysis. In order to provide a self-contained study and the necessary definitions, some results previously derived by others will inevitably be repeated, with due reference to the literature. Most calculations are presented in an abridged form in the appendixes provided in the supplementary material. A list of notations is provided in Tables I and II, with mention of where they are defined in the text. Finally, links to free software used in this work and raw data available are provided at the end of this article.

**TABLE I.** Different loci of phasors of PSEDs (SEPL) discussed in this article. Index notations: ∞ indicates a continuous phasor, *N* indicates a discrete phasor, and [*W*] indicates a square-gated phasor.

	Discussed	Equation	Type of
SEPL	in Section	number	curve
$L_{\infty }$	III B 2	(70)	Universal semicircle
$L_{\left[W\right]}$	III B 3	(75)	Rotated, dilated semicircle
$L_{N}$	III C 3	(99)	Circular arc
$L_{N\left[W\right]}$	III C 4	(103)	Complex curve unless *W* = *qθ*

**TABLE II.** Notations.

Symbol	Description	Defined in equations
$\left\lfloor x\right\rfloor$	Floor function	(4)
$\left\lceil x\right\rceil$	Ceiling function	(105)
$x\left[T\right]$	Modulo operation	(18)
$H\left(t\right)$	Heaviside function	(8)
$f_{T}\underset{T}{*}g_{T}\left(t\right)$	Cyclic convolution product of two *T*-periodic functions	(9)
$I_{T}\left(t\right)$	*T*-periodic instrument response function (IRF)	(15)
$\Lambda_{\tau }\left(t\right)$	Normalized exponential function	(16)
$\Lambda_{\tau ,T}\left(t\right)$	Normalized *T*-periodic exponential function (PSED)	(17)
$\Lambda_{\tau ,T|t_{0}}\left(t\right)$	Normalized PSED with offset (Dirac excitation)	(120)
$\Psi_{\tau ,\tau_{\times },T}\left(t\right)$	Convolution of 2 *T*-PSEDs	(20) and (27)
${\bar{\Gamma }}_{W,nT}\left(t\right)$	Mirrored *nT*-periodic gate function of width *W*	(35)
${\bar{\Pi }}_{W,nT}\left(t\right)$	Mirrored *nT*-periodic square-gate function of width *W*	(37)
$I_{T,W}\left(t\right)$	Square-gated (width *W*) *T*-periodic IRF	(43)
$\Lambda_{\tau ,T,W}\left(t\right)$	Square-gated (*W*) *T*-PSED (Dirac excitation)	(47)
$\Lambda_{\tau ,T,W|t_{0}}\left(t\right)$	Square-gated (*W*) *T*-PSED with offset (Dirac excitation)	(121)
$\Psi_{\tau ,\tau_{\times },T,W}\left(t\right)$	Square-gated (*W*) *T*-PSED (single-exponential excitation)	(48)
$z\left[S_{T}\right]$	Cyclic phasor of *T*-periodic function *S*_*T*_	(66) and (67)
$\zeta_{f}\left(\tau \right)$	Continuous phasor of ungated PSED (Dirac excitation)	(70)
$z_{\left[W\right]}\left[\Lambda_{\tau ,T}\right]$	Continuous phasor of square-gated PSED (Dirac excitation)	(75)
$z_{N}\left[S_{T}\right]$	Discrete cyclic phasor of *T*-periodic function *S*_*T*_	(88), (91), and (92)
$z_{N\left[W\right]}\left[\Lambda_{\tau ,T}\right]$	Discrete phasor of square-gated PSED (Dirac excitation)	(103)
$\left\|S\left(t\right)\right\|,\left\|S\right\|$	Integral over ]−∞,∞[ of function *S*	(53)
${\left\|S_{T}\left(t\right)\right\|}_{T},{\left\|S_{T}\right\|}_{T}$	Integral over [0,*T*] of *T*-periodic function *S*_*T*_	(60)
${\left\|S_{T}\left(t_{p}\right)\right\|}_{N},{\left\|S_{T}\right\|}_{N}$	Discrete version of ${\left\|S_{T}\left(t\right)\right\|}_{D}$ over [0,*D*], D: record duration	(88) and (92)

## II. TIME-GATED PERIODIC DECAYS

### A. Periodic decays

#### 1. Excitation pulse, pure decay, and emitted signal

Steady-state *T*-periodic excitation of a system results in a *T*-periodic emitted signal, whose temporal profile is the sum of the signals excited by individual pulses. Let *ε*_0_(*t*) be the *signal emitted by the system after a single excitation pulse* $x_{0}\left(t\right)$, the latter being generated nominally at time *t*_0_. Since the excitation pulse will in general have a non-instantaneous profile, the definition of time *t*_0_ is somewhat arbitrary. To fix ideas, we will make the reasonable assumption that $x_{0}\left(t\right)$ reaches a maximum at a well-defined time, which we will call *t*_0_. Initially, we will assume *t*_0_ = 0 for simplicity but examine the general case in Sec. IV.

Introducing $F_{0}\left(t\right)$, the *response of the sample to a single Dirac excitation pulse* $\delta \left(t\right)$, which we will refer to as the sample’s *pure decay*, the emitted signal *ε*_0_(*t*) after a *single* non-Dirac excitation pulse $x_{0}\left(t\right)$ is given by the convolution product

$$\varepsilon_{0}\left(t\right)=\overset{+\infty }{\underset{-\infty }{\int }}x_{0}\left(u\right)F_{0}\left(t-u\right)du=x_{0}*F_{0}\left(t\right).$$ (1)

By definition, $F_{0}\left(t\right)$ is equal to zero for *t* < 0 and, in general (but not necessarily), decays from a maximum value reached at *t*_*max*_ ≥ 0 to 0 as *t* → *∞*.

#### 2. T-periodic summation and periodic signal

The steady-state emitted *T*-*periodic* signal $\varepsilon_{0,T}\left(t\right)$ obtained by the superimposition of the responses to *infinitely many* excitation pulses separated by a period *T* is given by the *T*-*periodic summation*

$$\left\{\begin{array}{c} \varepsilon_{0,T}\left(t\right)=\overset{+\infty }{\underset{k=-\infty }{\sum }}\varepsilon_{k}\left(t\right)=\overset{n\left(t\right)}{\underset{k=-\infty }{\sum }}\varepsilon_{k}\left(t\right),\\ n\left(t\right)=\left\lfloor t/T\right\rfloor , \end{array}\right.$$ (2)

where we have introduced the definitions

$$\left\{\begin{array}{c} \varepsilon_{k}\left(t\right)=\overset{+\infty }{\underset{-\infty }{\int }}x_{k}\left(u\right)F_{0}\left(t-u\right)du=\varepsilon_{0}\left(t-kT\right),\\ x_{k}\left(t\right)=x_{0}\left(t-kT\right), \end{array}\right.$$ (3)

and the notation $\left\lfloor x\right\rfloor$ denotes the “lower” integer part of *x* (the floor function of programming languages),

$$\forall x\in R,\;x\in [n,n+1[,n\in Z\Rightarrow \;\left\lfloor x\right\rfloor =n,$$ (4)

while the index *T*, such as *ε*_0,*T*_ in Eq. (2), indicates that it is a *T*-periodic function, as will be the convention in the remainder of this article.

*ε*_*k*_(*t*) is the system’s response to the *k*th excitation pulse $x_{k}\left(t\right)$. The sum truncation on the rightmost side of Eq. (2) is due to the fact that a system cannot respond to an excitation that has not yet taken place at time *t*.

Equation (2) can be rewritten as

$$\varepsilon_{0,T}\left(t\right)=x_{0,T}*F_{0}\left(t\right),$$ (5)

where the *T*-periodic excitation function $x_{0,T}\left(t\right)$ is defined by the *T*-periodic summation

$$x_{0,T}\left(t\right)=\overset{n\left(t\right)}{\underset{k=-\infty }{\sum }}x_{0}\left(t-kT\right).$$ (6)

As before, the sum is truncated at $k=n\left(t\right)=\left\lfloor t/T\right\rfloor$ as signals do not propagate back in time. For a Dirac pulse, $x_{0}\left(t\right)=\delta \left(t\right)$, the resulting *T*-periodic summation is a truncated *Dirac comb* (sometimes designated by the shah symbol *III*^20^),

$$\delta_{T}\left(t\right)=\overset{n\left(t\right)}{\underset{k=-\infty }{\sum }}\delta \left(t-kT\right).$$ (7)

An alternative way to write Eqs. (2) and (6) without the need to introduce explicitly the upper bound *n*(*t*) consists in writing *ε*_0_(*t*) and $x_{0}\left(t\right)$ as products of some function with the *Heaviside function* $H\left(t\right)$, where

$$H\left(t\right)=\left\{\begin{array}{c} 0ift<0\\ 1ift\geq 0. \end{array}\right.$$ (8)

#### 3. Cyclic convolution product

Introducing $f_{T}\underset{T}{*}g_{T}$, the periodic version of the convolution product of two *T*-periodic functions $f_{T}\left(t\right)$ and $g_{T}\left(t\right)$ [*circular* or *cyclic convolution*, see Appendix C.1, Eq. (C2)],

$$f_{T}\underset{T}{*}g_{T}\left(t\right)=\overset{T}{\underset{0}{\int }}du\;f_{T}\left(u\right)g_{T}\left(t-u\right),$$ (9)

we can rewrite Eq. (5) as

$$\varepsilon_{0,T}\left(t\right)=x_{0,T}\underset{T}{*}F_{0,T}\left(t\right),$$ (10)

where we have introduced the *T*-periodic summation $F_{0,T}\left(t\right)$ of the pure decay, $F_{0}\left(t\right)$, response of the sample to a Dirac excitation, defined by

$$F_{0,T}\left(t\right)=\overset{+\infty }{\underset{k=-\infty }{\sum }}F_{0}\left(t-kT\right).$$ (11)

These two definitions (*T*-summation and cyclic convolution product) will be used extensively throughout this work. The connection with non-periodic functions and the regular convolution product is provided by the following identities (derived in Appendix C.1):

$$\begin{array}{ll} & f_{T}\underset{T}{*}g_{T}\left(t\right)=g_{T}\underset{T}{*}f_{T}\left(t\right)=f*g_{T}\left(t\right)=f_{T}*g\left(t\right)\\ & \;=g_{T}*f\left(t\right)=g*f_{T}\left(t\right)={\left(f*g\right)}_{T}\left(t\right), \end{array}$$ (12)

where *f* and *g* are arbitrary functions with support over R and *f*_*T*_ and *g*_*T*_ are their *T*-periodic summations.

#### 4. Electronic response function

The emitted *T*-periodic signal $\varepsilon_{0,T}\left(t\right)$ of Eq. (10) is generally detected by a series of instruments (detectors, electronics, etc.) with a characteristic response $E\left(t\right)$ to a hypothetical instantaneous incident signal $\delta \left(t\right)$, its so-called *electronic response function* (ERF).^21^ The resulting *T*-periodic recorded signal $S_{T}\left(t\right)$ is given by the convolution of the (non-periodic) ERF with the periodic emitted signal,

$$S_{T}\left(t\right)=E*\varepsilon_{0,T}\left(t\right)=\overset{+\infty }{\underset{-\infty }{\int }}du\;E\left(u\right)\varepsilon_{0,T}\left(t-u\right)=E_{T}\underset{T}{*}\varepsilon_{0,T}\left(t\right).$$ (13)

This equation introduces $E_{T}\left(t\right)$, the *T*-periodic summation of $E\left(t\right)$, and rewrites the recorded signal as a cyclic convolution product.

Note that at this stage, we do not specify whether the detector is time-gated or not. We will make this distinction in Sec. II B.

#### 5. Instrument response function

Equation (13) can be rewritten as

$$\begin{array}{ll} S_{T}\left(t\right) & =E_{T}\underset{T}{*}\left(x_{0,T}\underset{T}{*}F_{0,T}\right)\left(t\right)\\ & =\left(E_{T}\underset{T}{*}x_{0,T}\right)\underset{T}{*}F_{0,T}\left(t\right)\\ & =I_{T}\underset{T}{*}F_{0,T}\left(t\right). \end{array}$$ (14)

Equation (14) introduces $I_{T}\left(t\right)$_,_ the *T*-periodic *instrument response function* (IRF), equal to the cyclic convolution of the *T*-periodic *excitation* function $x_{0,T}\left(t\right)$ with the *T*-periodic summation of the *electronic response* function, $E_{T}\left(t\right)$,

$$I_{T}\left(t\right)=E_{T}\underset{T}{*}x_{0,T}\left(t\right).$$ (15)

In other words, the instrument response function $I_{T}\left(t\right)$ incorporates the details of the excitation part of the optical setup (including the laser source temporal profile) and those of the detection part (including the electronic finite response time) in a single function, as is well known.^21^ While Eq. (15) is useful to understand the contribution of excitation and detection in the IRF, $I_{T}\left(t\right)$ is, in practice, the only measurable quantity in an experimental system. Equation (14) shows that it is all that is needed to account for the recorded signal $S_{T}\left(t\right)$ if the source signal functional form $F_{0}\left(t\right)$ [or its *T*-periodic summation, $F_{0,T}\left(t\right)$] is known, a property that is at the core of the convolution properties of continuous phasors, as discussed in Sec. III.

#### 6. Examples of periodic decays

##### a. Example 1: Dirac IRF and single-exponential decay.

To illustrate the difference between a single-period response and the summed, *T*-periodic response, it is useful to consider the case of a Dirac IRF, *δ*(*t*), and a normalized *single-exponential decay with lifetime τ*, $\Lambda_{\tau }\left(t\right)$, whose analytical expression is easily computed, starting from

$$\left\{\begin{array}{c} I\left(t\right)=\delta \left(t\right),\\ F_{0}\left(t\right)=\Lambda_{\tau }\left(t\right)≜\frac{1}{\tau }e^{-t/\tau }H\left(t\right), \end{array}\right.$$ (16)

where *H*(*t*) is the Heaviside function and both $I\left(t\right)$ and $\Lambda_{\tau }\left(t\right)$ have an integral of 1 over ]−∞, +∞[ (symbol ≜ will be used to indicate that the definition of the term to the left of that symbol is given by the expression on the right).

It is easy to verify that the corresponding *T*-periodic decay [$F_{0,T}\left(t\right)$ in Eq. (2) or (5)] is [see Appendix D, Eq. (D1)]

$$\Lambda_{\tau ,T}\left(t\right)≜\frac{1}{\tau \left(1-e^{-T/\tau }\right)}e^{-\left(t-\left\lfloor t/T\right\rfloor T\right)/\tau }=\frac{1}{\tau \left(1-e^{-T/\tau }\right)}e^{-t\left[T\right]/\tau },$$ (17)

where we have introduced the *modulo T* operation, $t\left[T\right]$,

$$t\left[T\right]=t-\left\lfloor t/T\right\rfloor \;T\;\in [0,T[.$$ (18)

In other words, in this simple case, the total emitted signal is simply a scaled (and *T*-periodic) version of the original signal $\Lambda_{\tau }\left(t\right)$ over [0, *T*[ ($t\in \;[0,T[\Rightarrow t\left[T\right]=t$). Its integral over [0, *T*] is equal to 1.

##### b. Example 2: Single-exponential IRF and single-exponential decay.

Another simple example is provided by a single-exponential IRF with time constant *τ*_×_ convolved with a single-exponential decay with lifetime *τ*,

$$\left\{\begin{array}{c} I\left(t\right)=\Lambda_{\tau_{\times }}\left(t\right)=\frac{1}{\tau_{\times }}e^{-t_{\times }/\tau }H\left(t\right),\\ F_{0}\left(t\right)=\Lambda_{\tau }\left(t\right)=\frac{1}{\tau }e^{-t/\tau }H\left(t\right). \end{array}\right.$$ (19)

The result of the convolution with the *T*-periodic IRF is [see Appendix D, Eq. (D3)]

$$\begin{array}{ll} \Psi_{\tau ,\tau_{\times },T}\left(t\right)≜I_{T}*F_{0}\left(t\right) & =I_{T}\underset{T}{*}F_{0,T}\left(t\right)\\ & =\Lambda_{\tau_{\times },T}\underset{T}{*}\Lambda_{\tau ,T}\left(t\right)\\ & =\frac{\tau \Lambda_{\tau ,T}\left(t\right)-\tau_{\times }\Lambda_{\tau_{\times },T}\left(t\right)}{\tau -\tau_{\times }} \end{array}$$ (20)

for *τ* ≠ *τ*_×_, where $\Lambda_{\tau ,T}\left(t\right)$ is the *T*-periodic function defined in Eq. (17). A distinct formula [Eq. (D6)] needs to be used when *τ* = *τ*_×_. When *τ*_×_ → 0, we recover Eq. (17) obtained in the case of a *T*-periodic Dirac IRF. Some properties of these functions are discussed in Appendix D. In particular, its integral over [0, *T*] is equal to 1.

#### 7. General representation of periodic decays

In the case of arbitrary IRFs, the results obtained for the simple example above can be generalized as discussed next.

##### a. Decomposition in bases of exponential functions.

In the general case, an IRF $I\left(t\right)$ can always be expressed as the Laplace transform of some function $g\left(k\right)$,

$$I\left(t\right)=\overset{\infty }{\underset{0}{\int }}dk\;g\left(k\right)e^{-kt}.$$ (21)

This integral transform can also be rewritten as^22^

$$I\left(t\right)=\overset{\infty }{\underset{0}{\int }}d\tau \;\eta_{0}\left(\tau \right)e^{-t/\tau },$$ (22)

where $\eta_{0}\left(\tau \right)$ is the weight function of $I\left(t\right)$ in the basis of single-exponential functions ${\left\{e^{-t/\tau }\right\}}_{\tau >0}$. As discussed in Ref. 22, $\eta_{0}\left(\tau \right)$ need not be positive and, therefore, cannot in general be considered as a probability density of lifetimes (which would also require it to be normalized).

An alternative representation is given by

$$I\left(t\right)=\overset{\infty }{\underset{0}{\int }}d\tau \;\xi_{0}\left(\tau \right)\frac{e^{-t/\tau }}{\tau }=\overset{\infty }{\underset{0}{\int }}d\tau \;\xi_{0}\left(\tau \right)\Lambda_{\tau }\left(t\right),$$ (23)

where the basis of decomposition is comprised of the *normalized* exponential functions ${\left\{\Lambda_{\tau }\left(t\right)\right\}}_{\tau >0}$ defined in Eq. (16) and $\xi_{0}\left(\tau \right)$ is the weight function of $I\left(t\right)$ in this basis. This latter decomposition leads to simpler notations in some of the later results. Note that, like $\eta_{0}\left(\tau \right)$, $\xi_{0}\left(\tau \right)$ need not be positive.

All these representations are related by

$$\xi_{0}\left(\tau \right)=\tau \eta_{0}\left(\tau \right)=\frac{1}{\tau }g\left(\frac{1}{\tau }\right).$$ (24)

The *T*-periodic summation of $I\left(t\right)$ can thus be expressed as

$$\begin{array}{ll} I_{T}\left(t\right) & =\overset{\left\lfloor t/T\right\rfloor }{\underset{n=-\infty }{\sum }}I\left(t-nT\right)\\ & =\overset{\left\lfloor t/T\right\rfloor }{\underset{n=-\infty }{\sum }}\overset{\infty }{\underset{0}{\int }}d\tau \;\eta_{0}\left(\tau \right)e^{-\left(t-nT\right)/\tau }\\ & =\overset{\infty }{\underset{0}{\int }}d\tau \;\eta_{0}\left(\tau \right)\frac{e^{-t\left[T\right]/\tau }}{1-e^{-T/\tau }}\\ & =\overset{\infty }{\underset{0}{\int }}d\tau \;\eta_{0}\left(\tau \right)\tau \Lambda_{\tau ,T}\left(t\right)\\ & =\overset{\infty }{\underset{0}{\int }}d\tau \;\xi_{0}\left(\tau \right)\Lambda_{\tau ,T}\left(t\right). \end{array}$$ (25)

Comparing the last terms of Eqs. (23) and (25), it is clear that the representation of the original function $I\left(t\right)$ in terms of $\xi_{0}\left(\tau \right)$ leads to a simpler form for its *T*-periodic summation $I_{T}\left(t\right)$, the two expressions appearing identical except for a replacement of $\Lambda_{\tau }\left(t\right)$ in Eq. (23) by its *T*-periodic summation $\Lambda_{\tau ,T}\left(t\right)$ in Eq. (25).

##### b. Application to PSEDs convolved with an arbitrary IRF.

Calculations similar to those detailed in Appendix D lead to the following formula for the convolution of an arbitrary *T*-periodic excitation function $I_{T}\left(t\right)$ and a PSED $\Lambda_{\tau_{0},T}\left(t\right)$,

$$\begin{array}{ll} I_{T}\underset{T}{*}\Lambda_{\tau_{0},T}\left(t\right) & =\overset{\infty }{\underset{0}{\int }}d\tau \;\xi_{0}\left(\tau \right)\;\Lambda_{\tau ,T}\underset{T}{*}\Lambda_{\tau_{0},T}\left(t\right)\\ & =\overset{\infty }{\underset{0}{\int }}d\tau \;\xi_{0}\left(\tau \right)\Psi_{\tau ,\tau_{0},T}\left(t\right)\\ & =\overset{\infty }{\underset{0}{\int }}d\tau \;\xi_{0}\left(\tau \right)\frac{1}{\tau -\tau_{0}}\left(\tau \Lambda_{\tau ,T}\left(t\right)-\tau_{0}\Lambda_{\tau_{0},T}\left(t\right)\right). \end{array}$$ (26)

When $I\left(t\right)$ is a single-exponential function with time constant *τ*_×_, $\xi_{0}\left(\tau \right)=\delta \left(\tau -\tau_{\times }\right)$ and one recovers Eq. (20). Once again, this formula illustrates the advantage of using Eq. (23) to represent $I\left(t\right)$ since both formulas are identical save for the replacement of $\Lambda_{\tau }\left(t\right)$ in Eq. (23) by $\Psi_{\tau ,\tau_{0},T}\left(t\right)$ in Eq. (26).

Note that $\Psi_{\tau ,\tau_{0},T}\left(t\right)$ can be itself rewritten as

$$\left\{\begin{array}{c} \Psi_{\tau ,\tau_{0},T}\left(t\right)=\overset{\infty }{\underset{0}{\int }}d\lambda \;p_{\tau ,\tau_{0}}\left(\lambda \right)\Lambda_{\lambda ,T}\left(t\right),\\ p_{\tau ,\tau_{0}}\left(\lambda \right)=\frac{1}{\tau -\tau_{0}}\left(\delta \left(\lambda -\tau \right)-\delta \left(\lambda -\tau_{0}\right)\right), \end{array}\right.$$ (27)

which illustrates that, due to the presence of a negative sign, the weight function cannot in general be interpreted as a probability density function of lifetimes.^22^

### B. Time-gated or binned periodic signal

In the previous discussion, we emphasized the fact that details of the excitation and detection processes could be encompassed in a single IRF. While this can be convenient, it is also some time useful to separate out some aspects of the data acquisition process, especially when these aspects can be experimentally controlled. This is, in particular, the case of the gate duration (or more generally, gate shape) in a gated detection scheme or the bin size or timing resolution in a time-tagged detection system. Separating the effect of gating or binning on the recorded signal thus allows studying their influence on data analysis. In practice, although the experimental situations are different, both gating and binning can be treated by the same simple formalism. The purpose of this section is to elaborate this point. We first briefly discuss the different possible experimental situations, before introducing the formalism allowing their general analytical description.

#### 1. Detector types

Time-gating can be implemented in different ways depending on which detector is considered. We will distinguish between integrating and photon-counting detectors.

An example of a (time-gated) integrating detector still commonly found at the time of this writing is an intensified camera,^1,23^ in which the gain of the camera’s intensifier is modulated (either sinusoidally or turned on and off) periodically during the overall integration time by a camera. To a good approximation, in the case of a moderate incident signal and in a range of intensifier gain values that depends on the specific detector, the signal recorded by such detectors is proportional to the applied gain (some secondary effects such as gain saturation at high intensity can come into play and would need to be corrected for). In the case of such a time-gated integrating detector, the temporal variation of the gain can be identified with the gate shape discussed later in the text.

Photon-counting detectors come in many different flavors. Detectors such as single-photon avalanche diodes (SPADs), working in the so-called Geiger mode, provide a binary response (0 or 1) to an incoming instantaneous photon flux (they can detect a single photon at a time and two or more photons arriving within the duration of the avalanche are registered as a single event).^24^ By contrast, silicon photomultipliers (SiPMs)^25^ or hybrid photodetectors (HPDs)^26^ equipped with appropriate electronics are capable of actually counting the number of impinging photons during each detection event. All these detectors are capable, with the appropriate electronics, to precisely time-tag each event with respect to a reference pulse (the so-called time-correlated single-photon counting approach, or TCSPC). Alternatively, either by design of the detector or the associated electronics, they can provide information on the finite interval of time (the “gate”), with respect to a reference pulse, during which the detection event took place. In effect, a detector working in this manner will, by accumulation over time, count the number of photons arriving during a specific window of time with respect to a reference pulse. If the gate is defined by an applied electronic signal, it will in general have a shape governed by the response of the electronics, while if it is defined digitally (as, for example, in the FLIMbox approach^13^), the gate shape will be essentially “square” and amount to time binning of the equivalent time-tag information. In this respect, time binning of time-tagged data can be viewed as a form of digital time-gating and considered as a special case of time-gating, which is the perspective used in the remainder of this paper.

It is worth mentioning that, in addition to the previous detector specificities, counting electronics having their own limitation, in particular, a maximum counter value *q* before data needs to be read out. Examples of very different values for *q* can be found in the literature. For instance, the SPAD array used in Ref. 27 is characterized by *q* = 255, while SwissSPAD^28^ and SwissSPAD 2^29^ are characterized by *q* = 1. To compensate for this type of limitations, repeated measurements of finite duration (a “frame” encompassing *L* gates) can be performed and summed up to form an “image” comprised of *F* frames. The combination of all these characteristics results in general in signal saturation equivalent to the well-known pile-up effect caused by detector dead-time, which needs to and can be corrected.^16,30^ In the remainder of this article, we will assume that the corrected (or non-saturated) time-gated decays are used.

#### 2. Gate profile

In all cases, the detection efficiency of the detector can be modeled by a *gate function* Γ_*s*,*W*_(*t*) with finite support [*s*, *s* + *W*] such that

$$\Gamma_{s,W}\left(t\right)\;\left\{\begin{array}{cc} =0\; & ift<s\\ \in ]0,1]\; & if\in \left[s,s+W\right]\\ =0\; & ift>s+W, \end{array}\right.$$ (28)

where *s* is the gate’s *offset* (with respect to a reference trigger, generally corresponding to the excitation pulse) and *W* is its *width*. The hypothesis of a finite support is appropriate for the specific examples discussed in this study but can in principle be relaxed to allow for more general integration or modulation schemes or detector types, including sinusoidal ones relevant to frequency modulation techniques. In these cases, the support of the gate function covers the whole laser period (or multiple thereof, as discussed later in this section), and the notion of “gate width” becomes useless.

The simplest example is a gate function proportional to the *boxcar* function Π_*s*,*W*_(*t*),

$$\Pi_{s,W}\left(t\right)=\left\{\begin{array}{cc} 0\; & ift<s\\ 1\; & ift\in \left[s,s+W\right]\\ 0\; & ift>s+W. \end{array}\right.$$ (29)

In general, the detector’s response to the applied voltage swing, or the voltage swing itself, is not instantaneous, and the boxcar function may need to be replaced by a function with “rounder” edges. Herein, we will limit ourselves to the boxcar model (which we will henceforth refer to as a *square gate*) as the results derived with this model are minimally affected by small departures from it. Numerical experimentations with other gate shapes (e.g., triangle, sawtooth, logistic edge, or custom gate) can be performed using the accompanying *Phasor Explorer* software (Appendix F) and are discussed in Sec. VI.

Because gates are generally synchronized with respect to the excitation pulse, we will be interested in periodic versions of Γ_*s*,*W*_(*t*),

$$\Gamma_{s,W,T_{G}}\left(t\right)=\Gamma_{s,W}\left(t\left[T_{G}\right]\right)\;\left\{\begin{array}{cc} =0\; & ift\left[T_{G}\right]<s\\ \in \left[0,1\right]\; & ift\left[T_{G}\right]\in \left[s,s+W\right]\\ =0\; & ift\left[T_{G}\right]>s+W, \end{array}\right.$$ (30)

where $s\in \left[0,T_{G}\right.$[ is again the start or *offset* of the gate with respect to the reference trigger, used to define time 0 (*vide supra*). *T*_*G*_ = *nT* is the *gate period*, which we will allow to be equal to any multiple of the laser period (*n* ≥ 1) to account for situations that may require *n* > 1. For instance, if the gate width *W* > *T*, it does not make sense to re-open the gate after one laser period *T*, as the previous one will still not be closed. Another possible reason could be that the detector gating electronics is not capable of responding at the laser repetition rate, forcing a decimation of the incoming laser triggers. In the particular case of a square gate [Eq. (29)], a *nT*-periodic version can also be defined as

$$\Pi_{s,W,nT}\left(t\right)=\Pi_{s,W}\left(t\left[nT\right]\right)=\left\{\begin{array}{cc} 0\; & ift\left[nT\right]<s\\ 1\; & ift\left[nT\right]\in \left[s,s+W\right]\\ 0\; & ift\left[nT\right]>s+W. \end{array}\right.$$ (31)

#### 3. Gate offset

Experimentally, gated data are acquired for different values of the gate offset *s*, ${\left\{s_{k}\right\}}_{1\leq k\leq N}$. Formulas derived next will consider arbitrary values of *s*, the specific case of a finite number of offsets (and therefore, gates) being discussed separately when needed.

Note that definition Eq. (28) and the following assume that the offset *s* in Γ_*s*,*W*_(*t*) represents the beginning of the gate’s support (that is, the interval over which the gate value is non-zero). It is possible to extend this definition to account for cases where the gate’s offset is not exactly known and different from the index *s* used to refer to it,

$$\Gamma_{s,W,s_{0}}\left(t\right)=\left\{\begin{array}{cc} 0\; & ift<s+s_{0}\\ \left.\left.\in \right]0,1\right]\; & ift\in \left[s+s_{0},s+s_{0}+W\right]\\ 0\; & ift>s+s_{0}+W. \end{array}\right.$$ (32)

The unknown offset delta, *s*_0_, amounts to an IRF offset −*s*_0_ as will be discussed in Sec. II B 4. It is possible to extend this concept of gate offset to gate profiles with support covering the whole gate period.

#### 4. Gated signal and mirrored gate

The signal accumulated during a gate starting at time *s*, $S_{T,W}\left(s\right)$, is given by

$$S_{T,W}\left(s\right)=\int_{0}^{nT}\Gamma_{s,W,nT}\left(t\right)S_{T}\left(t\right)\;dt.$$ (33)

This function is clearly *T*-periodic, hence the index *T* in the previous notation. This equation can be rewritten as a cyclic convolution product by introducing the *nT*-periodic *mirrored gate function* of the gate function, ${\bar{\Gamma }}_{W,nT}\left(t\right)$, verifying

$${\bar{\Gamma }}_{W,nT}\left(s-t\right)=\Gamma_{s,W,nT}\left(t\right).$$ (34)

This identity is equivalent to

$${\bar{\Gamma }}_{W,nT}\left(t\right)=\Gamma_{0,W,nT}\left(-t\right)=\Gamma_{0,W,nT}\left(nT-t\right)=\Gamma_{0,W,nT}\left(-t\left[nT\right]\right)$$ (35)

and is a mirror image with respect to half the gate period *nT* of the gate function starting at *t* = 0.

For a square gate (boxcar) function as defined in Eq. (31), the *mirrored square-gate function* of width *W* and period *nT* is defined by

$${\bar{\Pi }}_{W,nT}\left(t\right)=\Pi_{0,W,nT}\left(nT-t\right)$$ (36)

or, explicitly,

$${\bar{\Pi }}_{W,nT}\left(t\right)=\Pi_{0,W,nT}\left(nT-t\right)=\left\{\begin{array}{c} 0ifnT-t<0\\ 1if0\leq nT-t\leq W\\ 0ifnT-t>W. \end{array}\right.$$ (37)

With definition Eq. (34) of the mirrored gate function, Eq. (33) reads

$$S_{T,W}\left(t\right)\;=\int_{0}^{nT}ds\;S_{T}\left(s\right){\bar{\Gamma }}_{W,nT}\left(t-s\right)={\bar{\Gamma }}_{W,nT}\underset{nT}{*}S_{T}\left(t\right),$$ (38)

where the cyclic convolution product is defined for a period *nT* and we have used the fact that $S_{T}\left(t\right)$ is also *nT*-periodic. The notation $\underset{nT}{*}$ specifies the period of the convolution product as *T*_*G*_ = *nT* > *W*, the gate period imposed by the gate width (as noted before, in most cases, *n* = 1).

Because the experimental signal accumulated during a single gate period is generally very small (less than one photon in the case of a photon-counting detector), the signals of several (*L*) gates separated by a duration *T*_*G*_ = *nT* are integrated to generate what we will designate henceforth as the *integrated gate signal S*_*T*,*W*_(*t*)_*L*_,

$$S_{T,W}{\left(t\right)}_{L}=\overset{L-1}{\underset{l=0}{\sum }}S_{T,W}\left(t+lT_{G}\right).$$ (39)

Using the *nT*-periodicity of the emitted signal $S_{T}\left(t\right)$, we have

$$S_{T,W}{\left(s\right)}_{L}=L\;S_{T,W}\left(s\right).$$ (40)

Since the two signals differ only by a constant experimental multiplication factor, which does not intervene in the results derived in this discussion, we will omit the distinction between both and henceforth only refer to $S_{T,W}\left(t\right)$. However, this multiplication factor would be necessary when considering effects such as shot noise.

As mentioned in Sec. II B 3, gate definition (28), which assumes a perfect knowledge of where the gate starts, might need to be modified into Eq. (32), which introduces an offset delta *s*_0_. Plugging this definition in Eq. (33),

$$\begin{array}{ll} S_{T,W}\left(s\right) & =\int_{0}^{nT}\Gamma_{s,W,s_{0},nT}\left(t\right)S_{T}\left(t\right)\;dt\\ & =\int_{0}^{nT}\Gamma_{s,W,s_{0},nT}\left(t+s_{0}\right)S_{T}\left(t+s_{0}\right)\;dt\\ & =\int_{0}^{nT}\Gamma_{s,W,nT}\left(t\right)\;\delta_{-s_{0}}\underset{T}{*}S_{T}\left(t\right)\;dt\\ & ={\bar{\Gamma }}_{W,nT}\underset{nT}{*}\delta_{-s_{0}}\underset{T}{*}S_{T}\left(t\right), \end{array}$$ (41)

where $\delta_{-s_{0}}\left(t\right)=\delta \left(t+s_{0}\right)$ is the Dirac function with offset *t*_0_ = −*s*_0_. In other words, an imperfect knowledge of where the gate starts can be incorporated into an additional IRF offset.

#### 5. Time-gated instrument response function

Combining Eq. (38) with Eq. (14), we obtain the following expression for the signal recorded by a setup employing a time-gated detection scheme:

$$\begin{array}{ll} S_{T,W}\left(t\right) & =I_{T}\underset{T}{*}F_{0,T}\left(t\right)\underset{nT}{*}{\bar{\Gamma }}_{W,nT}\left(t\right)\\ & =\left({\bar{\Gamma }}_{W,nT}\underset{nT}{*}I_{T}\right)\underset{T}{*}F_{0,T}\left(t\right)\\ & =I_{T,W}\underset{T}{*}F_{0,T}\left(t\right), \end{array}$$ (42)

which defines the *time-gated instrument response function* $I_{T,W}\left(t\right)$ as

$$I_{T,W}\left(t\right)≜{\bar{\Gamma }}_{W,nT}\underset{nT}{*}I_{T}\left(t\right),$$ (43)

demonstrating that gating is simply adding a product of convolution to the computation (note however that this product involves not the gate function itself but a mirrored version).

#### 6. Time-gated electronic response function

Using $I_{T}\left(t\right)$’s definition [Eq. (15)], we can further decompose $I_{T}\left(t\right)$ and write

$$I_{T,W}\left(t\right)={\bar{\Gamma }}_{W,nT}\underset{nT}{*}E_{T}\underset{T}{*}x_{0,T}\left(t\right),$$ (44)

which introduces the *time-gated electronic response function* $E_{T,W}\left(t\right)$,

$$E_{T,W}\left(t\right)≜{\bar{\Gamma }}_{W,nT}\underset{nT}{*}E_{T}\left(t\right).$$ (45)

This definition formally separates the gating part of the electronics response from any other electronics contributions, which is an appropriate representation if the acquisition electronics does contain two distinct signal processing stages. In cases where such a distinction does not exist or cannot be made explicitly, Eq. (44) for the IRF is replaced by a form identical to that of Eq. (15),

$$I_{T,W}\left(t\right)=E_{T,W}\underset{T}{*}x_{0,T}\left(t\right).$$ (46)

#### 7. Examples of square-gated PSEDs

##### a. Example 1: Square-gated PSEDs with Dirac IRF.

In the special case of a *T*-periodic Dirac IRF and a periodic single-exponential decay $\Lambda_{\tau ,T}\left(t\right)$ [Eq. (17)] and a boxcar gate with width *W* and period *nT* [Eq. (30)], we can explicitly compute the definition of the corresponding *square-gated* PSED, $\Lambda_{\tau ,T,W}\left(t\right)$, by evaluating Eq. (33). The result is

$$\Lambda_{\tau ,T,W}\left(t\right)≜\left\{\begin{array}{cc} \left(a\right)\; & \frac{1-e^{-\omega /\tau }}{1-e^{-T/\tau }}e^{-t\left[T\right]/\tau }+k\;ift\left[T\right]\in [0,T-\omega [\\ \left(b\right)\; & \frac{1-e^{-\left(\omega -T\right)/\tau }}{1-e^{-T/\tau }}e^{-t\left[T\right]/\tau }+k+1\;ift\left[T\right]\in \left[T-\omega ,T\left[\right.\right., \end{array}\right.$$

where

$$\left\{\begin{array}{c} k=\left\lfloor W/T\right\rfloor ,\;\\ \omega =W\left[T\right]=W-kT.\; \end{array}\right.$$ (47)

The introduction of *k* and *ω* accounts for cases where *W* > *T*, while that of $t\;\left[T\right]$ shifts the time argument back to the [0, *T*] interval. Note that with this definition, the integral of $\Lambda_{\tau ,T,W}\left(t\right)$ over [0, *nT*] is equal to *W*. In most experimental cases, *ω* = *W*, *k* = 0, and *n* = 1.

**FIG. 2.**
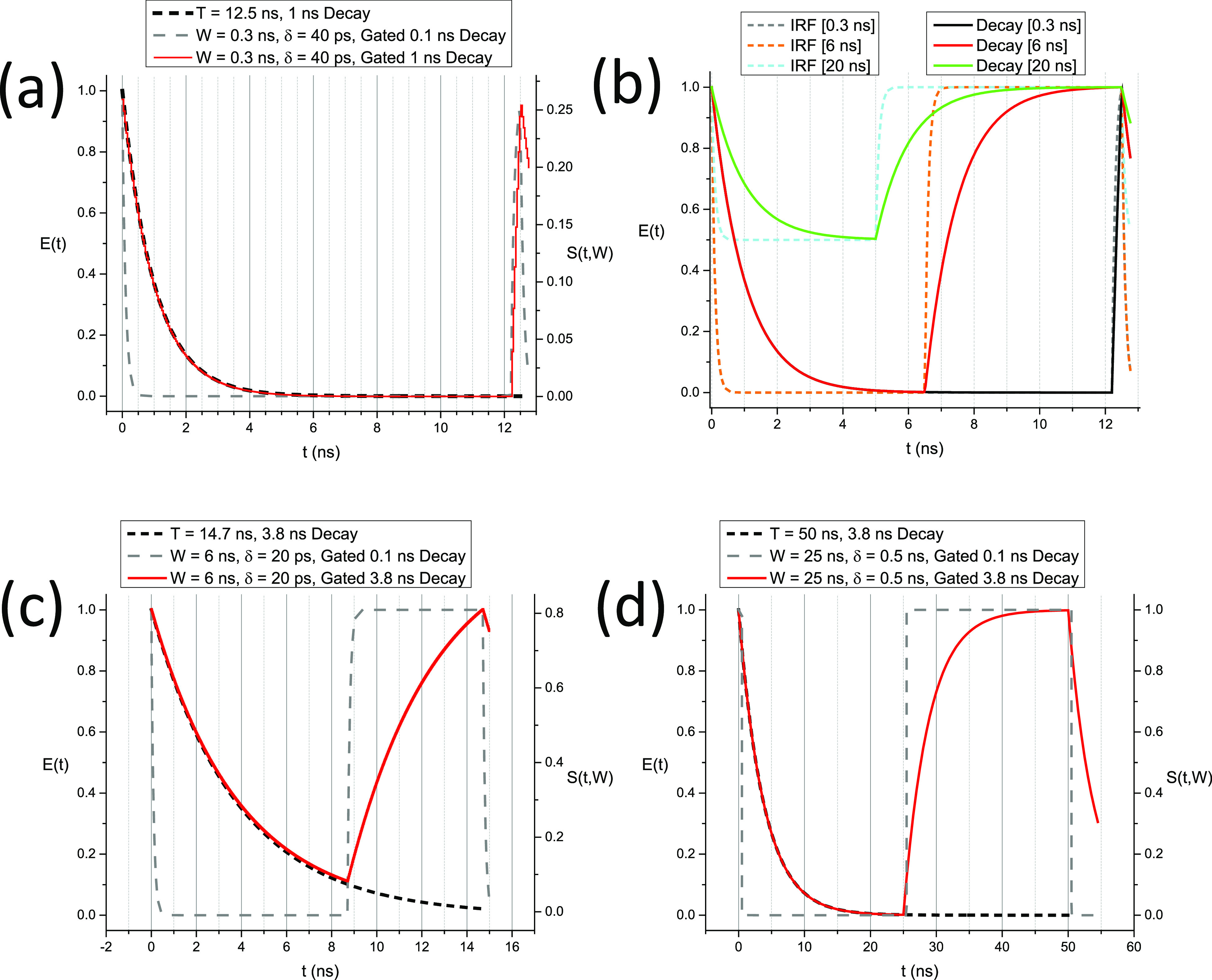
Examples of time-gated exponential decays. (a) Gated decays (*τ*_1_= 0.1 ns, representing the equivalent of an IRF, and *τ*_2_= 1 ns, laser period: 12.5 ns) with gate width *W* = 0.3 ns and gate separation (gate step) *δ* = 40 ps, corresponding to settings used in time-gated ICCD measurements performed in Ref. 15. The time-gated decay (red) is essentially identical to the ungated decay (black dashed curve), except at the end of the period where the rise time of the time-gated decay is of the order of *W* = 0.3 ns instead of being instantaneous. (b) Same decays (IRF: *τ*_1_= 0.1 ns, decay: *τ*_2_= 1 ns) but after time-gating with parameters *W*_1_ = 0.3 ns (black), *W*_2_ = 6 ns (red), or *W*_3_ = 20 ns (*δ* = 40 ps in all cases). The last two values correspond to characteristics of SwissSPAD 1 and 2 described in Refs. 28 and 29. For *W*_2_ = 6 ns, the main difference is at the end of the decay, where the instantaneous rise time is replaced by a mirror image of the decay of width *W*_2_. For *W*_3_ = 20 ns, the resulting decay is offset vertically due to the full period integration (*T* = 12.5 ns) common to all gates, and the instantaneous rise time is replaced by a mirror image of the decay of width *W*_3_ − *T* = 7.5 ns. (c) Gated decays (*τ*_1_= 0.1 ns, representing the equivalent of an IRF, and *τ*_2_= 3.8 ns, laser period: 14.7 ns) with gate width *W* = 6 ns and gate separation *δ* = 20 ps, corresponding to the settings used in the SwissSPAD measurements performed in Ref. 28. The time-gated decay (red curve) is essentially identical to the ungated decay (black dashed curve), except at the end of the period where the rise time of the time-gated decay is replaced by a mirror image of the decay of width *W* = 6 ns. (d) Gated decays (*τ*_1_ = 0.1 ns, representing the equivalent of an IRF, and *τ*_2_= 3.8 ns, laser period: 50 ns) with gate width *W* = 25 ns and gate separation *δ* = 500 ps, corresponding to the settings used in the SwissSPAD 2 measurements performed in Ref. 29. The time-gated decay (red curve) is essentially identical to the ungated decay (black dashed curve), except at the end of the period where the rise time of the time-gated decay is replaced by a mirror image of the decay of width *W* = 20 ns.

A few examples of square-gated PSEDs are represented in Fig. 2. The *Phasor Explorer* software accompanying this article (Appendix F) allows exploring other types of gate profiles (triangle, sawtooth, etc.), including user-defined ones for which analytical formulas might not be available or convenient to use. We will briefly look at the effect of other gate shapes in Sec. VI.

##### b. Example 2: Square-gated PSEDs with single-exponential IRF.

The evaluation of Eq. (33) where $S_{T}\left(t\right)=\Psi_{\tau ,\tau_{\times },T}\left(t\right)$, the ungated decay, is given by Eq. (20) and is outlined in Appendix D.6 and leads to the following result for the square-gated signal [Eq. (D20)]:

$$\begin{array}{ll} \Psi_{\tau ,\tau_{\times },T,W}\left(t\right) & =\frac{1}{\tau -\tau_{\times }}\left(\tau \Lambda_{\tau ,T,W}\left(t\right)-\tau_{\times }\Lambda_{\tau_{\times },T,W}\left(t\right)\right)\\ & =\left\{\begin{array}{c} \frac{1}{\tau -\tau_{\times }}\left(\tau \frac{1-u}{1-y}e^{-t\;\left[T\right]/\tau }-\tau_{\times }\frac{1-u_{\times }}{1-y_{\times }}e^{-t\;\left[T\right]/\tau_{\times }}\right)+k\;if\;t\;\left[T\right]\;\in [0,T-\omega [\\ \frac{1}{\tau -\tau_{\times }}\left(\tau \frac{1-uy^{-1}}{1-y}e^{-t\;\left[T\right]/\tau }-\tau_{\times }\frac{1-u_{\times }y_{\times }^{-1}}{1-y_{\times }}e^{-t\;\left[T\right]/\tau_{\times }}\right)+k+1\;if\;t\;\left[T\right]\;\in [T-\omega ,T[, \end{array}\right. \end{array}$$ (48)

where we have used the following notations:

$$\left\{\begin{array}{c} k=\left\lfloor W/T\right\rfloor ,\\ \omega =W\left[T\right]=W-kT,\\ y\left(\tau \right)=e^{-T/\tau },\;y_{\times }\left(\tau \right)=e^{-T/\tau_{\times }},\\ u\left(\tau \right)=e^{-\omega /\tau },\;u_{\times }\left(\tau \right)=e^{-\omega /\tau_{\times }}. \end{array}\right.$$ (49)

##### c. Square-gated PSEDs with general IRF.

Using the general representation of an IRF $I\left(t\right)$ given by Eq. (23), the square-gated PSED obtained with this excitation function is given by

$$\begin{array}{ll} {\bar{\Pi }}_{W,nT}\underset{nT}{*}I_{T}\underset{T}{*}\Lambda_{\tau ,\tau_{0},T}\left(t\right) & =\overset{\infty }{\underset{0}{\int }}d\tau \;\xi_{0}\left(\tau \right)\;{\bar{\Pi }}_{W,nT}\underset{nT}{*}\Psi_{\tau ,\tau_{0},T}\left(t\right)\\ & =\overset{\infty }{\underset{0}{\int }}d\tau \;\xi_{0}\left(\tau \right)\;\Psi_{\tau ,\tau_{0},T,W}\left(t\right). \end{array}$$ (50)

#### 8. General expression for time-gated PSEDs

For any other mirrored gate shape ${\bar{\Gamma }}_{W,nT}\left(t\right)$ and an IRF $I\left(t\right)$ given by Eq. (23) [or equivalently, its *T*-periodic version $I_{T}\left(t\right)$ given by Eq. (25)], the time-gated PSED is given by the generalization of Eq. (50),

$${\bar{\Gamma }}_{W,nT}\underset{nT}{*}I_{T}\underset{T}{*}\Lambda_{\tau ,\tau_{0},T}\left(t\right)=\overset{\infty }{\underset{0}{\int }}d\tau \;\xi_{0}\left(\tau \right)\;{\bar{\Gamma }}_{W,nT}\underset{nT}{*}\Psi_{\tau ,\tau_{0},T}\left(t\right).$$ (51)

## III. PHASOR OF PERIODIC DECAYS

### A. Definition and notations

#### 1. Phasor and Fourier transform

Phasor analysis is a well-documented approach to study fluorescence decays without having to resort to non-linear model fitting.^7,10,19,22^ The formalism found in most discussions in the literature uses non-periodic signals $S\left(t\right)$ (equal to zero for *t* < 0) and defines the phasor $z\left[S\right]\left(\;f\right)$ of signal *S* at harmonic frequency *f*, using infinite integrals

$$z\left[S\right]\left(\;f\right)=\frac{\overset{+\infty }{\underset{-\infty }{\int }}dt\;S\left(t\right)e^{i2\pi ft}}{\overset{+\infty }{\underset{-\infty }{\int }}dt\;S\left(t\right)}=\frac{\left\|S\left(t\right)e^{i2\pi ft}\right\|}{\left\|S\left(t\right)\right\|},$$ (52)

where we have introduced the notation $\left\|S\left(t\right)\right\|$ to denote the integral of $S\left(t\right)$ over ]−*∞*, +*∞*[,

$$\left\|S\left(t\right)\right\|=\overset{+\infty }{\underset{-\infty }{\int }}dt\;S\left(t\right).$$ (53)

Note that in most cases discussed in the following, the effective integration bounds are 0 and +∞ due to the fact that the decays we will consider in this work are equal to zero for *t* < 0.

From the ratiometric nature of definition (52), it results that the phasor is invariant by dilation,

$$\left(\forall a\in R^{*}\right),\;z\left[aS\right]=z\left[S\right].$$ (54)

This phasor definition is related to the *Fourier transform* $F\;\left[S\right]$ of *S*,

$$F\;\left[S\right]\left(\;f\right)=\overset{+\infty }{\underset{-\infty }{\int }}dt\;S\left(t\right)e^{-i2\pi ft},$$ (55)

defined for any value *f* ≥ 0, by the following relation:

$$z\left[S\right]\left(\;f\right)=\frac{{F\;}^{*}\left[S\right]\left(\;f\right)}{F\;\left[S\right]\left(0\right)},$$ (56)

where x^*^ indicates the complex conjugate of *x*.

As it is obvious from the above definitions, if $\left\|S\left(t\right)\right\|=1$ [i.e., if $S\left(t\right)$ is normalized], the relation between phasor and Fourier transform is further simplified into

$$\left\|S\left(t\right)\right\|=1\Rightarrow z\left[S\right]\left(\;f\right)=F^{*}\left[S\right]\left(\;f\right).$$ (57)

Because Eqs. (52) and (57) involve infinite integrals of non-periodic decay functions, the corresponding quantities are not directly accessible experimentally. It therefore appears important to show that this formalism can be replaced by an equivalent one involving finite integrals of periodic decays, which are experimentally accessible quantities.

#### 2. Cyclic phasor and Fourier series

Definition (52) of the phasor is in fact formally identical to an alternate definition involving *T*-periodic signals, which is more natural when dealing with experimental data. We will devote this section to establishing this connection.

When the phasor harmonic frequency *f* is a multiple of 1/*T*,

$$f=f_{n}=\frac{n}{T},\;n\in N\Rightarrow \left(\forall k\in Z\right)\;e^{i2\pi fkT}=1,$$ (58)

the numerator of Eq. (52) can be rewritten as

$$\begin{array}{ll} \overset{+\infty }{\underset{-\infty }{\int }}dt\;S\left(t\right)e^{i2\pi ft} & =\overset{+\infty }{\underset{k=-\infty }{\sum }}\overset{kT+T}{\underset{kT}{\int }}dt\;S\left(t\right)e^{i2\pi ft}\\ & =\overset{+\infty }{\underset{k=-\infty }{\sum }}\overset{kT+T}{\underset{kT}{\int }}dt\;S\left(t\right)e^{i2\pi f\left(t-kT\right)},\\ \left(u=t-kT\right) & =\overset{+\infty }{\underset{k=-\infty }{\sum }}\overset{T}{\underset{0}{\int }}du\;S\left(u+kT\right)e^{i2\pi fu}\\ & =\overset{T}{\underset{0}{\int }}du\;\left(\overset{+\infty }{\underset{k=-\infty }{\sum }}S\left(u+kT\right)\right)e^{i2\pi fu}\\ & =\overset{T}{\underset{0}{\int }}dt\;S_{T}\left(t\right)e^{i2\pi ft}, \end{array}$$ (59)

in which we have introduced $S_{T}\left(t\right)$, the *T*-periodic summation of $S\left(t\right)$ [Sec. II A 1, Eq. (6)]. As noted before, $S_{T}\left(t\right)$ is proportional to the signal measured in experiments, while the original signal $S\left(t\right)$ is generally not directly measurable.

Introducing the notation ${\left\|S_{T}\left(t\right)\right\|}_{T}$ for the integral of a *T*-periodic function $S_{T}\left(t\right)$ over a period *T* (we will occasionally use the simpler notation ${\left\|S_{T}\right\|}_{T}$ and omit the function’s argument),

$${\left\|S_{T}\left(t\right)\right\|}_{T}=\overset{T}{\underset{0}{\int }}dt\;S_{T}\left(t\right).$$ (60)

Equation (59) can be rewritten as

$$\left\|S\left(t\right)e^{i2\pi ft}\right\|={\left\|S_{T}\left(t\right)e^{i2\pi ft}\right\|}_{T}.$$ (61)

Similarly, it is trivial to verify that

$$\left\|S\left(t\right)\right\|={\left\|S_{T}\left(t\right)\right\|}_{T},$$ (62)

thus establishing that, for phasor harmonic frequencies *f* equal to a multiple of 1/*T*,

$$\begin{array}{ll} \underset{C}{z}\left[S_{T}\right]\left(\;f\right) & ≜\frac{\overset{T}{\underset{0}{\int }}dt\;S_{T}\left(t\right)e^{i2\pi ft}}{\overset{T}{\underset{0}{\int }}dt\;S_{T}\left(t\right)}\\ & =\frac{{\left\|S_{T}\left(t\right)e^{i2\pi ft}\right\|}_{T}}{{\left\|S_{T}\left(t\right)\right\|}_{T}}=\frac{\left\|S\left(t\right)e^{i2\pi ft}\right\|}{\left\|S\left(t\right)\right\|}=z\left[S\right]\left(\;f\right). \end{array}$$ (63)

While the two definitions Eqs. (52) and (63) give identical results, it is again important to notice that one definition involves a non-periodic function [$S\left(t\right)$], while the other involves its *T*-periodic summation [$S_{T}\left(t\right)$].

The definition of the phasor of $S_{T}\left(t\right)$ [Eq. (63)], which we will call the *cyclic phasor* $\underset{C}{z}\left[S_{T}\right]\left(\;f\right)$ (note the symbol “C” underneath the “z”) because it involves a single period of the recorded periodic signal, connects it to the formalism of *Fourier series*, as discussed next. To simplify notations, we will omit the symbol “C” below the phasor notation “z” in the remainder of the discussion as it should be obvious what definition is used based on the periodicity (or not) of the function involved.

The Fourier series of a *T*-periodic signal $S_{T}\left(t\right)$ is defined as

$$S_{T}\left(t\right)=\frac{1}{2}a_{0}+\overset{+\infty }{\underset{n=1}{\sum }}a_{n}\cos2\pi f_{n}t+\overset{+\infty }{\underset{n=1}{\sum }}b_{n}\sin2\pi f_{n}t,$$ (64)

where the Fourier coefficients $\left(a_{n},b_{n}\right)$ and harmonic frequencies *f*_*n*_ are given by

$$\left\{\begin{array}{c} a_{n}=\frac{2}{T}\overset{T}{\underset{0}{\int }}dt\;S_{T}\left(t\right)\cos\left(2\pi f_{n}t\right),\\ b_{n}=\frac{2}{T}\overset{T}{\underset{0}{\int }}dt\;S_{T}\left(t\right)\sin\left(2\pi f_{n}t\right),\\ f_{n}=\frac{n}{T},\;n\in N. \end{array}\right.$$ (65)

*n* (a positive integer) is the order of the Fourier harmonic. Note that contrary to the Fourier transform, defined for any frequency *f*, Fourier series only involve multiples of the fundamental frequency *f*_0_ = 1/*T*.

With these definitions, the cyclic phasor [Eq. (63)] can be rewritten as

$$z\left[S_{T}\right]\left(\;f_{n}\right)=\frac{a_{n}+ib_{n}}{a_{0}}.$$ (66)

If need be, with the proper normalization of $S_{T}\left(t\right)$, we can obtain *a*_0_ = 1, further simplifying the relation between cyclic phasor and Fourier series component.

*Notations*: in the remainder of this article, we will omit the mention of the phasor harmonic *f* = *f*_*n*_ in the phasor notation when there is no ambiguity and write instead

$$z\left[S_{T}\right]\left(\;f\right)\equiv z\left[S_{T}\right].$$ (67)

The choice of the actual harmonic (or harmonics) to use in phasor analysis will not be discussed here as it is to some extent irrelevant to the topics addressed in this article. For some examples of considerations involving harmonic(s) choices, see, for instance, Refs. 18 and 31.

#### 3. Continuous vs discrete and ungated vs gated phasor

All definitions so far, including Eq. (65), have assumed that the signal $S_{T}\left(t\right)$ was recorded for all values *t* in [0, *T*], which is an idealization. In practice, a signal is recorded experimentally only at a finite number of *t* values, in which case the integrations in Eq. (63) need to be replaced by summations. We will therefore distinguish in the following between “continuous” and “discrete” phasor definitions.

Moreover, most experimental data are effectively binned or time-gated. In other words, while data are tagged with precise time stamps ${\left\{t_{p}\right\}}_{1\leq p\leq N}$ measured with respect to the previous laser pulse, the recorded signal $S_{T}\left(t_{p}\right)$ corresponds effectively to the signal integrated over a period of time $\left[t_{p},t_{p}+W\right]$, where *W* is the gate width (or bin duration). We will therefore also distinguish between “ungated” (or instantaneous) and “gated” (or “binned”) decay definitions and, by extension, speak of phasors of such experimental decays as “continuous phasors” or “discrete phasors.”

The differences between continuous (Sec. III B) and discrete phasors (Sec. III C) of several classes of decays whose phasors can be easily computed analytically will be reviewed: (i) periodic single-exponential decays (PSEDs), (ii) PSEDs with single-exponential IRF, (iii) square-gated PSEDs, and (iv) square-gated PSED with single-exponential IRF. Because they are useful in this type of calculations, the properties of phasors of convolution products will be examined in both cases (continuous and discrete phasors).

### B. Phasor of continuous decays: Continuous phasor

#### 1. Continuous phasor of convolution products

As discussed in Sec. II A, a recorded periodic decay $S_{T}\left(t\right)$ can generally be expressed as a cyclic convolution product [Eq. (14)],

$$S_{T}\left(t\right)=I_{T}\underset{T}{*}F_{0,T}\left(t\right),$$ (68)

where $I_{T}\left(t\right)$ is the *T*-periodic *instrument response function* and $F_{0,T}\left(t\right)$ is the *T*-periodic summation of the sample’s response $F_{0}\left(t\right)$ to a Dirac excitation. $F_{0,T}\left(t\right)$ is easily computed if the analytical form of $F_{0}\left(t\right)$ is known, while $I_{T}\left(t\right)$ is in principle measurable experimentally. However, the importance of Eq. (68) comes from the following property of the continuous cyclic phasor established in Appendix C [“*continuous phasor convolution rule*,” Eq. (C12)]:

$$z\left[f_{T}\underset{T}{*}g_{T}\right]=z\left[f_{T}\right]z\left[g_{T}\right],$$ (69)

where *f*_*T*_ and *g*_*T*_ are two *T*-periodic functions. This property simplifies the computation of the continuous phasor of experimental decays obtained as the cyclic convolution product of two or more functions, as encountered in Sec. II [Eqs. (10), (14), and (38)].

#### 2. Continuous phasor of ungated PSEDs

We will first review a few useful examples of phasors of ungated decays before presenting their counterpart in the presence of a square gate.

##### a. Ungated PSEDs with Dirac IRF.

It is straightforward to verify that for the special case of an ungated PSED with lifetime *τ* [$S_{T}\left(t\right)=\Lambda_{\tau ,T}\left(t\right)$ defined by Eq. (17)], Eq. (63) reads

$$z\left[\Lambda_{\tau ,T}\right]=\frac{1}{1-i2\pi f\tau }=\frac{1+i2\pi f\tau }{1+{\left(2\pi f\tau \right)}^{2}}≜\zeta_{f}\left(\tau \right).$$ (70)

This expression, which we will refer to as the *canonical phasor* of a PSED with lifetime *τ*, $\zeta_{f}\left(\tau \right)$, is of course identical to that of the phasor of infinite, non-periodic single-exponential decays generally encountered in the literature, with the following definitions of the phasor *components* (*g*, *s*) and phasor *modulus m* and *phase φ*:

$$\left\{\begin{array}{c} \zeta_{f}\left(\tau \right)=g\left(\tau \right)+is\left(\tau \right)=m\left(\tau \right)e^{i\varphi \left(\tau \right)},\\ g\left(\tau \right)=\frac{1}{1+{\left(2\pi f\tau \right)}^{2}},\;\\ s\left(\tau \right)=\frac{2\pi f\tau }{1+{\left(2\pi f\tau \right)}^{2}},\\ m\left(\tau \right)=\frac{1}{\sqrt{1+{\left(2\pi f\tau \right)}^{2}}},\;\\ \tan\varphi \left(\tau \right)=2\pi f\tau . \end{array}\right.$$ (71)

The locus (*g*, *s*) of the phasors $\zeta_{f}\left(\tau \right)=g\left(\tau \right)+is\left(\tau \right),\;\tau \geq 0$ of continuous PSEDs is the so-called *universal semicircle* (sometimes called *universal circle*), noted $L_{\infty }$ in the following, defined by

$${\left(g-\frac{1}{2}\right)}^{2}+s^{2}=\frac{1}{4},\;g,s\geq 0.$$ (72)

In particular, $\zeta_{f}\left(0\right)=1$ and $\zeta_{f}\left(\infty \right)=0$.

##### b. Ungated PSEDs with single-exponential IRF.

When the IRF is not a Dirac function, but a single-exponential with time constant *τ*_×_, the phasor of the corresponding *T*-periodic signal, $\Psi_{\tau ,\tau_{\times },T}\left(t\right)=\Lambda_{\tau_{\times },T}\underset{T}{*}\Lambda_{\tau ,T}\left(t\right)$, the cyclic convolution of two PSEDs, is given by [Eq. (D14)]

$$z\left[\Psi_{\tau ,\tau_{\times },T}\right]=\zeta_{f}\left(\tau_{\times }\right)\zeta_{f}\left(\tau \right),$$ (73)

which describes a semicircle rotated by an angle *φ*_×_ and dilated by a factor *m*_×_ given by

$$\left\{\begin{array}{c} \;\varphi_{\times }=\tan^{-1}\left(2\pi f\tau_{\times }\right),\;\\ \;m_{\times }={\left(1+{\left(2\pi f\tau_{\times }\right)}^{2}\right)}^{-\frac{1}{2}}. \end{array}\right.$$ (74)

#### 3. Continuous phasor of square-gated PSEDs

##### a. Square-gated PSEDs with Dirac IRF.

Using Eq. (47) for the corresponding recorded decay $S_{W}\left(t\right)=\Lambda_{\tau ,T,W}\left(t\right)$ and reporting it in Eq. (63), or alternatively, using the fact that $\Lambda_{\tau ,T,W}\left(t\right)={\bar{\Pi }}_{W,nT}\underset{T}{*}\Lambda_{\tau ,T}\left(t\right)$ is the cyclic convolution of a PSED and a mirrored square gate, we obtain

$$\left\{\begin{array}{c} z\left[\Lambda_{\tau ,T,W}\right]=z\left[{\bar{\Pi }}_{W,nT}\right]\zeta_{f}\left(\tau \right)=M_{W}e^{-i\varphi_{W}}\zeta_{f}\left(\tau \right)≜z_{\left[W\right]}\left[\Lambda_{\tau ,T}\right],\\ \varphi_{W}=\pi fW,\\ M_{W}=\frac{\sin\varphi_{W}}{\varphi_{W}}, \end{array}\right.$$ (75)

where the expression of the canonical phasor $\zeta_{f}\left(\tau \right)$ is given by Eq. (70) and the phasor of a mirrored square gate is derived in Appendix C.5.1 [Eq. (C30)]. Note the subscript “[W]” (*W* within square brackets) in the phasor notation, which indicates a square gate of width *W*.

**FIG. 3.**
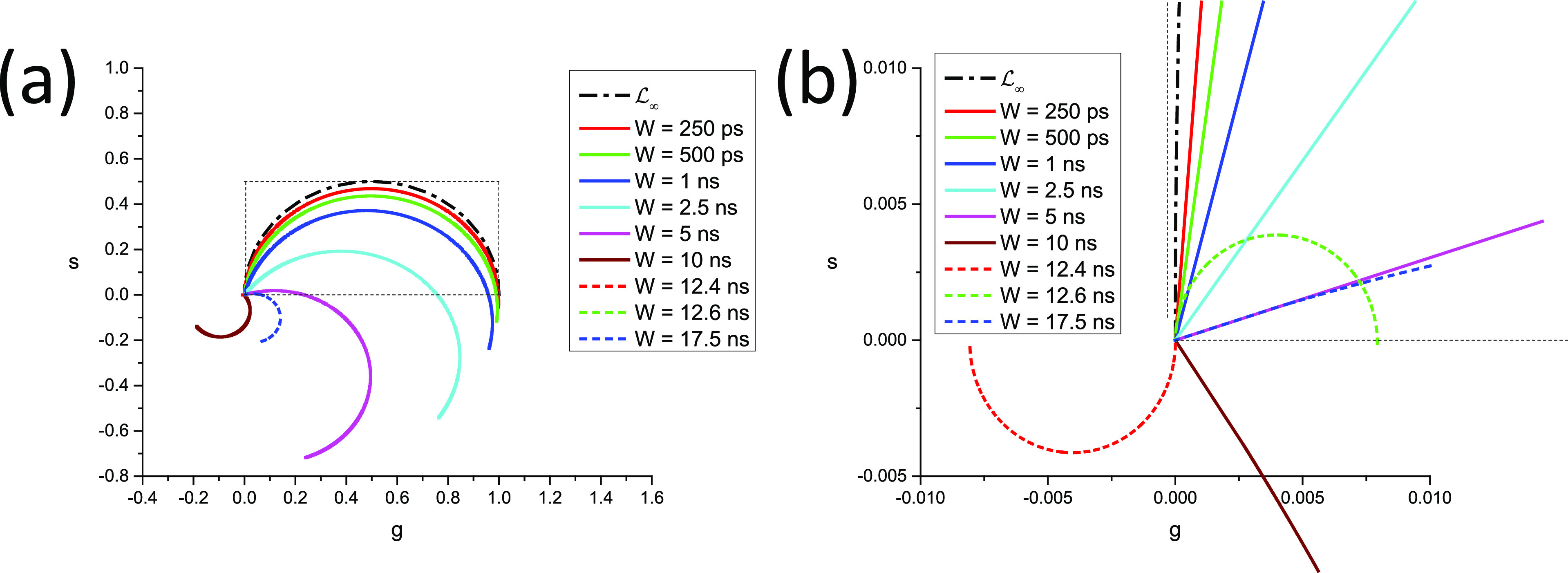
Locus of continuous phasors of square-gated periodic single-exponential decays for different values of the gate width W. *T* = 12.5 ns, f_1_ = 80 MHz: (a) overview and (b) detail of the (0,0) region.

In other words, as illustrated in Fig. 3, the continuous phasor of a square-gated PSED is identical to that of the corresponding ungated PSED, up to a rotation by an angle −*φ*_*W*_ = −*πfW* about the origin and a dilation by a factor *M*_*W*_ given in Eq. (75), both of which are independent of *τ*. The locus of continuous phasors of square-gated PSEDs is thus a rotated, dilated semicircle, which we will refer to as the *SEPL for square-gated decays* and denote by $L_{\left[W\right]}$. Its equation is given by

$$g^{2}+s^{2}=M_{W}\left(\cos\varphi_{W}g-\sin\varphi_{W}s\right),$$ (76)

which describes a circle whose center $\left(g_{c},s_{c}\right)$ and radius *r* are given by

$$\left\{\begin{array}{c} g_{C}=\frac{M_{W}}{2}\cos\varphi_{W},\\ s_{C}=-\frac{M_{W}}{2}\sin\varphi_{W},\\ r=\frac{M_{W}}{2}. \end{array}\right.$$ (77)

The radius of this circle decreases inversely to *W* and, because of the sine function in the expression for *M*_*W*_ [Eq. (75)], can occasionally be equal to zero. However, this only happens in trivial cases when the gate period is a multiple of the laser period, in which case the square-gated decay is a constant, resulting in a constant phasor, no matter what lifetime is considered.

In all cases, $z_{\left[W\right]}\left[\Lambda_{0,T}\right]=M_{W}e^{i\varphi_{W}}$ and $z_{\left[W\right]}\left[\Lambda_{\infty ,T}\right]=0$.

##### b. Square-gated PSEDs with single-exponential IRF.

The continuous phasor of a square-gated PSED convolved with a single-exponential excitation or IRF with time constant *τ*_×_, $\Psi_{\tau ,\tau_{\times },T,W}\left(t\right)={\bar{\Pi }}_{W,nT}\underset{T}{*}\Lambda_{\tau_{\times },T}\underset{T}{*}\Lambda_{\tau ,T}\left(t\right)$, is given by the product of three phasors [Eq. (D24)],

$$\begin{array}{ll} z\left[\Psi_{\tau ,\tau_{\times },T,W}\right] & =M_{W}e^{-i\varphi_{W}}\zeta_{f}\left(\tau_{\times }\right)\zeta_{f}\left(\tau \right)\\ & =z_{\left[W\right]}\left[\Lambda_{\tau_{\times },T}\right]\zeta_{f}\left(\tau \right)≜z_{\left[W\right]}\left[\Psi_{\tau ,\tau_{\times },T}\right]. \end{array}$$ (78)

The locus of these phasors is thus a semicircle rotated by an angle $\varphi_{*}-\varphi_{W}=\tan^{-1}\left(2\pi f\tau_{*}\right)-\pi fW$ and dilated by a factor m_*_M_*W*_ [given in Eqs. (74) and (75)].

#### 4. Continuous phasor of arbitrary periodic decays

##### a. Dirac IRF.

By analogy with the discussion of Sec. II A 7, any *T*-periodic function $F_{0,T}\left(t\right)$ can be expressed in terms of a *T*-summation of a non-periodic function $F_{0}\left(t\right)$ [Eq. (11)], which, in turn, can be expressed in terms of a $\phi_{0}\left(\tau \right)$-weighted integral of normalized exponential functions $\Lambda_{\tau }\left(t\right)$ as

$$F_{0}\left(t\right)=\overset{\infty }{\underset{0}{\int }}d\tau \;\phi_{0}\left(\tau \right)\Lambda_{\tau }\left(t\right).$$ (79)

It follows that $F_{0,T}\left(t\right)$ can be rewritten as

$$F_{0,T}\left(t\right)=\overset{\infty }{\underset{0}{\int }}d\tau \;\phi_{0}\left(\tau \right)\Lambda_{\tau ,T}\left(t\right).$$ (80)

The integral of $F_{0,T}\left(t\right)$ over [0, *T*] is given by

$${\left\|F_{0,T}\left(t\right)\right\|}_{T}=\overset{T}{\underset{0}{\int }}dt\;F_{0,T}\left(t\right)=\overset{\infty }{\underset{0}{\int }}d\tau \;\phi_{0}\left(\tau \right)=\overset{\infty }{\underset{0}{\int }}dt\;F_{0}\left(t\right)=\left\|F_{0}\left(t\right)\right\|.$$ (81)

Inserting Eqs. (80) and (81) in Eq. (63) yields

$$z\left[F_{0,T}\right]=\frac{\overset{\infty }{\underset{0}{\int }}d\tau \;\phi_{0}\left(\tau \right)\zeta_{f}\left(\tau \right)}{{\left\|F_{0,T}\left(t\right)\right\|}_{T}}=\frac{\overset{\infty }{\underset{0}{\int }}d\tau \;\phi_{0}\left(\tau \right)\zeta_{f}\left(\tau \right)}{\left\|F_{0}\left(t\right)\right\|}=z\left[F_{0}\right].$$ (82)

Due to the invariance of the phasor by dilation [Eq. (54)], this can also be written in terms of the ${\left\|\right\|}_{T}$-normalized decay $f_{0,T}\left(t\right)$ and ${\left\|\right\|}_{T}$-normalized weight function $\mu_{0}\left(\tau \right)$,

$$\left\{\begin{array}{c} f_{0,T}\left(t\right)=\frac{F_{0,T}\left(t\right)}{{\left\|F_{0,T}\left(t\right)\right\|}_{T}},\\ \mu_{0}\left(\tau \right)=\frac{\phi_{0}\left(\tau \right)}{{\left\|F_{0,T}\right\|}_{T}}, \end{array}\right.$$ (83)

as

$$\left\{\begin{array}{c} f_{0,T}\left(t\right)=\overset{\infty }{\underset{0}{\int }}d\tau \;\mu_{0}\left(\tau \right)\Lambda_{\tau ,T}\left(t\right),\\ z\left[f_{0,T}\right]=\overset{\infty }{\underset{0}{\int }}d\tau \;\mu_{0}\left(\tau \right)\zeta_{f}\left(\tau \right). \end{array}\right.$$ (84)

Equation (84) expresses the fact that the phasor of an arbitrary periodic function, expressed as a normalized weighted sum of PSEDs $\Lambda_{\tau ,T}\left(t\right)$, is expressed as the same weighted sum but of the canonical phasors $\zeta_{f}\left(\tau \right)$. This formula provides a formal extension to arbitrary periodic functions of the formalism discussed in this article, our discussion being mostly focused on PSEDs for simplicity.

A useful particular case of Eq. (79) is encountered when the sample’s emission can be written as a *sum of exponentials*,

$$\left\{\begin{array}{c} F_{0}\left(t\right)=\overset{n}{\underset{i=1}{\sum }}a_{i}e^{-t/\tau_{i}}=\overset{n}{\underset{i=1}{\sum }}a_{i}\tau_{i}\Lambda_{\tau_{i}}\left(t\right)=\overset{\infty }{\underset{0}{\int }}d\tau \;\phi_{0}\left(\tau \right)\Lambda_{\tau }\left(t\right),\\ \phi_{0}\left(\tau \right)=\overset{n}{\underset{i=1}{\sum }}a_{i}\tau_{i}\delta \left(\tau -\tau_{i}\right). \end{array}\right.$$ (85)

From this definition of $\phi_{0}\left(\tau \right)$, we obtain

$$\left\{\begin{array}{c} \left\|F_{0}\left(t\right)\right\|=\overset{n}{\underset{i=1}{\sum }}a_{i}\tau_{i}={\left\|F_{0,T}\left(t\right)\right\|}_{T},\\ \mu_{0}\left(t\right)=\overset{n}{\underset{i=1}{\sum }}\mu_{i}\delta \left(\tau -\tau_{i}\right),\;\mu_{i}=\frac{a_{i}\tau_{i}}{\overset{n}{\underset{j=1}{\sum }}a_{j}\tau_{j}}. \end{array}\right.$$ (86)

Equation (84) thus reads

$$\left\{\begin{array}{c} f_{0,T}\left(t\right)=\overset{n}{\underset{i=1}{\sum }}\mu_{i}\Lambda_{\tau_{i},T}\left(t\right),\\ z\left[f_{0,T}\right]=\overset{n}{\underset{i=1}{\sum }}\mu_{i}\zeta_{f}\left(\tau_{i}\right), \end{array}\right.$$ (87)

which expresses the fact that the phasor of a normalized weighted sum of normalized PSEDs $\Lambda_{\tau_{i},T}\left(t\right)$ can be expressed with the same weighted sum of their individual phasors $\zeta_{f}\left(\tau_{i}\right)$.

##### b. Arbitrary IRF.

The case of arbitrary IRFs will be discussed in the context of phasor calibration in Sec. VIII B.

### C. Phasor of decays with discrete sampling: “Discrete” phasor

#### 1. Definitions

If a signal is only recorded at a finite number *N* of temporal locations${\left\{t_{p}\right\}}_{1\leq p\leq N}$, separated by intervals${\left\{\theta_{p}\right\}}_{1\leq p\leq N},\;\theta_{p}=t_{p+1}-t_{p}$, a discrete version of the cyclic phasor definition [Eq. (63)] needs to be used,

$$\left\{\begin{array}{c} z_{N}\left[S_{T}\right]\left(\;f\right)≜{\left\|S_{T}\left(t_{p}\right)e^{i2\pi ft_{p}}\right\|}_{N}/{\left\|S_{T}\left(t_{p}\right)\right\|}_{N},\\ {\left\|S_{T}\left(t_{p}\right)\right\|}_{N}≜\overset{N}{\underset{p=1}{\sum }}\theta_{p}S_{T}\left(t_{p}\right),\\ {\left\|S_{T}\left(t_{p}\right)e^{i2\pi ft_{p}}\right\|}_{N}≜\overset{N}{\underset{p=1}{\sum }}\theta_{p}S_{T}\left(t_{p}\right)e^{i2\pi ft_{p}}. \end{array}\right.$$ (88)

In the definition of the last value, *θ*_*N*_, the periodicity of the decay is used: *θ*_*N*_ = *t*_1_ + *T* − *t*_*N*−1_. From this definition, it is obvious that the discrete phasor, like the continuous phasor, is invariant by dilation,

$$\left(\forall a\right),\;z_{N}\left[aS_{T}\right]=z_{N}\left[S_{T}\right].$$ (89)

Definition (88) is *not* assuming that the *N* intervals cover the whole laser period. In other words, the record “duration” *D*, defined by

$$D=\overset{N}{\underset{p=1}{\sum }}\theta_{p},$$ (90)

might well be different from the decay period *T*, although *D* = *T* is often the case experimentally. We will indicate when this assumption is used and dedicate a specific section to the cases where *D* < *T* (Sec. V, the effect of decay truncation). As before, we will drop the mention of the phasor harmonic frequency when it is redundant and write

$$z_{N}\left[S_{T}\right]\left(\;f\right)\equiv z_{N}\left[S_{T}\right]$$ (91)

and use the shorthand notation ${\left\|S_{T}\right\|}_{N}\equiv {\left\|S_{T}\left(t_{p}\right)\right\|}_{N}$ when this does not create any ambiguity.

In typical cases where the recording locations are equidistant (*θ*_*p*_ = *θ*, 1 ≤ *p* ≤ *N*; *D* = *Nθ*),

$${\left\|S_{T}\left(t_{p}\right)\right\|}_{N}=\theta \overset{N}{\underset{p=1}{\sum }}S_{T}\left(t_{p}\right)=\frac{D}{N}\overset{N}{\underset{p=1}{\sum }}S_{T}\left(t_{p}\right).$$ (92)

We will assume this condition to be met in the subsequent discussion.

From Eq. (92), it follows that

$$\underset{N\to \infty }{\lim}{\left\|S_{T}\left(t_{p}\right)\right\|}_{N}=\overset{T}{\underset{0}{\int }}dt\;S_{T}\left(t\right)={\left\|S_{T}\left(t\right)\right\|}_{T}.$$ (93)

Some authors use a slightly different definition, where the argument of the complex exponential term in Eqs. (88) and (92) is replaced by $2\pi f\left(t_{p}+W/2\right)$, i.e., the gate’s center *t*_*p*_ + *W*/2 is used instead of the gate beginning *t*_*p*_ in the complex exponential argument.^14^ While this choice is legitimate, it breaks the direct connection to the discrete Fourier transform [Eq. (66)]. As we shall see, its only effect is to multiply the phasor as calculated in Eq. (92) by a constant term *e*^*iπfW*^, i.e., it rotates the phasor by an angle *πfW*. This may have the undesirable effect to move the phasor of *τ* = 0 away from its standard location $z\left[\Lambda_{0,T}\right]=1$.

We will now examine some simple situations where the phasor of PSEDs can be expressed in compact form, as done for continuous phasors in Sec. III B.

#### 2. Discrete phasor of convolution products

When *D* = *T*, the discrete phasor as defined by Eq. (92) is related to the *discrete Fourier transform* (DFT) of the sequence of equidistant data points $\left\{S_{T}\left(t_{k}\right)=S_{k}\right\},1\leq k\leq N$,

$$DF\left[S_{T}\right]\left(n\right)=\overset{N}{\underset{k=1}{\sum }}S_{k}e^{-i2\pi n\frac{k-1}{N}},\;0\leq n\leq N-1,$$ (94)

as can easily be seen from the following identities:

$$\left\{\begin{array}{c} \frac{k-1}{N}=\frac{\left(k-1\right)\theta }{N\theta }=\frac{t_{k}}{T},\\ f_{n}=\frac{n}{T}. \end{array}\right.$$ (95)

Therefore,

$$z_{N}\left[S_{T}\right]\left(f_{n}\right)=\frac{D{F\;}^{*}\left[S_{T}\right]\left(n\right)}{DF^{*}\left[S_{T}\right]\left(0\right)},$$ (96)

defined for all possible values of the phasor harmonic frequency *f*_*n*_ = *n*/*T*, 0 ≤ *n* ≤ *N* − 1. Note that this equivalence relies on a definition of the phasor harmonic frequency *f*_*n*_ as a multiple of the inverse of the signal period *T* and of the sampling times as $t_{k}=\left(k-1\right)\theta ,\;1\leq k\leq N$ [Eq. (95)]. As discussed in Appendix C.4, this connection to the DFT is not particularly useful because convolution products involved in discrete phasor calculations are continuous convolutions, not discrete convolutions.

In fact, a *negative* “discrete phasor convolution product rule” applies [Eq. (C17)],

$$z_{N}\left[F_{T}\underset{T}{*}G_{T}\right]\neq z_{N}\left[F_{T}\right]z_{N}\left[G_{T}\right],$$ (97)

which states that, in general, knowing the discrete phasors $z_{N}\left[F_{T}\right]$ and $z_{N}\left[G_{T}\right]$ of the components of a convolution product $F_{T}\underset{T}{*}G_{T}$ does not help with calculating the phasor $z_{N}\left[F_{T}\underset{T}{*}G_{T}\right]$ of the continuous convolution product. This has profound implications when dealing with discrete phasor calibration, as discussed in Sec. VIII.

In some particular cases, a *weak version* of the discrete phasor convolution rule applies [Eq. (C21)],

$$z_{N}\left[I_{T}\underset{T}{*}F_{T,\lambda }\right]=\kappa \;z_{N}\left[I_{T}\right]z_{N}\left[F_{T,\lambda }\right],$$ (98)

where *κ* is constant for a family of decays $\left\{F_{T,\lambda }\left(t\right)\right\},\;\lambda \in \Omega$, where Ω is a subset of R and $I_{T}\left(t\right)$ represents the *T*-periodic instrument response function. This “*weak discrete phasor convolution rule*” allows a limited use of the standard phasor calibration approach for this specific family of decays, as will be discussed in Sec. VIII.

#### 3. Discrete phasor of ungated PSEDs

##### a. Ungated PSEDs with Dirac IRF.

For an ungated PSED, and assuming *θ* = *T*/*N* (i.e., gates covering the whole laser period, *D* = *T*) and *f* = *n*/*T*, we obtain [Appendix B, Eq. (B3)]

$$\left\{\begin{array}{c} \zeta_{f,N}\left(\tau \right)≜z_{N}\left[\Lambda_{\tau ,T}\right]=\frac{1-x}{1-xe^{i\alpha }},\\ x\left(\tau \right)=e^{-\theta /\tau },\alpha =2\pi f\theta . \end{array}\right.$$ (99)

We will refer to this function as the *canonical discrete phasor* of *T*-PSEDs, $\zeta_{f,N}\left(\tau \right)$.

In this case, the locus of phasors of discrete ungated PSEDs is a *circular arc* (see Fig. 3), whose properties are discussed in Appendix B. In particular, its equation, center, and radius are given by [Eq. (B9)]

$$\left\{\begin{array}{c} {\left(g-g_{c}\right)}^{2}+{\left(s-s_{c}\right)}^{2}=r^{2},\\ g_{c}=\frac{1}{2},\\ s_{c}=-\frac{1}{2}\tan\left(\alpha /2\right),\\ r=\frac{1}{2\left|\cos\left(\alpha /2\right)\right|}. \end{array}\right.$$ (100)

**FIG. 4.**
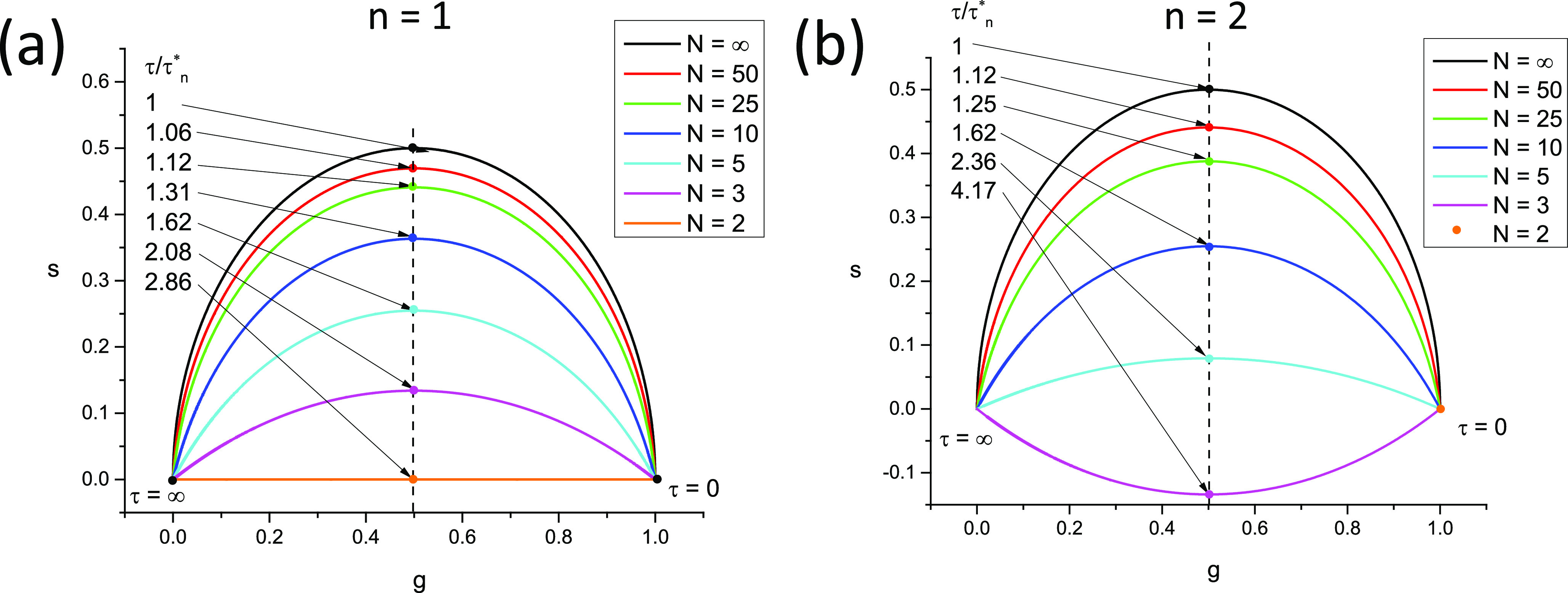
Locus of discrete phasors of single-exponential decays for different choices of *N*, the number of sampling points, and *n*, the phasor harmonic. [(a) *n* = 1, (b) *n* = 2] The extremum of these curves (indicated by a dot of the same color) is located at *g* = ½ and is attained for different values of *τ* expressed in units of τ^*^_n_ = *T*/2*πn*. Note that for *n* = 1, *N* = 2 results in a straight line, while for *n* = 2, *N* = 2 results in all phasors being located at 1. An example of a situation where *s* < 0 is provided in (b) (*n* = 2, *N* = 3).

The two extreme values, $z_{N}\left[\Lambda_{0,T}\right]=1$ and $z_{N}\left[\Lambda_{\infty ,T}\right]=0$, remain identical to those of the continuous case. We will refer to this curve as the *SEPL for discrete phasors of ungated PSEDs* and denote it $L_{N}$, the subscript “*N*” indicating the discrete nature of the decays (and the number of gates used to cover the whole laser period). Note that $L_{N}$ also depends on the chosen harmonic *n* via the harmonic frequency *f* [compare Figs. 4(a) and 4(b)].

For large values of *N*, Eq. (99) tends to Eq. (70), as expected, and $L_{N}$ tends to $L_{\infty }$, the standard universal semicircle.

##### b. Ungated PSEDs with single-exponential IRF.

Using the definition of $\Psi_{\tau ,\tau_{\times },T}\left(t\right)$ [Eq. (20)] in Eq. (92) yields [Appendix D, Eq. (D31)]

$$\left\{\begin{array}{c} z_{N}\left[\Psi_{\tau ,\tau_{\times },T}\right]=\frac{1-x}{1-xe^{i\alpha }}\frac{1-x_{\times }}{1-x_{\times }e^{i\alpha }}e^{i\alpha }=e^{i\alpha }\zeta_{f,N}\left(\tau \right)\zeta_{f,N}\left(\tau_{\times }\right),\\ x\left(\tau \right)=e^{-\theta /\tau },\;x_{\times }=x\left(\tau_{\times }\right)=e^{-\theta /\tau_{\times }},\;\alpha =2\pi f\theta . \end{array}\right.$$ (101)

This can be rewritten as

$$z_{N}\left[\Psi_{\tau ,\tau_{\times },T}\right]=e^{i\alpha }z_{N}\left[\Lambda_{\tau_{\times },T}\right]z_{N}\left[\Lambda_{\tau ,T}\right],$$ (102)

which shows that the discrete phasor of single-exponential decays convolved with a *T*-periodic single-exponential IRF is a rotated and dilated version of the discrete phasor of PSEDs. We will return to this identity in Sec. VIII, when discussing calibration. Note that Eq. (102) is an example of the weak discrete phasor convolution rule mentioned in Sec. III C 2 since $\Psi_{\tau ,\tau_{\times },T}\left(t\right)=\Lambda_{\tau_{\times },T}\underset{T}{*}\Lambda_{\tau ,T}\left(t\right)$ is a convolution product and the constant *κ* = *e*^*i*2*πfθ*^ does not depend on the lifetime *τ*.

#### 4. Discrete phasor of square-gated PSEDs

##### a. Square-gated PSEDs with Dirac IRF.

To obtain the phasor of a square-gated PSED recorded at discrete points, Eq. (92) is used with $S_{T}\left(t\right)$ given by $\Lambda_{\tau ,T,W}\left(t\right)$ in Eq. (47). The result is [Appendix B, Eq. (B37)]

$$z_{N\left[W\right]}\left[\Lambda_{\tau ,T}\right]≜z_{N}\left[\Lambda_{\tau ,T,W}\right]=\frac{-\frac{1-e^{ir\alpha }}{1-e^{i\alpha }}+\frac{1-uy^{-1}x^{r}e^{ir\alpha }}{1-xe^{i\alpha }}}{\left(k+1\right)N-r+\frac{1-uy^{-1}x^{r}}{1-x}},$$ (103)

where the following intermediate variables have been introduced:

$$\left\{\begin{array}{c} k=\left\lfloor W/T\right\rfloor ,\;\\ \omega =W\left[T\right]=W-kT,\;\\ r=\left\lceil \frac{T-\omega }{\theta }\right\rceil ,\;\\ x\left(\tau \right)=e^{-\theta /\tau };\;y\left(\tau \right)=e^{-T/\tau },\;u\left(\tau \right)=e^{-\omega /\tau },\;\alpha =2\pi f\theta ,\; \end{array}\right.$$ (104)

and the notation $\left\lceil x\right\rceil$ denotes the “upper” integer part of *x* (or ceil—or ceiling—function in most programming languages),

$$\left.\left.\forall x\in R,\;x\in \right]n-1,n\right],n\in Z\Rightarrow \;\left\lceil x\right\rceil =n.$$ (105)

As before, the gate duration *W* can take any positive value (including values larger than the laser period *T*, in which case, each gate in a frame will be separated by more than one laser period from the next); *r* is an index value used to determine which expression of $\Lambda_{\tau ,T,W}\left(t\right)$ to use in Eq. (47); *x*, *y*, *u*, and α are introduced to simplify notations.

The locus of the discrete phasors of PSEDs is, in general, a complex curve, which cannot be reduced to an algebraic equation due to the presence of the term *x*^*r*^ in Eq. (103). We will refer to this curve as the *SEPL for discrete phasors of square-gated PSEDs* and denote it as $L_{N\left[W\right]}$, subscript “*N* [*W*]” indicating that both discrete decays (*N* gates) and a square gate of duration *W* are considered. Equation (103) introduces a similar notation, $z_{N\left[W\right]}\left[S_{T}\right]$, for the discrete phasor of a square-gated periodic decay, *S*_*T*_.

In special cases where *T* − *ω* is proportional to the gate step *θ* (which, since *T* = *Nθ* is assumed, is equivalent to the gate width *W* being proportional to *θ*), one obtains the following identity [Appendix B, Eq. (B40)]:

$$\begin{array}{c} W=q\theta \;\Rightarrow \;z_{N\left[W\right]}\left[\Lambda_{\tau ,T}\right]=\frac{\sin q\frac{\alpha }{2}}{q\sin\frac{\alpha }{2}}\;e^{-i\left(q-1\right)\frac{\alpha }{2}},\\ z_{N}\left[\Lambda_{\tau ,T}\right]=\frac{\sin q\frac{\alpha }{2}}{q\sin\frac{\alpha }{2}}\;e^{-i\left(q-1\right)\frac{\alpha }{2}}\zeta_{f,N}\left(\tau \right). \end{array}$$ (106)

Using the expression for the discrete phasor of the mirror square-gate function derived in Appendix C [Eq. (C37)], this equation can be rewritten as

$$W=q\theta \;\Rightarrow \;z_{N\left[W\right]}\left[\Lambda_{\tau ,T}\right]=e^{i\alpha }\;z_{N}\left[{\bar{\Pi }}_{W,nT}\right]\;z_{N}\left[\Lambda_{\tau ,T}\right].$$ (107)

In other words, it is an *arc of circle* rotated about 0 and with a diameter $d_{N\left[W\right]}$ given by

$$\left\{\begin{array}{c} d_{N\left[W\right]}=\left|z_{N\left[W\right]}\left[\Lambda_{0,T}\right]\right|=\left|\frac{\sin q\frac{\alpha }{2}}{q\sin\frac{\alpha }{2}}\right|,\\ T=N\theta ,\;W=q\theta ,\;\alpha =2\pi f\theta , \end{array}\right.$$ (108)

which decreases as *q* (i.e., *W*) increases.

A particular case of interest is *W* = *θ*, i.e., *q* = 1, which corresponds to adjacent gates (contiguous and non-overlapping gates). We then have

$$W=\theta \;\Rightarrow \;z_{N\left[W\right]}\left[\Lambda_{\tau ,T}\right]=\;z_{N}\left[\Lambda_{\tau ,T}\right],$$ (109)

i.e., the discrete phasor of square-gated PSED with adjacent gates is equal to the discrete phasor of ungated PSED. This situation is that encountered with TCSPC data, where each “bin” of a discrete decay is contiguous to the next one. In this case, the SEPL is therefore an arc of circle ($L_{N}$) that only depends on the number of bins, not on their actual size.

In all other cases, $L_{N\left[W\right]}$ is a complex curve passing through $z_{N\left[W\right]}\left[\Lambda_{\infty ,T}\right]=0$ and $z_{N\left[W\right]}\left[\Lambda_{0,T}\right]=z_{N\left[r\left(W\right)\right]}\left[\Lambda_{0,T}\right]$, where *r*(*W*) is given by

$$r\left(W\right)=\left\lceil \frac{T-W\left[T\right]}{\theta }\right\rceil ,$$ (110)

and the discrete phasor of a square-gated PSED with 0 lifetime is given by [Appendix B, Eqs. (B41) and (B42)]

$$z_{N\left[W\right]}\left[\Lambda_{0,T}\right]=\left\{\begin{array}{c} -\frac{1}{\left(k+1\right)N-r+1}\frac{\sin\left(r-1\right)\frac{\alpha }{2}}{\sin\frac{\alpha }{2}}e^{ir\frac{\alpha }{2}},\;\frac{T-\omega }{\theta }\notin N\\ -\frac{1}{\left(k+1\right)N-r}\frac{\sin r\frac{\alpha }{2}}{\sin\frac{\alpha }{2}}e^{i\left(r+1\right)\frac{\alpha }{2}},\;\frac{T-\omega }{\theta }\in N. \end{array}\right.$$ (111)

**FIG. 5.**
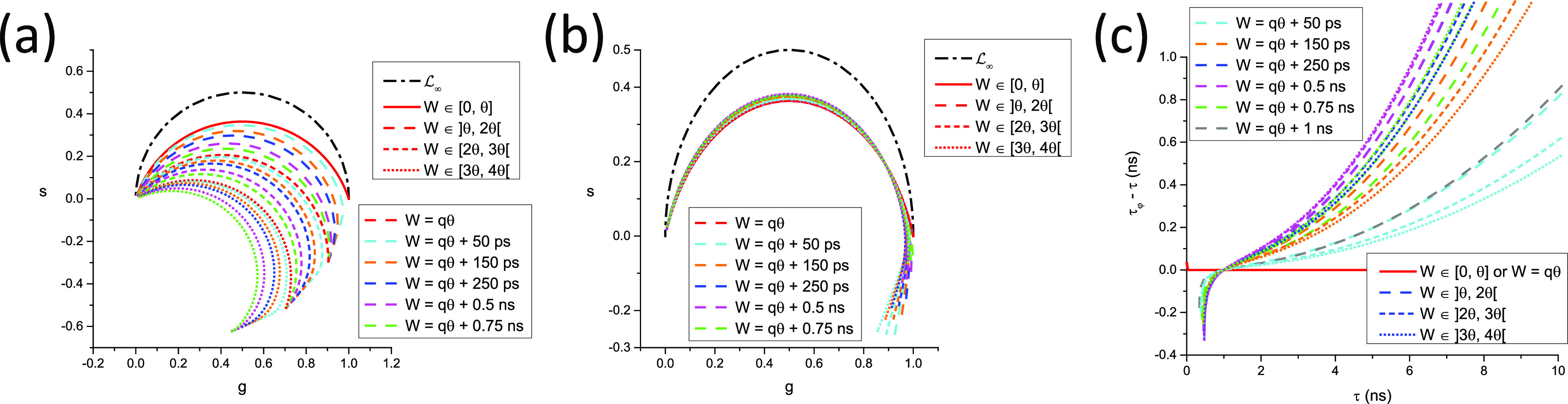
SEPL for discrete square-gated phasors. $L_{N\left[W\right]}$ for a constant number of gates *N* = 10 (and gate step *θ* = *T*/*N*, *T* = 12.5 ns, f = 1/*T*) and varying gate width. (a) Uncalibrated $L_{N\left[W\right]}$ and (b) $L_{N\left[W\right]}$ calibrated using *τ*_*C*_ = 1 ns. (a) For *W* ≤ *θ*, $L_{N\left[W\right]}$ is independent of W and equal to $L_{N}$ (red solid circular arc). For *W* ϵ ]*θ*, 2*θ*[ (long dashed curves), all $L_{N\left[W\right]}$ share a common $z_{N\left[W\right]}\left[\Lambda_{0,T}\right]$ and $z_{N\left[W\right]}\left[\Lambda_{\infty ,T}\right]$, but only $L_{N\left[W\right]}$ for *W* = *θ* (solid red curve) is a circular arc. Similarly, for *W* ϵ [2*θ*, *3θ*[ (short dashed curves) and *W* ϵ [3*θ*, 4*θ*[ (dotted curves), the different $L_{N\left[W\right]}$ share a common $z_{N\left[W\right]}\left[\Lambda_{0,T}\right]$ and $z_{N\left[W\right]}\left[\Lambda_{\infty ,T}\right]$ in each group, but only $L_{N\left[W\right]}$ for *W* = 2*θ* (red short dashed curve) and for *W* = 3*θ* (red dotted curve) are circular arcs. (b) The fact that only one of the $L_{N\left[W\right]}$ within each group is a circular arc is clearly visible after rotation bringing the phasor of *τ* = 1 ns back to $z_{N\left[W\right]}\left[\Lambda_{1,T}\right]$. All $L_{N\left[W\right]}$ for *W* = *kθ* are mapped to $L_{N}$ (circular arc) after calibration, while the others are clearly different from one another. (c) Difference between the pseudo-phase lifetime and the real lifetime computed for the various calibrated $L_{N\left[W\right]}$ curves shown in (b). As expected due to the choice of *τ*_*c*_ = 1 ns as the calibration lifetime, the difference is minimal around *τ* = 1 ns and increases around this value, demonstrating the limitations of phasor calibration in the general case.

In other words, $L_{N\left[W\right]}$ characterized by the same value of *r*(*W*) = *r* [Eq. (110)], i.e., for which $\omega \in [\left(N-r\right)\theta ,\left(N-r+1\right)\theta [$ share the same two points, 0 and $z_{N\left[W\right]}\left[\Lambda_{0,T}\right]$. This property is illustrated in Fig. 5(a) for *N* =10, *T* = 12.5 ns = 1/*f*, where curves characterized by the same *r* value are represented with the same style, while colors indicate different values of *W* within a given interval $[q\theta ,\left(q+1\right)\theta [$. Because the curves at the boundaries of these intervals are different arcs of circle, the intermediate curves progressively “interpolate” between those two regular curves, including curves that are best described qualitatively as a section of circular arc connected to the next circular arc by an almost straight “stem.”

The only exception to this behavior is the series of $L_{N\left[W\right]}$ for *W* ≤ *θ*, where the SEPL is identical to $L_{N}$ as can be easily verified [Appendix B, Eq. (B46) and Fig. 4(a)].

It is easy to verify that in the limit *W* → 0, one recovers the discrete phasor of an ungated signal [Eq. (99)]. Similarly, in the limit *N* → *∞*, one recovers the continuous phasor of a square-gated signal [Eq. (75)].

##### b. Square-gated PSEDs with single-exponential IRF.

The expression for the discrete phasor of a square-gated PSED with single-exponential IRF is derived in Appendix D.9 [Eq. (D47)] and does not correspond to any simple curve, even in the particular case where the gate width *W* is equal to the gate step *θ* (contiguous gates),

$$\left\{\begin{array}{c} z_{N\left[W\right]}\left[\Psi_{\tau ,\tau_{\times },T}\right]≜z_{N}\left[\Psi_{\tau ,\tau_{\times },T,W}\right]=\frac{-\frac{1-e^{ir\alpha }}{1-e^{i\alpha }}+\frac{1}{\tau -\tau_{\times }}\left(\tau \frac{1-\beta e^{ir\alpha }}{1-xe^{i\alpha }}-\tau_{\times }\frac{1-\beta_{\times }e^{ir\alpha }}{1-x_{\times }e^{i\alpha }}\right)}{\left(k+1\right)N-r+\frac{1}{\tau -\tau_{\times }}\left(\tau \frac{1-\beta }{1-x}-\tau_{\times }\frac{1-\beta_{\times }}{1-x_{\times }}\right)},\\ k=\left\lfloor \frac{W}{T}\right\rfloor ,\;r=\left\lceil \frac{T-\omega }{\theta }\right\rceil ,\\ y\left(\tau \right)=e^{-T/\tau },\;y_{\times }\left(\tau \right)=e^{-T/\tau_{\times }},\;u\left(\tau \right)=e^{-\omega /\tau },\;u_{\times }\left(\tau \right)=e^{-\omega /\tau_{\times }},\\ \beta \left(\tau \right)=ux^{r}y^{-1},\;\beta_{\times }\left(\tau \right)=u_{\times }x_{\times }^{r}y_{\times }^{-1}. \end{array}\right.$$ (112)

**FIG. 6.**
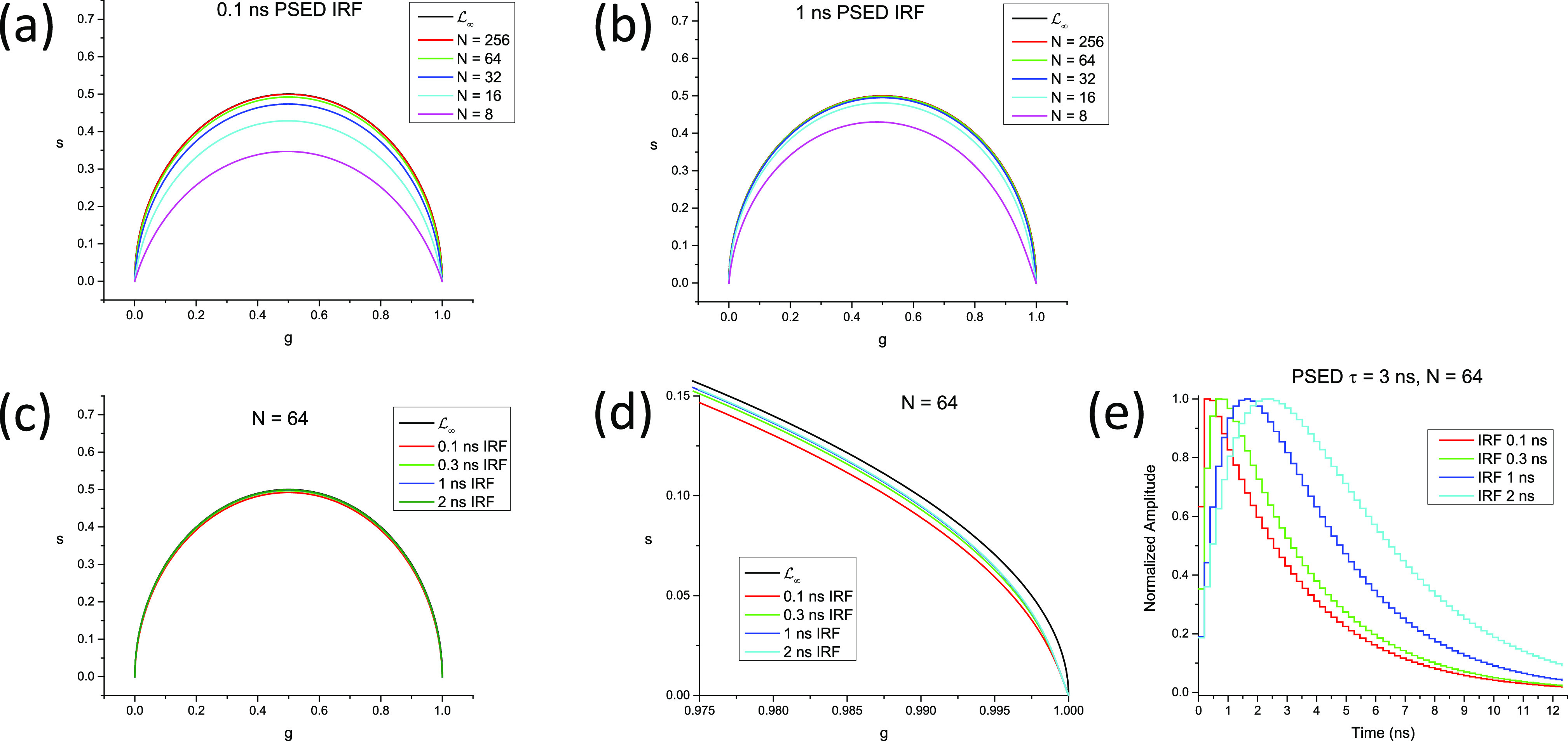
Effect of binning and IRF width on the phasor of TCSPC data. This figure illustrates the discussion of Sec. III C 4 a on the phasor of PSED with a single-exponential IRF, binned with a finite number of bins *N*. A laser period *T* = 12.5 ns was assumed, and an harmonic frequency f = 1/*T* was used. (a) Single-exponential IRF with time constant *τ*_0_ = 0.1 ns. (b) Single-exponential IRF with time constant *τ*_0_ = 1 ns. Note how the wider IRF brings the calibration closer to $L_{\infty }$. [(c) and (d)] Effect of the IRF time constant (*τ*_0_ = 0.1 ns–2 ns) on the SEPL at a fixed bin number (*N* = 64). All curves are very close to $L_{\infty }$ as can be seen in the detail of the (1,0) region shown in (d). (e) Examples of binned *τ* = 3 ns PSED convolved with the single-exponential IRFs used in (a)–(d). Note that all SEPLs have been rotated/rescaled so that the phasor of the 0-lifetime decays are located at the point (1,0) (see Sec. VIII).

This expression can however be used to explore the effect of different gate width/steps on the calibrated SEPL in a non-Dirac excitation case. As will be discussed in Sec. VIII, the calibrated SEPL can in some cases be close to $L_{\infty }$. Figure 6 provides a simple illustration of the non-classical shape of the SEPL for binned TCSPC data for several bin numbers and IRF time constants, which shows that even for the number of bins as small as *N* = 64 and an IRF time constant as large as 1/6 of the laser period, the SEPL is very similar to $L_{\infty }$ (after calibration, see Sec. VIII).

#### 5. Discrete phasor of arbitrary periodic decays

##### a. Dirac IRF.

A detailed discussion of this case can be found in Appendix B.3, whose results we will now summarize.

In the discrete case, a useful basis of functions to decompose a *T*-periodic decay is in the set of functions ${\left\{\Lambda_{\tau ,T,N}\left(t\right)\right\}}_{\tau >0}$ proportional to the PSEDs defined in Eq. (17),

$$\Lambda_{\tau ,T,N}\left(t\right)=\frac{\tau }{\theta }\left(1-e^{-\theta /\tau }\right)\Lambda_{\tau ,T}\left(t\right),$$ (113)

where *θ* = *T*/*N* is the gate step. When *N* → *∞*, these functions are identical to the normalized PSEDs, ${\left\{\Lambda_{\tau ,T}\left(t\right)\right\}}_{\tau >0}$.

Any *T*-periodic function $F_{0,T}\left(t\right)$ can be written, after normalization by ${\left\|F_{0,T}\left(t_{p}\right)\right\|}_{N}$ (i.e., ${\left\|\right\|}_{N}$-normalization) [Eq. (92)],

$$f_{0,T}\left(t\right)=\overset{\infty }{\underset{0}{\int }}d\tau \mu_{0}\left(\tau \right)\Lambda_{\tau ,T,N}\left(t\right),$$ (114)

where the ${\left\|\right\|}_{N}$-*normalized* weight function $\mu_{0}\left(\tau \right)$ also appears in the expression for the phasor of $F_{0,T}\left(t\right)$,

$$z_{N}\left[f_{0,T}\right]=\overset{\infty }{\underset{0}{\int }}d\tau \mu_{0}\left(\tau \right)\zeta_{f,N}\left(\tau \right),$$ (115)

where the *canonical discrete phasors* of *T*-PSEDs, $\zeta_{f,N}\left(\tau \right)$, are defined in Eq. (99).

In particular, for a linear combination of PSEDs,

$$F_{0,T}\left(t\right)=\overset{n}{\underset{i=1}{\sum }}a_{i}\tau_{i}\Lambda_{\tau_{i},T}\left(t\right),$$ (116)

the ${\left\|\right\|}_{N}$-*normalized* weight function $\mu_{0}\left(\tau \right)$ reads

$$\left\{\begin{array}{c} \mu_{0}\left(t\right)=\overset{n}{\underset{i=1}{\sum }}\mu_{i}\delta \left(\tau -\tau_{i}\right),\\ \mu_{i}=\frac{a_{i}}{\left(1-e^{-\theta /\tau_{i}}\right)}/\overset{n}{\underset{j=1}{\sum }}\frac{a_{j}}{\left(1-e^{-\theta /\tau_{j}}\right)}. \end{array}\right.$$ (117)

In other words, the discrete phasor of a linear combination of single-exponential decays can be expressed as a linear combination of phasors, but, in order for the same functional form to be preserved, the discrete decay needs to be expressed in the basis of ${\left\{\Lambda_{\tau_{i},T,N}\left(t\right)\right\}}_{i=1\cdot ...n}$. The discrete phasor of the total decay is then expressed in the same functional form using their individual phasors, ${\left\{\zeta_{f,N}\left(\tau_{i}\right)\right\}}_{i=1\cdot ...n}$.

##### b. Arbitrary IRF.

The case of arbitrary IRFs will be discussed in the context of phasor calibration in Sec. VIII C.

## IV. THE EFFECT OF DECAY OFFSET

So far, we have assumed that time 0 of the recording was identified with the IRF maximum location *t*_0_, but this is not the case, in general, for various experimental reasons. For instance, when using TCSPC hardware based on the time-to-amplitude converter (TAC), it is customary to artificially delay the detector recording window with respect to the laser pulse signal, in order that photons emitted quasi-simultaneously with the excitation pulse are associated with a non-zero time-stamp: this allows avoiding the short and long time delay regions of the electronics, which are associated with the largest uncertainties or artifacts. In the general case, however, the experimentalist may simply choose to offset the location of the rising part of the recorded signal away from time 0 of the electronics so that the rising part of the signal is clearly visible. Yet another reason why the time stamp corresponding to the IRF maximum might not be precisely known could be that some of the gates are discarded for one reason or another, or the timestamp assigned to the first gate is set to a non-zero value. While such a general offset is easily handled in fluorescence decay fitting approaches by incorporating an additional offset parameter, the result of phasor calculation according to Eq. (63) (continuous phasor) or Eq. (88) (discrete phasor) leads to phasor properties that depend on the precise value of the offset and, in general, differ from those discussed so far.

One trivial option to avoid these changes is to first subtract the offset from the recorded data timestamps,

$$t\mapsto t^{\prime }=t-t_{0},$$ (118)

and if the resulting timestamps are negative, use the periodicity *T* of the decay to correct them,

$$t\mapsto t^{\prime }=\left(t-t_{0}\right)\left[T\right],$$ (119)

where “[T]” indicates the modulo-*T* operation. This operation, which amounts to a periodic shift of the decay, yields corrected timestamps with which the formulas derived in Sec. III can be used. However, this approach requires determining the exact value of the offset. This can in principle be done by recording the excitation (laser) signal as detected by the system, but this procedure might not be always practical, for instance, because the laser signal is efficiently rejected by the detection system and its signal can therefore not be recorded. In those cases, obtaining expressions corresponding to those derived in Sec. III, modified by the presence of an offset, allows carrying out phasor analysis without prior knowledge of the offset [that is using Eq. (63) or Eq. (88)] and interpret the results in light of the formulas derived next.

### A. Periodic decays with offset

The effect of an offset on the results derived in Sec. II is simply to replace any formula with a time argument *t* by the same formula with the substitution defined by Eq. (119). For instance, the expression for a PSED with lifetime *τ* and period *T* [Eq. (17)] is modified into

$$\Lambda_{\tau ,T|t_{0}}\left(t\right)≜\frac{1}{\tau \left(1-e^{-T/\tau }\right)}e^{-\left(t-t_{0}\right)\left[T\right]/\tau }=\Lambda_{\tau ,T}\left(t-t_{0}\right)$$ (120)

with an integral of 1 over [0, *T*]. Similarly, the expression for a square-gated PSED [Eq. (47)] is replaced by

$$\Lambda_{\tau ,T,W|t_{0}}\left(t\right)≜\left\{\begin{array}{cc} \left(a\right)\;\frac{1-e^{-\omega /\tau }}{1-e^{-T/\tau }}e^{-\left(t-t_{0}\right)\left[T\right]/\tau }+k,\; & \left(t-t_{0}\right)\left[T\right]\in [0,T-\omega [\\ \left(b\right)\;\frac{1-e^{-\left(\omega -T\right)/\tau }}{1-e^{-T/\tau }}e^{-\left(t-t_{0}\right)\left[T\right]/\tau }+k+1,\; & \left(t-t_{0}\right)\left[T\right]\in [T-\omega ,T[, \end{array}\right.$$

where

$$\left\{\begin{array}{c} k=\left\lfloor W/T\right\rfloor ,\;\\ \omega =W\left[T\right]=W-kT.\; \end{array}\right.$$ (121)

More generally, for an arbitrary *T*-periodic IRF $I_{T}\left(t\right)$ expressed in terms of the normalized $\Lambda_{\tau ,T}\left(t\right)$ [Eq. (25)],

$$I_{T|t_{0}}\left(t\right)=\overset{\infty }{\underset{0}{\int }}d\tau \xi_{0}\left(\tau \right)\Lambda_{\tau ,T|t_{0}}\left(t\right),$$ (122)

and for any PSED $\Lambda_{\tau_{0},T}\left(t\right)$ convolved with such an IRF [see Appendix D.1.2, Eq. (D5)],

$$\begin{array}{ll} I_{T|t_{0}}\underset{T}{*}\Lambda_{\tau_{0},T}\left(t\right) & =\overset{\infty }{\underset{0}{\int }}d\tau \xi_{0}\left(\tau \right)\Lambda_{\tau ,T|t_{0}}\underset{T}{*}\Lambda_{\tau_{0},T}\left(t\right)\\ & =\overset{\infty }{\underset{0}{\int }}d\tau \xi_{0}\left(\tau \right)\Psi_{\tau ,\tau_{0},T|t_{0}}\left(t\right). \end{array}$$ (123)

Likewise, for the square-gated version of such a decay [see Appendix D.1.2, Eq. (D21)],

$$\begin{array}{ll} I_{T|t_{0}}\underset{T}{*}\Lambda_{\tau_{0},T,W}\left(t\right) & =\overset{\infty }{\underset{0}{\int }}d\tau \xi_{0}\left(\tau \right)\Lambda_{\tau ,T|t_{0}}\underset{T}{*}\Lambda_{\tau_{0},T,W}\left(t\right)\\ & =\overset{\infty }{\underset{0}{\int }}d\tau \xi_{0}\left(\tau \right)\Psi_{\tau ,\tau_{0},T,W|t_{0}}\left(t\right). \end{array}$$ (124)

For an arbitrary gate shape, the corresponding formal expression is

$$I_{T|t_{0}}\underset{nT}{*}{\bar{\Gamma }}_{W,nT}\underset{T}{*}\Lambda_{\tau_{0},T}\left(t\right)=\overset{\infty }{\underset{0}{\int }}d\tau \xi_{0}\left(\tau \right)\;{\bar{\Gamma }}_{W,nT}\underset{nT}{*}\Psi_{\tau ,\tau_{0},T,W|t_{0}}\left(t\right),$$ (125)

which may or may not be simplified.

Using these expressions, it is relatively simple to obtain the modified formulas for the phasor in the different situations explored in Sec. III. The results are discussed next. As before, we will distinguish between continuous and discrete phasors and present examples of ungated and square-gated PSEDs.

### B. Continuous phasor of PSEDs with offset

#### 1. Continuous phasor of ungated PSEDs

##### a. PSEDs with Dirac IRF with offset.

It is easy to verify that the continuous phasor of a PSED defined by Eq. (120) is given by (Appendix A)

$$z\left[\Lambda_{\tau ,T|t_{0}}\right]=\frac{1}{1-i2\pi f\tau }e^{i2\pi ft_{0}}=\zeta_{f}\left(\tau \right)e^{i2\pi ft_{0}}.$$ (126)

In other words, it is equal to the phasor of a PSED without offset, $\zeta_{f}\left(\tau \right)$, rotated about 0 by an angle 2*πft*_0_.

##### b. PSEDs with single-exponential IRF with offset.

Details of the calculations are provided in Appendix D.5.2, resulting in the following phasor expression [Eqs. (D16) and (D17)]:

$$\begin{array}{ll} z\left[\Psi_{\tau ,\tau_{\times },T|t_{0}}\right]\left(\;f\right) & =\frac{1}{1-i2\pi f\tau }\frac{1}{1-i2\pi f\tau_{\times }}e^{i2\pi ft_{0}}\\ & =\zeta_{f}\left(\tau_{\times }\right)\zeta_{f}\left(\tau \right)e^{i2\pi ft_{0}}\\ & =z\left[\Lambda_{\tau_{\times },T|t_{0}}\right]\zeta_{f}\left(\tau \right), \end{array}$$ (127)

where *τ*_×_ is the time constant of the single-exponential IRF. Once again, the phasor is equal to the phasor of the same PSED with single-exponential IRF without offset, $\zeta_{f}\left(\tau_{\times }\right)\zeta_{f}\left(\tau \right)$, rotated about 0 by an angle 2*πft*_0_.

#### 2. Continuous phasor of square-gated PSEDs with offset

Although the calculation is made a bit cumbersome, as two cases need to be distinguished (*t*_0_ < *ω* and *t*_0_ ≥ *ω*, see Appendix A, derivation of Eq. (A10) for details in the case of a Dirac IRF and a square gate), the result is again simply a rotation by an angle 2*πft*_0_ of the results in the absence of offset [Eqs. (75) and (78)].

For instance, for a Dirac IRF [Eq. (A10)],

$$\left\{\begin{array}{c} z_{\left[W\right]}\left[\Lambda_{\tau ,T|t_{0}}\right]≜z\left[\Lambda_{\tau ,T,W|t_{0}}\right]=M_{W}e^{-i\varphi_{W}}\zeta_{f}\left(\tau \right)e^{i2\pi ft_{0}}=z\left[{\bar{\Pi }}_{W,nT}\right]\zeta_{f}\left(\tau \right)e^{i2\pi ft_{0}},\\ M_{W}=\frac{\sin\varphi_{W}}{\pi fW},\;\varphi_{W}=\pi fW, \end{array}\right.$$ (128)

and for a single-exponential IRF with time constant *τ*_×_ [Eq. (D28)],

$$\begin{array}{ll} z_{\left[W\right]}\left[\Psi_{\tau ,\tau_{\times },T|t_{0}}\right] & ≜z\left[\Psi_{\tau ,\tau_{\times },T,W|t_{0}}\right]\\ & =M_{W}e^{-i\varphi_{W}}\zeta_{f}\left(\tau_{\times }\right)\zeta_{f}\left(\tau \right)e^{i2\pi ft_{0}}\\ & =z\left[{\bar{\Pi }}_{W,nT}\right]\zeta_{f}\left(\tau_{\times }\right)e^{i2\pi ft_{0}}\zeta_{f}\left(\tau \right). \end{array}$$ (129)

All these results are of the same general form,

$$\begin{array}{ll} z\left[I_{W,T|t_{0}}\underset{T}{*}\Lambda_{\tau ,T}\right] & =z\left[I_{T|t_{0}}\underset{nT}{*}{\bar{\Gamma }}_{W,nT}\underset{T}{*}\Lambda_{\tau ,T}\right]\\ & =z\left[{\bar{\Gamma }}_{W,nT}\right]z\left[I_{T|t_{0}}\right]\zeta_{f}\left(\tau \right), \end{array}$$ (130)

where the right hand side singles out the phasor of the (gated) instrument response function with offset. They also confirm that the effect of an offset *t*_0_ on the continuous phasor is simply a rotation by an angle 2*πft*_0_.

### C. Discrete phasor of PSEDs with offset

#### 1. Discrete phasor of ungated PSEDs with offset

Analytical results for discrete phasors of decays with offset are a bit more complicated to compute, in particular in the presence of gating, and differ from those for continuous phasors, which are characterized by their simplicity and universality. We discuss only a few cases that can be fairly simply calculated analytically.

##### a. PSEDs with Dirac IRF with offset.

In the case where the decay sampling points cover the whole period (*T* = *Nθ*), one obtains the following expression for the discrete phasor of an ungated PSED with a Dirac IRF [see Appendix B.1.2, Eq. (B26); an expression for the case where the decay samples do not cover the full period is also provided in Appendix B, Eq. (B27)]:

$$\left\{\begin{array}{c} z_{N}\left[\Lambda_{\tau ,T|t_{0}}\right]=\frac{1-x}{1-xe^{i\alpha }}e^{i\varphi_{N}\left(t_{0}\right)}=\zeta_{f,N}\left(\tau \right)e^{i\alpha \left\lceil t_{0}/\theta \right\rceil },\\ x\left(\tau \right)=e^{-\theta /\tau },\;\alpha =2\pi f\theta , \end{array}\right.$$ (131)

which is a rotated version of the expression $z_{N}\left[\Lambda_{\tau ,T}\right]$ obtained in the absence of offset [Eq. (99)].

The argument of the constant exponential factor on the right hand side of Eq. (131) states that if the offset $t_{0}\in [0,T[$ is a multiple of the gate step size *θ*, then the resulting phasor is simply a rotated version of the discrete phasor of the ungated PSED without offset [Eq. (99)], by an angle 2*πft*_0_.

If, however, the offset *t*_0_ is *not* a multiple of the gate step size *θ*, the resulting phasor is still a rotated version of the phasor without offset, but the angle is now given by a slightly different expression [see Appendix B, Eq. (B26) for a derivation]. In particular, this expression predicts that, in some cases, the phasor of identical decays with *different* offsets (within *θ* of each other) will be equal. This is, for instance, the case for *T* = 12.5 ns, *θ* = 1.25 ns (*N* = 10), and $\left.t_{0}\in \right]0,1.25$] ns, as can be verified by a direct calculation using Eqs. (92) and (120).

##### b. PSEDs with single-exponential IRF with offset.

The formula for the discrete phasor of an ungated PSED with a single-exponential IRF with time constant τ_*_ is derived in Appendix D [Eq. (D40)],

$$z_{N}\left[\Psi_{\tau ,\tau_{\times },T|t_{0}}\right]=\zeta_{f,N}\left(\tau \right)\zeta_{f,N}\left(\tau_{\times }\right)\Omega \left(\tau ,\tau_{\times },t_{0}\right)e^{i\left\lceil \frac{t_{0}}{\theta }\right\rceil \alpha },$$ (132)

where $\Omega \left(\tau ,\tau_{\times },t_{0}\right)$ is, in general, a complex function of *τ*, *τ*_×_, and *t*_0_, and *α* is defined as before [e.g., Eq. (131)]. This shows that the corresponding SEPL is not a simple curve.

If, however, the offset is commensurate with the gate step (*t*_0_ = *qθ*), this complex function $\Omega \left(\tau ,\tau_{\times },t_{0}\right)$ reduces to *e*^*iα*^ and the phasor can be written as the product of two phasors and a constant [Eq. (D42)],

$$t_{0}=q\theta \Rightarrow z_{N}\left[\Psi_{\tau ,\tau_{\times },T|t_{0}}\right]=z_{N}\left[\Lambda_{\tau ,T}\right]z_{N}\left[\Lambda_{\tau_{\times },T|t_{0}}\right]e^{i\alpha }.$$ (133)

The corresponding SEPL is therefore a rotated version of $L_{N}$.

#### 2. Discrete phasor of square-gated PSEDs with offset

The calculations in this situation are a bit cumbersome and require the distinction of different cases depending on the respective values of the offset, period, gate width, and gate step. We will look at the Dirac IRF case in some detail, limiting the discussion of the single-exponential IRF to a general formula and skipping the case of arbitrary IRF altogether.

##### a. Square-gated PSEDs with Dirac IRF with offset.

The expression for the discrete phasor of a square-gated PSED with Dirac IRF with offset is derived in Appendix B [Eq. (B60)],

$$\left\{\begin{array}{c} z_{N}\left[\Lambda_{\tau ,T,W|t_{0}}\right]=\frac{\frac{e^{ir\alpha }-e^{iq\alpha }}{1-e^{i\alpha }}+\frac{x^{q}e^{iq\alpha }-ux^{r}e^{ir\alpha }}{1-xe^{i\alpha }}e^{t_{0}/\tau }}{kN+q-r+\frac{x^{q}-ux^{r}}{1-x}e^{t_{0}/\tau }},\\ q=\left\lceil \frac{t_{0}}{\theta }\right\rceil ,r=\left\lceil \frac{t_{0}-\omega }{\theta }\right\rceil , \end{array}\right.$$ (134)

where $x\left(\tau \right)=e^{-\theta /\tau }$, $u\left(\tau \right)=e^{-\omega /\tau }$, and *α* = 2*πfθ* as before. This equation does not in general describe any simple algebraic curve, except in particular cases.

A special case of interest is encountered when the gates are adjacent (*W* = *θ*). Two different situations may occur:

*Case W* = *θ*, *t*_0_ = *qθ*

When the offset is proportional to the gate step (i.e., the offset falls on one of the gate starts), the discrete phasor reads

$$z_{N\left[\theta \right]}\left[\Lambda_{\tau ,T|t_{0}}\right]=\zeta_{f,N}\left(\tau \right)e^{i2\pi ft_{0}}=z_{N}\left[\Lambda_{\tau ,T|t_{0}}\right].$$ (135)

In other words, the discrete phasor of the square-gated PSED is then equal to the discrete phasor of the *ungated* version of the PSED, with the same offset. Consequently, $L_{N\left[\theta \right]}$ is a rotated arc of circle.

*Case W* = *θ*, *t*_0_ = *qθ* − *θ*_0_, 0 < *θ*_0_ < *θ*

When the offset is not proportional to the gate step (i.e., when the offset is distinct from any of the gate starts), the phasor reads

$$\begin{array}{ll} z_{N\left[\theta \right]}\left[\Lambda_{\tau ,T|t_{0}}\right] & =z_{N}\left[\Lambda_{\tau ,T,\theta |t_{0}}\right]\\ & =\frac{\left(1-x+\left(1-e^{i\alpha }\right)\left(x-e^{-\theta_{0}/\tau }\right)\right)}{1-xe^{i\alpha }}e^{i\left(q-1\right)\alpha }. \end{array}$$ (136)

**FIG. 7.**
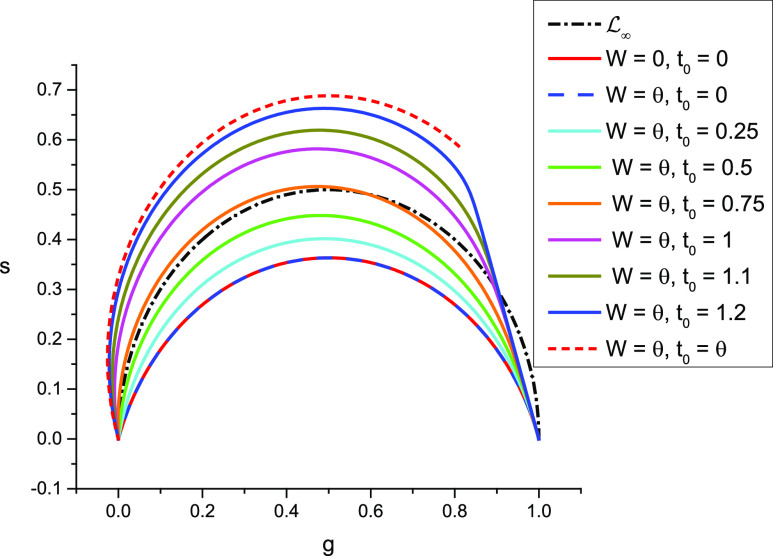
Effect of a decay offset on the $L_{N\left[W\right]}$ in the simple case where the gate width *W* is equal to the gate step *θ*. The calculations were made with *T* = 12.5 ns, *N* = 10 (i.e., *θ* = 1.25). The standard $L_{\infty }$ (semi-circle) is represented as a black dotted-dashed curve. $L_{N}$ (plain red curve) and $L_{N\left[\theta \right]}$ (plain dark blue curve) are identical, yielding a red/dark blue dashed circular arc. As the decay offset *t*_0_ increases (with *t*_0_ < *θ*), $L_{N\left[W\right]}$ progressively rotates toward and is deformed into $L_{N\left[\theta \right]}$, which is a circular arc (red dashed curve). Note that when *t*_0_ ∼ *θ*, $L_{N\left[\theta \right]}$ looks like a circular arc with a straight stem connecting to the 0-lifetime phasor $z_{N\left[W\right]}\left[\Lambda_{0,T}\right]$ = 0.

This expression does not describe any simple curve but can be studied numerically, as illustrated in Fig. 7, in which *q* = 0, and *t*_0_ was incremented from 0 to *θ*. As *t*_0_ is increased, $L_{N\left[\theta \right]}$ progressively deforms from the circular arc corresponding to $L_{N}$ for *t*_0_ = 0 into that corresponding to *t*_0_ = *θ*, with a quasi-linear “stem” overlapping the segment connecting $z_{N}\left[\Lambda_{0,T}\right]$ to $z_{N}\left[\Lambda_{0,T|\theta }\right]$. This scenario is repeated with each additional increment of *θ* to *t*_0_, with the circular arc ($L_{N}$) corresponding to *t*_0_ = *qθ* replacing the $L_{N}$ corresponding to *t*_0_ = 0.

This discussion shows that the SEPL can have rather odd shapes if the decay offset *t*_0_ does not correspond to gate starts. This problem is minimized if the number of gates N is large, since in this case, the difference between $L_{N\left[\theta \right]}$’s for *t*_0_ = *qθ* and $t_{0}=\left(q+1\right)\theta$ is a rotation of $2\pi f\theta =2\pi \frac{n}{N}$, which is a small angle.

##### b. Square-gated PSEDs with single-exponential IRF with offset.

The expression for the discrete phasor of a square-gated PSED with single-exponential IRF with offset is derived in Appendix D [Eq. (D53)]. It does not correspond to a simple curve but is useful to study the effect of the different parameters $\left(\theta ,W,\tau_{\times },t_{0}\right)$ on the shape of the SEPL.

## V. THE EFFECT OF DECAY TRUNCATION

So far, we have assumed that the recorded decay comprises *N* equidistant data points, which cover the whole laser period *T* (*Nθ* = *T*). However, this might not always be the case experimentally, for various reasons. For instance, if the laser period is much longer than most of the lifetimes encountered in a study, and if each data point requires a long acquisition time, it might be advantageous to dispense with recording data for gates starting past a few times the largest lifetime after the laser pulse (i.e., offset *t*_0_). Alternatively, the user may decide to skip the first few gates based on the argument that they are the most affected by the IRF or do both (truncation on both sides of the laser period window).

While such a truncated decay may provide sufficient data for a good fit of the decay with a mono- or multi-exponential model, calculating its phasor based on Eq. (88) will in general result in a phasor that does not behave as described in the Secs. III and IV. In particular, the choice of the phasor harmonic frequency *f* as a multiple of the fundamental frequency *T*^−1^ turns out to be a poor choice in general.

As will soon become clear, an analytical expression of the phasor in the general truncated case is not particularly illuminating, and it is more efficient to analyze the effect of truncation numerically, starting from the easily calculated equation  (88), which we will do in the examples discussed in Sec. IX. Here, we will limit ourselves to the case of the continuous and discrete phasors of ungated PSED with Dirac excitation, as they provide some insight into the different points discussed above. The situation of the discrete phasor of truncated square-gated PSED will be studied only in a single case where the phasor takes a simple form.

### A. Continuous phasor of truncated ungated PSEDs

We define the truncated decay by its initial recording position, *t*_1_ ≥ 0, and the total “span” of the record, *D* = *Nθ*, such that *t*_1_ + *D* ≤ *T*. The definitions of $\left\|S_{T}\left(t\right)\right\|$ and the numerator of the corresponding “truncated” phasor $\overset{↔}{z}\left[S_{T}\right]$ are given by a trivial modification of Eq. (63),

$$\left\{\begin{array}{c} \overset{↔}{z}{\left[S_{T}\right]}_{t_{1},D}=\frac{{\left\|S_{T}\left(t\right)e^{i2\pi ft}\right\|}_{t_{1},D}}{{\left\|S_{T}\left(t\right)\right\|}_{t_{1},D}},\\ {\left\|S_{T}\left(t\right)e^{i2\pi ft}\right\|}_{t_{1},D}=\overset{t_{1}+D}{\underset{t_{1}}{\int }}dt\;S_{T}\left(t\right)e^{i2\pi ft},\\ {\left\|S_{T}\left(t\right)\right\|}_{t_{1},D}=\overset{t_{1}+D}{\underset{t_{1}}{\int }}dt\;S_{T}\left(t\right), \end{array}\right.$$ (137)

where we have used a double-ended arrow above the phasor symbol, $\overset{↔}{z}$, to indicate that the start (*t*_1_) and end (*t*_1_ + *D*) of the integration are non-standard. Subscripts “*t*_1_, *D*” added to all quantities indicate the value of the first gate start and the record span. It is easy to verify that for a PSED excited by a Dirac pulse [Eq. (17)],

$$\left\{\begin{array}{c} \overset{↔}{z}{\left[\Lambda_{\tau ,T}\right]}_{t_{1},D}=\frac{1-\eta e^{i2\pi fD}}{1-\eta }e^{i2\pi ft_{1}}\zeta_{f}\left(\tau \right),\\ \eta \left(\tau \right)=e^{-D/\tau }. \end{array}\right.$$ (138)

This expression shows that the continuous phasor of a truncated PSED with Dirac excitation is equal to the canonical phasor $\zeta_{f}\left(\tau \right)$ rotated about the origin by a lifetime-dependent angle $2\pi f\left(t_{1}-\beta \right)$ and scaled by a lifetime-dependent factor $\lambda \left(\tau \right)$ given by

$$\left\{\begin{array}{c} \overset{↔}{z}\left[\Lambda_{\tau ,T}\right]=\lambda e^{i2\pi f\left(t_{1}-\beta \right)}\zeta_{f}\left(\tau \right),\\ \beta \left(\tau \right)=\tan^{-1}\frac{\eta \sin\left(2\pi fD\right)}{1-\eta \cos\left(2\pi fD\right)},\\ \lambda \left(\tau \right)=\frac{{\left(1-2\eta \cos\left(2\pi fD\right)+\eta^{2}\right)}^{\frac{1}{2}}}{1-\eta }. \end{array}\right.$$ (139)

The asymptotic behaviors of *β* and *λ* are as follows:

- For *τ* → 0, $\eta \left(\tau \right)\to 0$ and therefore $\beta \left(\tau \right)\to 0$: the right hand side of Eq. (138) tends to $e^{i2\pi ft_{1}}\zeta_{f}\left(\tau \right)$, located on a rotated version of the *UC*_*∞*_.
- For $\tau \to \infty ,\;\eta \left(\tau \right)\to 1$, and thus, $\tan\beta \left(\tau \right)\to \cot\pi fD$, while $\lambda \left(\tau \right)\to 2\left|\sin\pi fD\right|\tau /D$. Because $\zeta_{f}\left(\tau \right)\to e^{i\varphi \left(\tau \right)}/2\pi f\tau$ [see Eq. (71)], the result is:

$$\overset{↔}{z}{\left[\Lambda_{\infty ,T}\right]}_{t_{1},D}=\frac{\left|\sin\left(\pi fD\right)\right|}{\pi fD}e^{i\pi fD}e^{i2\pi ft_{1}},$$ (140)

a value which is in general different from 0.

**FIG. 8.**
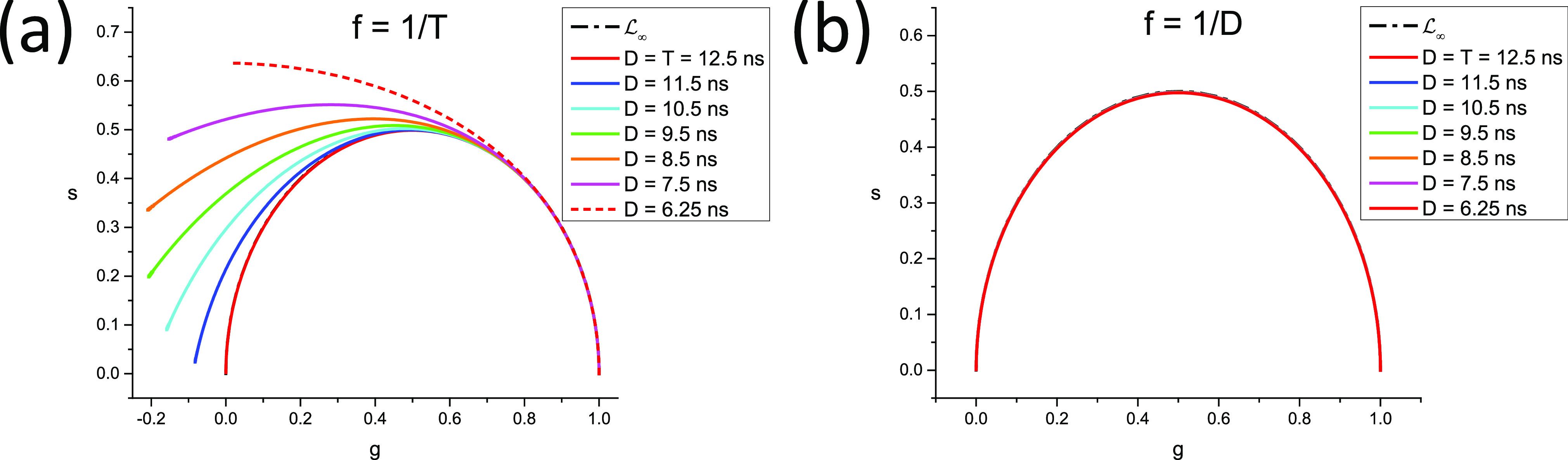
Continuous phasor of truncated decays. To illustrate the effect of decay truncation, we use *T* = 12.5 ns and compute the loci of continuous phasors of ungated PSED (SEPL) as a function of the observation duration *D* ≤ *T* when f = 1/*T* (a) or f = 1/*D* (b). In the latter case, all curves are identical to $L_{\infty }$. However, if the phasor frequency is chosen to be the fundamental Fourier frequency f = 1/*T*, the SEPL increasingly departs from the $L_{\infty }$ as the observation duration *D* decreases. For *D* = *T*/2, $z_{t_{1},D}\left[\Lambda_{\infty ,T}\right]$ ends up at the vertical of the 0 point (the location of $z\left[\Lambda_{\infty ,T}\right]$ in $L_{\infty }$).

Figure 8 illustrates, without loss of generality, these properties in the special case *t*_1_ = 0, as *t*_1_ ≠ 0 simply adds a constant rotation [Eq. (138)]. In that particular case, we truncated the decay down to *D* = *T*/2, for which Eq. (140) yields $\overset{↔}{z}{\left[\Lambda_{\infty ,T}\right]}_{0,T/2}=i2/\pi$, which is located at the vertical of the locus of infinite lifetime in the standard $L_{\infty }$ (*z* = 0).

It is also clear from Eq. (138) that if f=n/D,  n∈N, the first term $\frac{1-\eta e^{i2\pi fD}}{1-\eta }$ is equal to 1 and we are left with a rotated version of the continuous phasor of non-truncated ungated PSED [Eq. (99)],

$$f=\frac{n}{D}\Rightarrow \overset{↔}{z}{\left[\Lambda_{\tau ,T}\right]}_{t_{1},D}=e^{i2\pi ft_{1}}\zeta_{f}\left(\tau \right).$$ (141)

This phasor frequency does not belong to the series of Fourier harmonics associated with *T*-periodic decays, ${\left\{n/T\right\}}_{n>0}$, but since it leads to a simpler functional form of $\overset{↔}{z}{\left[\Lambda_{\tau ,T}\right]}_{t_{1},D}$, and therefore a simpler interpretation of the calculated phasor, it is a natural choice to adopt.

### B. Discrete phasor of truncated ungated PSEDs

The discrete phasor of a truncated decay is defined by Eq. (88), in which 0 ≤ *t*_1_ < *t*_*N*_ ≤ *T* (we will assume that the gates are equidistant: $\theta_{p}=\theta ,\;p\in \left[1,N\right]$) and they span *D* = *Nθ* < *T*.

In the only case we will treat analytically, that of a PSED in the presence of a Dirac IRF, a straightforward calculation yields

$$\begin{array}{ll} {\overset{↔}{z}}_{N}{\left[\Lambda_{\tau ,T}\right]}_{t_{1},D} & =\frac{1-x^{N}e^{iN\alpha }}{1-x^{N}}\frac{1-x}{1-xe^{i\alpha }}e^{i2\pi ft_{1}}\\ & =\frac{1-x^{N}e^{iN\alpha }}{1-x^{N}}e^{i2\pi ft_{1}}\zeta_{f,N}\left(\tau \right), \end{array}$$ (142)

where we have used the previous notations $x\left(\tau \right)=e^{-\theta /T},\;\alpha =2\pi f\theta$, and $\zeta_{f,N}\left(\tau \right)$ is the discrete phasor of a non-truncated, ungated PSED [Eq. (99)]. The prefactor in front of the term $e^{i2\pi ft_{1}}\zeta_{f,N}\left(\tau \right)$ in Eq. (142) depends on *τ* and therefore shows that the SEPL is in general complex, unless *Nα* = 2*nπ*, i.e., f=n/D,  n∈N.

We can therefore distinguish two situations:

#### 1. f = n/D, n∈N

As in the continuous case, if f=n/D,  n∈N, the fractional prefactor in Eq. (142) is equal to 1 and we are left with a rotated version of the discrete phasor of non-truncated ungated PSED with Dirac excitation [Eq. (99)], which is an arc of circle rotated about the origin,

$$f=\frac{n}{D}\Rightarrow {\overset{↔}{z}}_{N}{\left[\Lambda_{\tau ,T}\right]}_{t_{1},D}=e^{i2\pi ft_{1}}z_{N}\left[\Lambda_{\tau ,T}\right].$$ (143)

#### 2. General case, f ≠ n/D

In the general case, the fractional prefactor in Eq. (142) can be rewritten, $\lambda_{N}e^{-i\beta_{N}}$, with

$$\left\{\begin{array}{c} \lambda_{N}\left(\tau \right)=\frac{{\left(1-2x^{N}\cos N\alpha +x^{2N}\right)}^{\frac{1}{2}}}{1-x^{N}},\\ \beta_{N}\left(\tau \right)=\tan^{-1}\frac{x^{N}\sin N\alpha }{1-x^{N}\cos N\alpha }. \end{array}\right.$$ (144)

This prefactor depends on *τ*, and its asymptotic behavior when *τ* → 0 and *τ* → *∞* is easy to compute:

- When *τ* → 0, *λ*_*N*_ → 1, and *β*_*N*_ → 0, therefore, the phasor ${\overset{↔}{z}}_{N}{\left[\Lambda_{\tau ,T}\right]}_{t_{1},D}$ tends to the expression of Eq. (143), which defines a rotated circular arc.
- When *τ* → *∞*, *x* ∼ 1 −*θ*/*τ* and *x*^*N*^ ∼ 1 − *Nθ*/*τ*, which leads to the asymptotic expression

$${\overset{↔}{z}}_{N}{\left[\Lambda_{\tau ,T}\right]}_{t_{1},D}\underset{\tau \to \infty }{-\;-\;\to }\frac{1}{N}\frac{1-e^{iN\alpha }}{1-e^{i\alpha }}e^{i2\pi ft_{1}}.$$ (145)

This value is different from 0 when *f* ≠ *n*/*D*. It is easy to verify that in the limit *N* → *∞*, we recover Eq. (140).

Overall, we see that the curve described by Eq. (142) is close to a rotated arc of circle [Eq. (143)] when *τ* → 0 and ends up on a point ${\overset{↔}{z}}_{N}{\left[\Lambda_{\infty ,T}\right]}_{t_{1},D}$, which is, in general, different from the origin.

### C. Discrete phasor of truncated square-gated PSEDs with offset

This situation is the most complicated, but also the most general, and, as in the previous discussion, can be simplified with an adequate choice of starting gate position and phasor frequency.

As shown in Appendix B, Sec. B.2.4.b, in the case of a positive offset ($\left.t_{0}\in \right]0,T$[) and first gate chosen to start at the decay offset, the phasor reads, assuming a phasor harmonic frequency proportional to *D*^−1^,

$$\begin{array}{ll} & \left.\begin{array}{c} t_{1}=t_{0},\;t_{N}<T-\omega ,\\ f=\frac{n}{D} \end{array}\right\}\\ & \Rightarrow \left\{\begin{array}{c} {\overset{↔}{z}}_{N\left[W\right]}{\left[\Lambda_{\tau ,T}\right]}_{t_{0},D}=\frac{1-x}{1-xe^{i\alpha }}e^{i2\pi ft_{0}}=e^{i2\pi ft_{0}}\zeta_{f,N}\left(\tau \right),\\ x\left(\tau \right)=e^{-\theta /\tau },\;\alpha =2\pi f\theta . \end{array}\right. \end{array}$$ (146)

This is the same equation as for the discrete phasor of an ungated PSED and describes a circular arc rotated about the origin. Note that this simple formula is valid only if the phasor frequency is a multiple of *D*^−1^ (it is not correct if *f* is chosen to be a multiple of *T*^−1^ instead, see Sec. B.2.4.b).

**FIG. 9.**
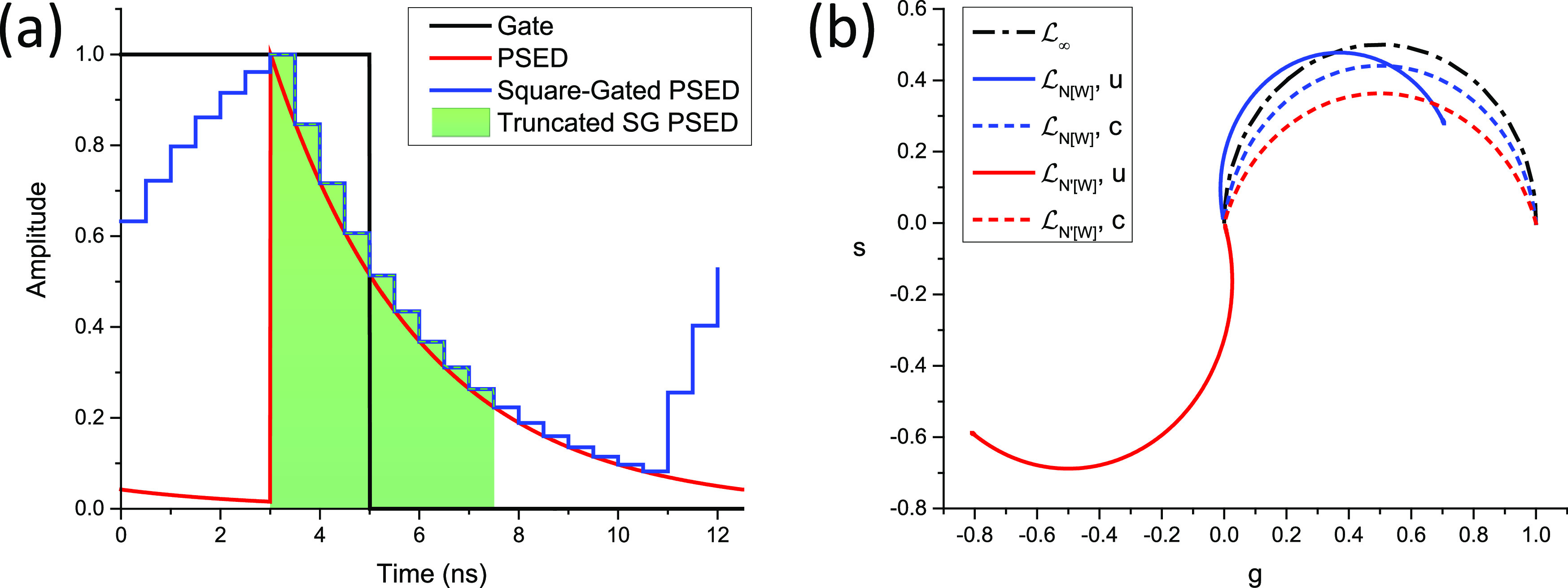
Discrete phasor of an offset and truncated square-gated PSED. (a) Square Gate (*W* = 5 ns, black), periodic single-exponential decay (PSED, *τ* = 3 ns, red), gated PSED sampled every *θ* = 0.5 ns (*N* = 25, blue), and truncated version (*N* = 10, green) starting at *t*_1_ = 3 ns, the position of the laser IRF, and ending at *t*_*N*_ = *T* − *W*. The laser period is *T* = 12.5 ns. The “duration” of the truncated decay is *D* = *T* − *W* − *t*_0_ + *θ* = 5 ns. (b) SEPLs for the PSED shown in (a). Blue: full PSED, f = 1/*T*, red: truncated PSED, f = 1/*D*. Uncalibrated SEPLs are shown as solid curves, while calibrated (*τ*_*C*_ = 0 ns) SEPLs are shown as dashed curves.

In other words, as shown in Fig. 9, in the case of a discrete square-gated PSED with offset, it might be advantageous to make sure that the IRF location *t*_0_ coincides with the start of a gate, chosen as the starting gate (*t*_1_ = *t*_0_), and truncate the recording at gate *N* such that

$$N\leq \left\lfloor \frac{T-\omega -t_{0}}{\theta }\right\rfloor .$$ (147)

Using a phasor frequency *f* = *n*/*Nθ* = *n*/*D* will result in a phasor

$$z_{N\left[W\right]}\left[\Lambda_{\tau ,T}\right]=\frac{1-x}{1-xe^{i\alpha }}e^{i2\pi ft_{0}}=e^{i2\pi ft_{0}}\zeta_{f,N}\left(\tau \right),$$ (148)

that is, an arc of circle rotated about the origin.

## VI. THE EFFECT OF GATE SHAPE

In Secs. III–V, we have used square gates as an example of gate shape that can be easily treated analytically, at least in the case of simple excitation pulse shapes. This has allowed us to study the effect of gate width on the phasors of PSEDs. This model is adapted to data acquired with photon-counting detectors followed by electronics that effectively bin photons or to study the effect of binning on such data. For instruments whose response is actually electronically gated (turned on and off), the resulting detection efficiency temporal profile is rarely rectangular (or “square”). In practice, the finite temporal resolution of the response leads to smooth instead of sharp edges and potentially to ringing or irregular rather than flat top. Examples of such a departure from ideality can be found in the literature (e.g., Refs. 28, 29, and 32). In other cases, the electronic “gating” profile might simply not be rectangular at all but instead triangular or ramp-like or even sinusoidal, among many possible examples.

Note that it is also possible to modulate the phase of the modulation of the detector response in some frequency modulation approaches.^33,34^ While this may give rise to interesting modulation shapes, these are not directly relevant to the topic of this article, concerned exclusively with fixed phase (or offset) gate functions.

### A. Effect of gate shape on continuous phasors

In the case of continuous phasors, the effect of gating on the phasor is indeed trivial due to the fact that

(i) a gate’s effect on a decay can be described as an additional term in a convolution product [Eq. (42)] and
(ii) the convolution rule [Eq. (69)] shows that this gate term amounts to multiplying the phasor by a constant term.

In other words, two experiments differing only by their gate shapes will result in phasors that differ only by a constant complex scaling factor, i.e., a dilation and a rotation of the universal circle ($L_{\infty }$). As discussed in Sec. VIII, this difference is taken care of by phasor calibration.

### B. Effect of gate shape on discrete phasors

The difference between SEPLs corresponding to different gate shapes is more subtle for discrete phasors. Indeed, we have seen in Sec. III C 4 that even in the case of a simple square gate and Dirac excitation, the shape of the SEPL can change significantly by a mere change in gate width. This effect will be more noticeable for smaller number of gates.

**FIG. 10.**
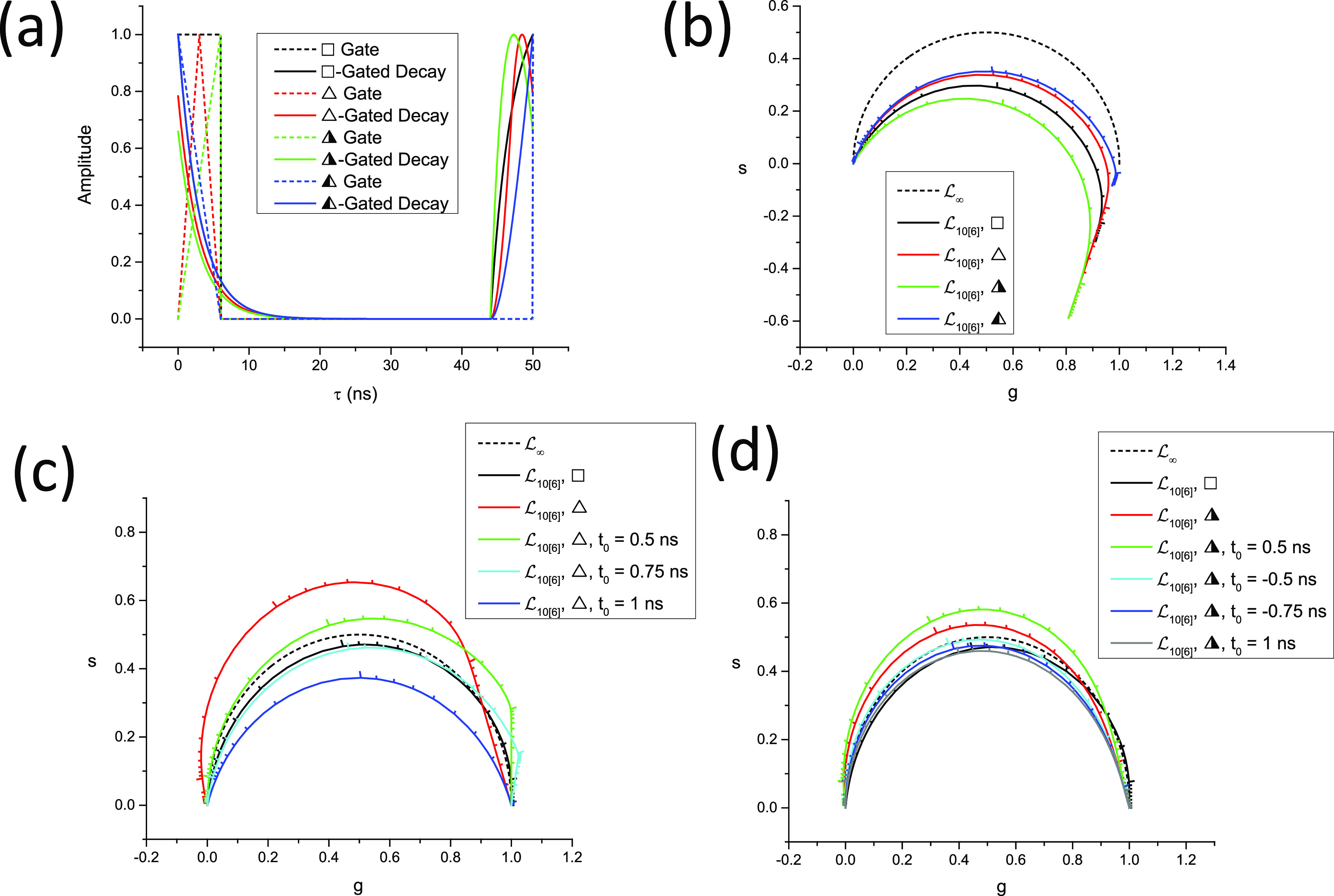
Effect of gate shapes on the discrete phasors. (a) 4 gates of width *W* = 6 ns (square, triangle, sawtooth, and reversed sawtooth, dashed curves) are shown starting at *t* = 0 within a period of duration *T* = 50 ns (f = 20 MHz). The corresponding gated-decays for a PSED with *τ* = 3 ns are shown as plain curves. Due to the different locations of the maximum of each gate, the corresponding *T*-periodic gated decays exhibit different maxima locations as well as different shapes. This effect is similar for all PSEDs and therefore results in different SEPL shown in (b). (b) Universal semicircle ($L_{\infty }$, dashed curve) and the 4 SEPL with gate width *W* = 6 ns for the same number of equidistant gate locations *N* = 10. While the SEPLs look fairly similar, noticeable differences exist. Ticks indicate the locations of PSEDs with lifetime 0.1–1 in steps of 0.1, 1–10 in steps of 1, etc., with ticks corresponding to 1, 10, and 100 drawn slightly longer. (c) By shifting the triangle gate or equivalently, introducing a positive IRF offset *t*_0_ (indicated in the legend), the difference between the calibrated SEPL and that corresponding to the square gate (black curve) can be minimized if not completely eliminated. (d) A similar but negative adjustment of the sawtooth gate offset achieves a similar better similarity between the SEPL corresponding to the square gate (black curve) and the sawtooth one (offset indicated in the legend).

A numerical comparison of the SEPLs obtained for a few gate shapes can be instructive and is presented in Fig. 10. Figure 10(a) shows 4 examples of *W* = 6 ns-wide gate shapes (square, triangle, sawtooth, and reversed sawtooth) and their effect on a *τ* = 3 ns PSED. All broaden the decay but also shift and deform it in different ways. This results in different discrete SEPLs computed for *N* = 10 gates for the 4 gate shapes, as shown in Fig. 10(b). These differences are not overly surprising, considering that the gates may effectively shift the IRF differently. Figures 10(c) and 10(d) illustrate this point by showing that by adding or subtracting some IRF offset, the SEPL of triangle-gated decays [Fig. 10(c)] or of sawtooth-gated decays [Fig. 10(d)] can be somewhat (but not perfectly) made to look closer after proper rotation and rescaling so that the phasors of the 0-lifetime PSEDs are all located at 1 (i.e., after proper phasor calibration, as discussed in detail in Sec. VIII).

While these observations are merely qualitative, they suggest that, in general, gates of similar duration (i.e., support size *W*) result in similar SEPL shapes, provided that the proper IRF shift is implemented.

More quantitative estimates of the effects of small gate shape variations on the calculated phasors can of course be obtained in case an analytical expression for the phasor is available, such as those provided in this work. We give an example of such an analysis in Sec. IX C 2 when discussing the effect of gate width variations on the phase and modulus lifetime. The necessary expressions are derived in Sec. VII.

## VII. PHASE AND MODULUS LIFETIMES

### A. General considerations

Equipped with this better understanding of the differences between continuous vs discrete phasors of ungated and square-gated PSED, the effect of complete or truncated recording, IRF offset, and various combinations thereof, we can now look into ways to use the computed phasors to gain information on the recorded decays.

In the “standard” phasor analysis, by which term we mean the analysis of *continuous* phasors of *ungated* decays excited by a Dirac IRF, definition (70) of the phasor $\zeta_{f}\left(\tau \right)$ of a PSED with lifetime *τ* can be rewritten, $\zeta_{f}\left(\tau \right)=me^{i\varphi }$, as in Eq. (71), where

$$\left\{\begin{array}{c} m\left(\tau \right)=\frac{1}{\sqrt{1+{\left(2\pi f\tau \right)}^{2}}},\;\\ \varphi \left(\tau \right)=\tan^{-1}\left(2\pi f\tau \right), \end{array}\right.$$ (149)

defining the phasor phase *φ* and modulus *m*. For PSED, this leads to two equivalent expressions for the lifetime *τ*: the *phase lifetime τ*_*φ*_ and the *modulus lifetime τ*_*m*_, given by^13,35^

$$\left\{\begin{array}{c} \tau_{\varphi }=\frac{1}{2\pi f}\tan\varphi ,\\ \tau_{m}=\frac{1}{2\pi fm}\sqrt{1-m^{2}}. \end{array}\right.$$ (150)

These expressions can be used formally with the modulus and phase of the phasor of non-single-exponential decays as well, but in that case, the two values *τ*_*φ*_ and *τ*_*m*_ are likely to (i) be different from one another, and (ii) their interpretation will be ambiguous at best. In particular, if the phasor of a decay $S_{T}\left(t\right)$ is outside $L_{\infty }$, i.e., *m* > 1, *τ*_*m*_ given by Eq. (150) will be imaginary (due to the presence of the square root of a negative number). No such problem exists for *τ*_*φ*_, but the result should be interpreted cautiously, as any decay whose phasor is not located on the universal circle is obviously not a PSED. In particular, *τ*_*φ*_ is, in general, different from the average lifetime,

$$\left\{\begin{array}{c} \tau_{\varphi }\neq \left\langle \tau \right\rangle =\frac{\overset{\infty }{\underset{0}{\int }}dt\;t\;F_{0}\left(t\right)}{\overset{\infty }{\underset{0}{\int }}dt\;F_{0}\left(t\right)}=\frac{{\left\|F_{0,T}\left(t\right)\right\|}_{T}}{{\left\|F_{0,T}\left(t\right)\right\|}_{T}},\\ F_{0}\left(t\right)=-\overset{t}{\int }du\;F_{0}\left(u\right), \end{array}\right.$$ (151)

where $F_{0}\left(t\right)$ is the signal emitted upon a Dirac excitation, $F_{0,T}\left(t\right)$ its *T*-periodic summation, $-F_{0}\left(t\right)$ is its primitive, and $F_{0,T}\left(t\right)$ is the *T*-periodic summation of $F_{0}\left(t\right)$.

In the case of the different examples of phasor expressions studied previously,

(i) the continuous phasor of ungated PSEDs with single-exponential IRF [Eq. (73)],
(ii) the continuous phasor of square-gated PSEDs with Dirac IRF [Eq. (75)],
(iii) the continuous phasor of square-gated PSEDs with single-exponential IRF [Eq. (78)],
(iv) the discrete phasor of ungated PSEDs with Dirac IRF [Eq. (99)],
(v) the discrete phasor of ungated PSEDs with single-exponential IRF [Eq. (101)], and
(vi) the discrete phasor of square-gated PSEDs with Dirac IRF [Eq. (103)],

it is still possible to define a modulus *m* and phase *φ* of the calculated continuous or discrete phasor *z* = *me*^*iφ*^, where $m=m\left(\tau \right)$ and $\varphi =\varphi \left(\tau \right)$ are different functions of *τ* than those of Eq. (149), and the lifetimes *τ*_*φ*_ and *τ*_*m*_ defined as

$$\left\{\begin{array}{c} \tau_{\varphi }=\varphi^{-1}\left(\arg\left(z\right)\right),\\ \tau_{m}=m^{-1}\left(\left|z\right|\right) \end{array}\right.$$ (152)

are not given by Eq. (150) anymore because the phasor expression is different from $\zeta_{f}\left(\tau \right)$ [Eq. (70)].

It is possible to obtain analytical formulas for the phasor modulus and phase in the continuous square-gated decay case and the discrete ungated decay case. However, in general, only implicit formulas can be obtained in the discrete square-gated decay case due to the complexity of Eq. (103), although, in a few special cases, analytical formulas can be obtained.

We will look at these cases in Secs. VII B–VII C.

### B. Phase and modulus lifetime of continuous phasors

#### 1. Phase and modulus lifetime of ungated PSEDs in the presence of a single-exponential IRF

Equation (73) for the phasor in this case can be rewritten as

$$\left\{\begin{array}{c} z\left[\Lambda_{\tau ,T}\right]=m\left(\tau \right)e^{i\varphi \left(\tau \right)},\\ m\left(\tau \right)=\frac{1}{\sqrt{1+{\left(2\pi f\tau_{\times }\right)}^{2}}}\frac{1}{\sqrt{1+{\left(2\pi f\tau \right)}^{2}}},\\ \varphi \left(\tau \right)=\tan^{-1}\left(2\pi f\tau_{\times }\right)+\tan^{-1}\left(2\pi f\tau \right), \end{array}\right.$$ (153)

from which a phase and modulus lifetime can be defined by

$$\left\{\begin{array}{c} \tau_{\varphi }=\tan\left(\varphi -\varphi_{\times }\right),\;\\ \tau_{m}=\frac{1}{2\pi f}\sqrt{{\left(m_{\times }/m\right)}^{2}-1}, \end{array}\right.$$ (154)

where angle *φ*_×_ and modulus *m*_×_ were defined in Eq. (74). These equations are the same as Eq. (150) after rotation of the phasor by *φ*_×_ and dilation by a factor 1/*m*_×_. Obviously, for this calculation to be possible, *m*_×_ and *φ*_×_, the modulus and phase of the excitation pulse, need to be known. A simpler alternative is provided by phasor calibration, as discussed in Sec. VIII.

#### 2. Phase and modulus lifetime of continuous square-gated PSEDs with Dirac IRF

Writing Eq. (75) for the continuous phasor of an ungated PSED with Dirac IRF as

$$z_{\left[W\right]}\left[\Lambda_{\tau ,T}\right]=m\left(\tau \right)e^{i\varphi \left(\tau \right)},$$ (155)

we obtain the following equations for the phase and modulus lifetimes of square-gated PSEDs:

$$\left\{\begin{array}{c} \tau_{\varphi }=\frac{1}{2\pi f}\tan\left(\varphi +\varphi_{W}\right),\\ \tau_{m}=\frac{1}{2\pi f}\sqrt{{\left(M_{W}/m\right)}^{2}-1}, \end{array}\right.$$ (156)

where *φ*_*W*_ and *M*_*W*_ are defined in Eq. (75). These equations simply express the fact that $L_{\left[W\right]}$ is a rotated and dilated version of $L_{\infty }$, and thus, the modulus and phase of the phasor need to be, respectively, rescaled and corrected before using the formulas valid for $L_{\infty }$ [Eq. (150)].

#### 3. Phase and modulus lifetime of continuous square-gated PSEDs with single-exponential IRF

Writing Eq. (78) for the continuous phasor of a square-gated PSED with single-exponential IRF as

$$z_{\left[W\right]}\left[\Psi_{\tau ,\tau_{\times },T}\right]=m\left(\tau \right)e^{i\varphi \left(\tau \right)},$$ (157)

we obtain the following equations for the phase and modulus lifetimes:

$$\left\{\begin{array}{c} \tau_{\varphi }=\frac{1}{2\pi f}\tan\left(\varphi +\varphi_{W}-\varphi_{\times }\right),\\ \tau_{m}=\frac{1}{2\pi f}\sqrt{{\left(m_{\times }M_{W}/m\right)}^{2}-1}. \end{array}\right.$$ (158)

### C. Phase and modulus lifetime of discrete phasors

#### 1. Phase and modulus lifetime of discrete ungated PSEDs with Dirac IRF

Writing $z_{N}\left[\Lambda_{\tau ,T}\right]$ in Eq. (99) as

$$z_{N}\left[\Lambda_{\tau ,T}\right]=m\left(\tau \right)e^{i\varphi \left(\tau \right)},$$ (159)

we obtain the following equations for the phase and modulus lifetimes of discrete ungated PSEDs with Dirac IRF, after some straightforward algebra:

$$\left.\begin{array}{c} m\leq 1\Rightarrow \tau_{m}=\tau_{m}^{-},\\ 1<m\leq {\left|\cos\frac{\alpha }{2}\right|}^{-1}\Rightarrow \tau_{m}=\tau_{m}^{+} \end{array}\right\}\to \left\{\begin{array}{c} \tau_{\varphi }=\frac{\theta }{\ln\left[\cos\alpha \left(1+\frac{\tan\alpha }{\tan\varphi }\right)\right]},\\ \tau_{m}^{\pm }=\frac{\theta }{\ln\left(\frac{1-m^{2}}{{\left(\sqrt{1-m^{2}\cos^{2}\frac{\alpha }{2}}\pm m\left|\sin\frac{\alpha }{2}\right|\right)}^{2}}\right)}, \end{array}\right.$$ (160)

where we have used the notation *α* = 2*πf θ* introduced in Eq. (99). Note that the term ${\left|\cos\frac{\alpha }{2}\right|}^{-1}$ in Eq. (160) is the diameter of the circle of which $L_{N}$ is a part, and therefore, the condition expressed in Eq. (160) simply states that the phasor needs to be inside $L_{N}$ for the modulus lifetime to be defined, as expected.

Note also that there is no simple connection between these expressions and those valid in the simpler case of ungated decays [Eq. (150)].

#### 2. Phase and modulus lifetime of discrete ungated PSEDs with single-exponential IRF

Writing $z_{N}\left[\Psi_{\tau ,\tau_{\times },T}\right]$ in Eq. (101) as

$$z_{N}\left[\Psi_{\tau ,\tau_{\times },T}\right]=m\left(\tau \right)e^{i\varphi \left(\tau \right)},$$ (161)

we obtain the same equations as those obtained for the phase and modulus lifetimes of discrete ungated PSEDs with Dirac IRF [Eq. (160)], with the simple replacements

$$\left\{\begin{array}{c} m\mapsto \frac{m}{\left(\frac{x_{\times }\sin\alpha }{1-x_{\times }\cos\alpha }\right)},\\ \varphi \mapsto \varphi -\alpha -\tan^{-1}\left(\frac{x_{\times }\sin\alpha }{1-x_{\times }\cos\alpha }\right). \end{array}\right.$$ (162)

#### 3. Phase and modulus lifetime of discrete square-gated PSEDs with Dirac IRF

**FIG. 11.**
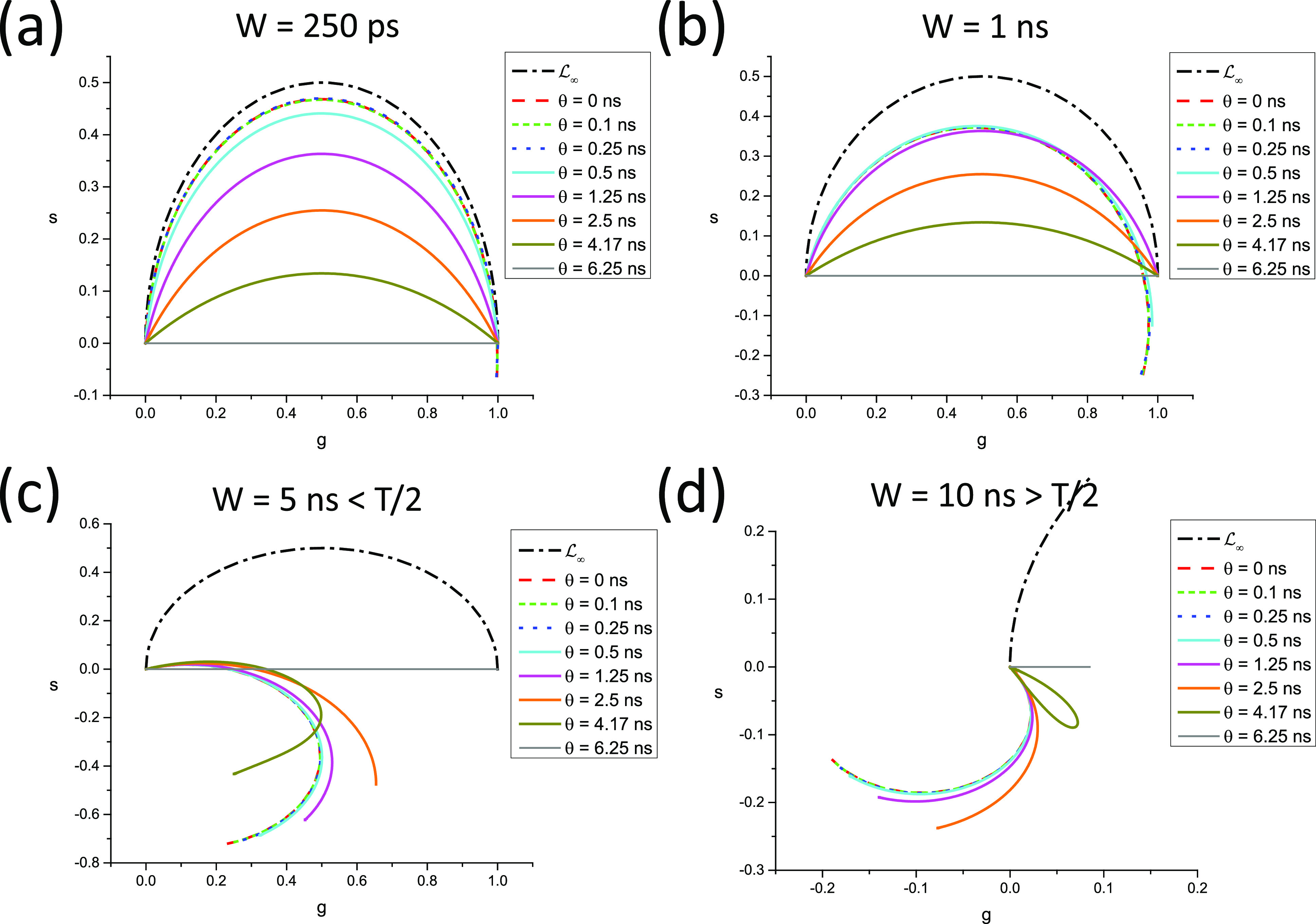
Locus of the discrete phasors of periodic square-gated single-exponential decays, $L_{N\left[W\right]}$, for different *N* and *W*. The curves were calculated for a laser period *T* = 12.5 ns and the corresponding first harmonic frequency f_1_ = 80 MHz. Each panel corresponds to a different gate width, each curve corresponding to a different gate step choice *θ* (the number of gates is *N* = *T*/*θ*). (a) *W* = 250 ps, (b) *W* = 1 ns, (c) *W* = 5 ns, (d) *W* = 10 ns. Note, in particular, the locus of phasors for *N* = 2 (*θ* = 6.25 ns), which covers the whole [0,1] segment, except when *W* > *T*/2. Of particular interest is the case *N* = 3 and *W* > *T*/2, for which the $L_{N\left[W\right]}$ forms a closed loop.

As mentioned previously, Eq. (103) for *z* does not, in general, lead to any simple relation between the phase and modulus of *z* and the lifetime *τ*. In fact, as is visible in Fig. 11, for some choices of gate width *W* and gate step *θ*, different PSEDs can be associated with the same modulus, showing that, in some cases, looking for an unambiguous modulus lifetime is impossible using the implicit formula

$$\left|z_{N\left[W\right]}\left[\Lambda_{\tau ,T}\right]\right|=m\left(\tau \right).$$ (163)

Fortunately, this is not the case for the phase *φ*, which appears to be uniquely defined for *τ* ≥ 0. The implicit relation between the phase $\varphi \left(\tau \right)$ and lifetime *τ* is given by

$$\frac{Im\left(z_{N\left[W\right]}\left[\Lambda_{\tau ,T}\right]\right)}{Re\left(z_{N\left[W\right]}\left[\Lambda_{\tau ,T}\right]\right)}=\tan\varphi \left(\tau \right).$$ (164)

Numerical solutions of these equations can be obtained using standard iterative procedures and are implemented in the *Phasor Explorer* software provided with this article (see the supplementary material and Data Availability sections).

Note that all the results above are only valid in the case where there is no decay offset (the laser pulse corresponds to *t*_0_ = 0 in the decay recording coordinates) and the decay is not truncated (*T* = *Nθ*). In practice, the decay might well be complete, but unless care is taken to circularly shift the decay such that time 0 in the recording corresponds to the IRF peak, the locus of phasors of PSEDs will be different from one of the tractable situations described above, making it impractical to obtain modified equations for the phase and modulus lifetime.

While this seems to imply that the prospect of interpreting phasor data in terms of phase (or modulus) lifetime in the most general case is compromised, it turns out that a simple approach based on the concept of phasor calibration can provide useful quantitative results in most practical situations, as discussed next.

## VIII. PHASOR CALIBRATION AND PSEUDO-CALIBRATION

Phasor calibration is a central concept in *continuous* phasor analysis, abstracting all experimental details into a simple algebraic operation, in order to bring back the phasors of PSEDs to the universal semicircle $L_{\infty }$. In Sec. VIII A, we first qualitatively discuss how this is modified in the non-standard cases examined in this article, before briefly reviewing the case of continuous phasors (Sec. VIII B) and examining discrete phasors quantitatively (Sec. VIII C).

### A. Reference single-exponential phasor loci

**FIG. 12.**
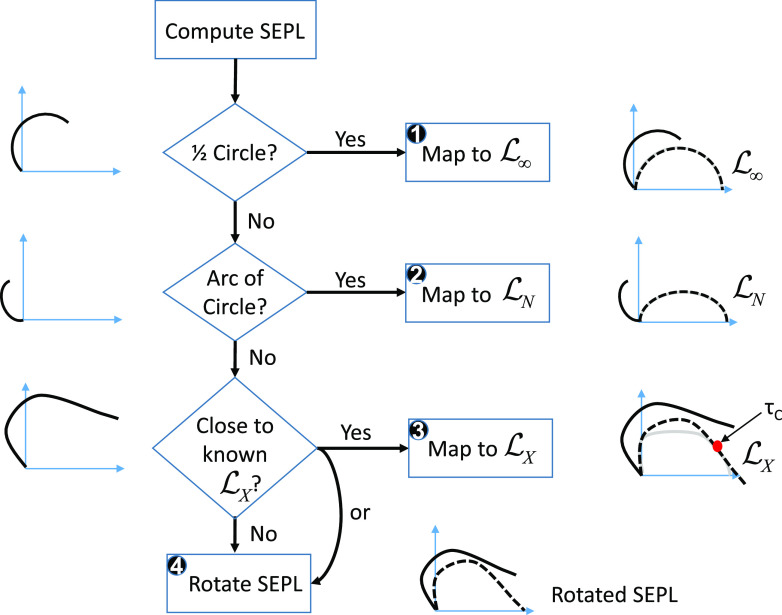
Phasor calibration workflow. The Single-Exponential Phasor Locus (SEPL) can be computed if the IRF shape (including the gate shape effect) is known. The resulting curve (shown on the left side of the figure) can be compared to the simple cases studied here. Case 1: if the SEPL is a rotated, scaled half-circle, it is natural to use the universal semicircle, $L_{\infty }$, as reference. In that case, the calibrated phasors are mapped exactly to the corresponding phasors on the universal semicircle. Case 2: if the SEPL is a rotated, scaled arc of circle, it is natural to use $L_{N}$ as reference. In that case, the calibrated phasors are mapped exactly to the corresponding phasors on $L_{N}$. Case 3: if the SEPL, after rotation and scaling, is identical to (respectively, close to) a simple SEPL (generically referred to as $L_{X}$), it is natural to it as a reference. In that case, the calibrated phasors are mapped exactly to (respectively, close to) the corresponding phasors on $L_{X}$. Case 4: if there is no good match of the rotated/rescaled SEPL to any known reference SEPL, it is simpler to rotate and scale the SEPL so that the phasor of a PSED with zero lifetime is mapped to 1.

The results of the Secs. III–VI have shown that different data recording situations may result in different loci of the phasor of PSEDs (a curve dubbed SEPL) in the phasor plot. Does this mean that phasor data need to interpreted differently each time the SEPL changes, i.e., each time some modification of the experimental conditions happens? In the case of continuous phasors of complete decays, the answer is no, provided that *phasor calibration* is used, as discussed in Subsection VIII B. We will first qualitatively discuss this familiar situation and contrast it with non-standard cases discussed in Secs. III–VI, in order to extend this notion to these more complex cases. To help with the discussion, the different cases addressed in this section are schematically illustrated in Fig. 12.

As discussed in Secs. III and IV, for *continuous phasors of non-truncated decays*, the effects of the excitation pulse profile, instrument integration (gating), or offset can all be described as convolution products of various decay-independent functions with the “pure” luminescence decays of interest (i.e., the signal emitted by the sample upon excitation by a Dirac comb). Due to the *continuous phasor convolution rule* [Sec. III B 1, Eq. (69)], this means that the resulting phasors are merely multiplied by a constant complex number, compared to the ideal situation where the IRF is a Dirac function (and therefore, no detector gating or binning takes place). In other words, the experimental continuous phasors only differ by a dilation and a rotation from the phasors of the pure single-exponential decays, whatever the nature of the excitation function and electronic response function (including gating and offset) are. The corresponding SEPL is thus a rescaled and rotated version of the canonical $L_{\infty }$ obtained for continuous, offset-free, ungated PSEDs.

In these simple cases, an opposite rotation and dilation will bring the experimental *SEPL* back to the reference $L_{\infty }$, as illustrated in Fig. 12(1). Data interpretation and analysis are thus simplified, being performed within the same familiar framework, where the phasor of a zero lifetime PSED is equal to 1, the phasor of an infinite lifetime PSED is equal to 0, and the phasor of PSEDs with finite lifetimes is located at predictable locations on the universal semi-circle $L_{\infty }$.

In all other cases, however, the difference is more profound, such as in the case of discrete phasors (discussed in Secs. III C and IV C 1 and IV C 2) or for continuous phasors of truncated decays (Sec. V). Indeed, the locus of discrete phasors of *ungated*, *offset-free* PSEDs with Dirac IRF, $L_{N}$ [Eq. (99)], is a circular *arc* instead of a full semicircle, whose radius *r* depends on the number of gates *N* according to Eqs. (99) and (100). In the presence of a single-exponential IRF [Eq. (101)] or an IRF offset *t*_0_ [Eq. (131)], the corresponding SEPL is still a circular arc, but the arc is rotated by an angle that depends in a non-trivial manner on the IRF or its offset and, additionally, dilated in the single-exponential IRF case. In those cases, it would seem desirable to use $L_{N}$ as the reference SEPL, since trying to map those SEPLs back to $L_{\infty }$ (a half-circle) by inverse rotation and dilation will clearly not succeed, as illustrated in Fig. 12(2). The advantage of such a remapping to $L_{N}$ would be that, in all these cases, the phasor of a PSED with zero lifetime would always be located at 1 and the phasor of a PSED with infinite lifetime would be located at 0, with the phasor of PSEDs with intermediate lifetimes in-between on the $L_{N}$ at predictable locations.

The fact that things are otherwise more complex is obvious because, for an arbitrary IRF, the discrete phasor of an ungated PSED does not verify any weak rule for the discrete phasors of convolution products.

In the case of *gated* PSEDs, the situation is obscured by the fact that the analytical expression for the phasor is relatively complex, even in the simple case of a *square gate* and a Dirac IRF [Eq. (103)]. In some cases, however, the corresponding SEPL, $L_{N\left[W\right]}$, is “close to” a circular arc ($L_{N}$) as can be seen in Fig. 11, which suggests once again that, in favorable situations, it might be possible to bring the SEPL “close to” a familiar curve ($L_{N}$ or $L_{\infty }$) by inverse rotation and dilation, in order to fall back “approximately” to a familiar situation, as illustrated in Fig. 12(3).

In some other cases, however, some examples of which can be seen in Fig. 11, even such an “approximate mapping” is impossible because the shape of the *SEPL* departs too much from an arc of circle. In these cases, it will be up to the practitioner to decide whether to try and remap the SEPL “partially” to one of the reference SEPLs identified so far ($L_{N}$ or $L_{\infty }$) or instead use a rotated/dilated $L_{N\left[W\right]}$ as reference SEPL or even a rotated/dilated version of the actual SEPL bringing a specific phasor to a particular point in the complex plane (for instance, the phasor of the PSED with zero lifetime to 1), as illustrated in Fig. 12(4).

The remainder of this section will clarify and examine the validity and usefulness of these general statements in the continuous and discrete cases.

### B. Calibration of continuous phasor of periodic decays

According to the continuous phasor convolution rule [Eq. (69)], the continuous phasor of an arbitrary *T*-periodic signal, $S_{T}\left(t\right)=I_{T}\underset{T}{*}F_{0,T}\left(t\right)$, is the product of two phasors: that of the IRF used to acquire the signal and that of the decay obtained (hypothetically) with a Dirac excitation only,

$$z\left[S_{T}\right]=z\left[I_{T}\right]z\left[F_{0,T}\right].$$ (165)

To keep the discussion general, we do not specify the *T*-periodic instrument response function $I_{T}\left(t\right)$, which could be characterized by an arbitrary gate profile $\Gamma_{s,W}\left(t\right)$ and offset *t*_0_. This is of course true for a PSED, for which the phasor reads

$$z\left[I_{T}\underset{T}{*}\Lambda_{\tau ,T}\right]=z\left[I_{T}\right]\zeta_{f}\left(\tau \right).$$ (166)

Both equations use the same IRF phasor $z\left[I_{T}\right]$, which can therefore be computed using any reference sample such as one characterized by a single-exponential decay with lifetime *τ*_*C*_. Equation (166) for that reference yields

$$z\left[I_{T}\right]=\frac{z\left[I_{T}\underset{T}{*}\Lambda_{\tau_{C},T}\right]}{\zeta_{f}\left(\tau_{C}\right)}.$$ (167)

In that equation, $z\left[I_{T}\underset{T}{*}\Lambda_{\tau_{C},T}\right]$ is the *calibration phasor* or phasor of the *reference decay before calibration* obtained from Eq. (63) (a quantity that can be computed from the data) and $\zeta_{f}\left(\tau_{C}\right)$ is the *calibrated phasor of the reference sample*, given by the simple analytical formula of Eq. (70). Their ratio [Eq. (167)] or *calibration factor* is equal to the phasor of the IRF.

This relation is true for any reference lifetime *τ*_*C*_, including *τ*_*C*_ = 0, for which $\zeta_{f}\left(0\right)=1$, yielding

$$z\left[I_{T}\right]=z\left[I_{T}\underset{T}{*}\Lambda_{0,T}\right].$$ (168)

Equation (168) might not always be very useful in practice as it may not be simple to measure the decay of a sample of lifetime 0 (or close enough to 0), i.e., the IRF.

In any case, with the help of such a *calibration factor*, it is possible to obtain the *calibrated phasor* $\tilde{z}\left[S_{T}\right]$ of any measured decay $S_{T}\left(t\right)$ as

$$\tilde{z}\left[S_{T}\right]≜\frac{z\left[S_{T}\right]}{z\left[I_{T}\right]}=z\left[F_{0,T}\right].$$ (169)

We will use a tilde sign above the phasor symbol ($\tilde{z}$) to indicate a calibrated phasor in the remainder of this article. If the calibration phasor is correct (i.e., it is acquired with the same IRF as the samples of interest) and the reference lifetime is accurately known, the calibrated phasor computed by the formula on the right of the ≜ symbol in Eq. (169) is identical to that of the underlying decay hypothetically excited by a Dirac comb, $z\left[F_{0,T}\right]$.

While the calibration phasor is intended to be computed based on experimental data, it can be computed analytically in the simple cases studied before. As an example, for a *T*-periodic single-exponential excitation function with time constant *τ*_×_, a square gate of width *W*, and offset *t*_0_, the calibration phasor $z\left[I_{T}\right]$ is formally given by [Eq. (129)]

$$\begin{array}{ll} z\left[I_{T,W|t_{0}}\right] & =M_{W}e^{-i\varphi_{W}}\zeta_{f}\left(\tau_{\times }\right)e^{i2\pi ft_{0}}\\ & =\frac{\sin\left(\pi fW\right)/\pi fW}{1-i2\pi f\tau_{\times }}e^{i\left(2\pi ft_{0}-\pi fW\right)}. \end{array}$$ (170)

A comparison of the analytical result of Eq. (170) with the numerical result of Eq. (167) might be used, for instance, to estimate the width *W* of a square gate or the IRF offset *t*_0_.

In the case of truncated decays, however, the continuous phasor convolution rule does not apply in general (see Sec. V). Therefore, phasor calibration using a reference PSED as described above (that is, by division by a constant term) will only be useful for PSEDs and only in the case *f* = *n*/*D*, in the absence of gating, and for an instantaneous instrument response function [see Sec. V, Eq. (141)]. In this case, phasor calibration using a PSED reference will bring phasors of PSEDs back to the universal circle $L_{\infty }$, but the calibrated phasors of other arbitrary decays $S_{T}\left(t\right)=I_{T}\underset{T}{*}F_{0,T}\left(t\right)$ will not, in general, be identical to the phasor of $F_{0,T}\left(t\right)$, the original *T*-periodic emission due to a Dirac comb excitation.

In all other cases, *f* ≠ *n*/*D*, *ad hoc* choices will have to be made regarding which phasor calibration to apply (if any) to bring, say, part of the calibrated SEPL “close to” a region of $L_{\infty }$ within which minor differences between some characteristics of the decays (for instance, the lifetime and the phase lifetime extracted using $L_{\infty }$) will exist. We will examine an example of such a situation in Sec. IX B.

### C. Calibration of discrete phasor of periodic decays

In the case of decay functions sampled at a finite number of time points, the continuous phasor is replaced by a discrete phasor [Eq. (92)] and the corresponding discrete phasor convolution rule states that, in general (see Sec. III C 2) [Eq. (97)],

$$z_{N}\left[f_{T}\underset{T}{*}g_{T}\right]\neq z_{N}\left[f_{T}\right]z_{N}\left[g_{T}\right].$$ (171)

When *f*_*T*_ is the IRF, $I_{T}\left(t\right)$, (gated or ungated, with or without offset) and *g*_*T*_ is the *T*-periodic decay, $F_{0,T}\left(t\right)$, resulting from the hypothetical excitation of a sample with a Dirac comb, Eq. (171) says that the discrete case analog of the continuous phasor calibration, i.e., division of the calculated (gated or ungated) phasor by the IRF phasor [Eq. (169)], will *not* provide any direct useful information (in particular, it will not provide $z_{N}\left[F_{0,T}\right]$). We shall examine some special cases in Subsections VIII C 2–VIII C 3.

However, the situation may sometimes be more favorable, and a weak discrete phasor convolution rule applies. In this case, a similar form of phasor calibration as in the continuous case can be implemented, as we shall discuss first.

#### 1. Weak discrete phasor convolution rule cases

In some special cases, the above-mentioned inequality is replaced by a *weak* discrete phasor convolution rule valid for some families of functions only [Eq. (98)],

$$z_{N}\left[f_{T}\underset{T}{*}g_{T}\right]=\kappa \;z_{N}\left[f_{T}\right]z_{N}\left[g_{T}\right],$$ (172)

where *κ* is a constant.

In cases where Eq. (172) applies to the convolution of PSEDs and the instrument response function *I*_*T*_,

$$z_{N}\left[I_{T}\underset{T}{*}\Lambda_{\tau ,T}\right]=\kappa z_{N}\left[I_{T}\right]z_{N}\left[\Lambda_{\tau ,T}\right],$$ (173)

the following modified discrete phasor calibration equation can be used:

$${\tilde{z}}_{N}\left[I_{T}\underset{T}{*}\Lambda_{\tau ,T}\right]≜\frac{z_{N}\left[I_{T}\underset{T}{*}\Lambda_{\tau ,T}\right]}{\kappa z_{N}\left[I_{T}\right]}=z_{N}\left[\Lambda_{\tau ,T}\right].$$ (174)

As in the continuous phasor case, the *calibration factor* $\kappa z_{N}\left[I_{T}\right]$ can be obtained with the help of a single-exponential decay with known lifetime *τ*_*C*_ by

$$\kappa z_{N}\left[I_{T}\right]=\frac{z_{N}\left[I_{T}\underset{T}{*}\Lambda_{\tau_{C},T}\right]}{z_{N}\left[\Lambda_{\tau_{C},T}\right]},$$ (175)

where $z_{N}\left[I_{T}\underset{T}{*}\Lambda_{\tau_{C},T}\right]$ is the phasor of the *reference decay before calibration* obtained from Eq. (88), using the measured gated values $S_{T}\left(t_{p}\right)$ (*p* = 1, …, *N*). $z_{N}\left[\Lambda_{\tau_{C},T}\right]$ is given by the same Eq. (88) computed using the analytical formula for $S_{T}\left(t\right)=\Lambda_{\tau_{C},T}\left(t\right)$ and takes the simple form $\zeta_{f,N}\left(\tau \right)$ given by Eq. (99). As in the continuous case, the calibration factor could in principle be obtained with a reference sample of lifetime 0 (i.e., the IRF) for which $z_{N}\left[\Lambda_{0,T}\right]=1$,

$$\kappa z_{N}\left[I_{T}\right]=z_{N}\left[I_{T}\underset{T}{*}\Lambda_{0,T}\right],$$ (176)

although this could be a challenging measurement to perform. It is generally easier to use a sample with known finite lifetime *τ*_*C*_.

As we have seen in Sec. III C, a relation such as Eq. (173) can be obtained only in a few special cases such as for ungated decays with single-exponential IRF [Eq. (102)] and square-gated decays with Dirac IRF [when the gate width is proportional to gate step, Eq. (107)].

In those cases, since the discrete phasor $\zeta_{f,N}\left(\tau \right)$ given by Eq. (99) is located on a circular arc, $L_{N}$, described in Sec. III C 3, Eq. (174) states that the calibrated discrete phasor of such IRF-convolved PSEDs are mapped back to $L_{N}$, which therefore takes the role that $L_{\infty }$ played for continuous phasors.

The usefulness of such calibration is not limited to PSEDs. The weak discrete phasor convolution rule for PSEDs [Eq. (174)] results in a similar relation for any arbitrary recorded *T*-periodic decay $S_{T}\left(t\right)$. Indeed, we can introduce the ${\left\|\right\|}_{N}$-normalized recorded decay $\sigma_{T}\left(t\right)$,

$$\left\{\begin{array}{c} \sigma_{T}\left(t\right)=\frac{S_{T}\left(t\right)}{{\left\|S_{T}\right\|}_{N}}=\overset{\infty }{\underset{0}{\int }}d\tau \mu_{0}\left(\tau \right)\frac{I_{T}\underset{T}{*}\Lambda_{\tau ,T}\left(t\right)}{{\left\|I_{T}\underset{T}{*}\Lambda_{\tau ,T}\right\|}_{N}},\\ \overset{\infty }{\underset{0}{\int }}d\tau \mu_{0}\left(\tau \right)=1, \end{array}\right.$$ (177)

where $\mu_{0}\left(\tau \right)$ is a weight function in the ${\left\|\right\|}_{N}$-normalized base ${\left\{I_{T}\underset{T}{*}\Lambda_{\tau ,T}\left(t\right)/{\left\|I_{T}\underset{T}{*}\Lambda_{\tau ,T}\right\|}_{N}\right\}}_{\tau \geq 0}$. It follows from Eq. (177) that

$$\begin{array}{ll} z_{N}\left[S_{T}\right] & =z_{N}\left[\sigma_{T}\right]=\overset{\infty }{\underset{0}{\int }}d\tau \mu_{0}\left(\tau \right)\frac{{\left\|I_{T}\underset{T}{*}\Lambda_{\tau ,T}\left(t_{p}\right)e^{i2\pi ft_{p}}\right\|}_{N}}{{\left\|I_{T}\underset{T}{*}\Lambda_{\tau ,T}\right\|}_{N}}\\ & =\overset{\infty }{\underset{0}{\int }}d\tau \mu_{0}\left(\tau \right)z_{N}\left[I_{T}\underset{T}{*}\Lambda_{\tau ,T}\right]\\ & =\kappa z_{N}\left[I_{T}\right]\overset{\infty }{\underset{0}{\int }}d\tau \mu_{0}\left(\tau \right)\zeta_{f,N}\left(\tau \right), \end{array}$$ (178)

where we have used the definition of $z_{N}\left[\Lambda_{\tau ,T}\right]$ given in Eq. (99).

In other words, the weak discrete phasor convolution rule allows the phasor of an arbitrary recorded decay $S_{T}\left(t\right)$, expressed in the basis of ${\left\|\right\|}_{N}$-normalized IRF-convolved PSEDs, to be written as the product of $\kappa z_{N}\left[I_{T}\right]$ with the phasor of the same weighted sum of PSEDs, ${\left\{\Lambda_{\tau ,T}\left(t\right)\right\}}_{\tau >0}$. This also means that, after calibration with the calibration factor given by Eq. (175), the calibrated phasor of $S_{T}\left(t\right)$ [and $\sigma_{T}\left(t\right)$] is given by a similar linear relation,

$${\tilde{z}}_{N}\left[S_{T}\right]={\tilde{z}}_{N}\left[\sigma_{T}\right]=\overset{\infty }{\underset{0}{\int }}d\tau \mu_{0}\left(\tau \right)\zeta_{f,N}\left(\tau \right).$$ (179)

In particular, for a Dirac comb-excited decay equal to a linear combination of exponentials PSEDs,

$$F_{0,T}\left(t\right)=\overset{n}{\underset{i=1}{\sum }}a_{i}\tau_{i}\Lambda_{\tau_{i},T}\left(t\right).$$ (180)

The sum in Eq. (180) can be rewritten in terms of the ${\left\|\right\|}_{N}$-normalized PSEDs, ${\left\{\Lambda_{\tau ,T,N}\left(t\right)\right\}}_{\tau >0}$, using the results of Appendix B.3,

$$\left\{\begin{array}{c} f_{0,T}\left(t\right)=\frac{F_{0,T}\left(t\right)}{{\left\|F_{0,T}\right\|}_{N}}=\overset{n}{\underset{i=1}{\sum }}\mu_{i}\Lambda_{\tau_{i},T,N}\left(t\right),\\ \mu_{i}=\frac{a_{i}}{\left(1-e^{-{\theta /\tau }_{i}}\right)}/\overset{n}{\underset{j=1}{\sum }}\frac{a_{j}}{\left(1-e^{-{\theta /\tau }_{j}}\right)}. \end{array}\right.$$ (181)

This gives the phasor of the IRF-convolved decay $S_{T}\left(t\right)$ as

$$\begin{array}{ll} z_{N}\left[S_{T}\right] & =\kappa z_{N}\left[I_{T}\right]\overset{\infty }{\underset{0}{\int }}d\tau \mu_{0}\left(\tau \right)z_{N}\left[\Lambda_{\tau ,T,N}\right]\\ & =\kappa z_{N}\left[I_{T}\right]\overset{n}{\underset{i=1}{\sum }}\mu_{i}\zeta_{f,N}\left(\tau_{i}\right). \end{array}$$ (182)

After calibration with (i.e., division by) $\kappa z_{N}\left[I_{T}\right]$, the calibrated phasor is thus

$${\tilde{z}}_{N}\left[S_{T}\right]=\overset{n}{\underset{i=1}{\sum }}\mu_{i}z_{N}\left[\Lambda_{\tau_{i},T}\right].$$ (183)

The *calibrated* discrete phasor of a *T*-periodic decay is therefore equal to the same linear combination of the PSED phasors as the normalized “pure decay” (obtained with a Dirac comb IRF) is of the normalized PSEDs [Eq. (181)].

In summary, whenever a weak discrete phasor convolution rule applies for PSEDs and the instrument response function *I*_*T*_, phasor calibration with the calibration factor $\kappa z_{N}\left[I_{T}\right]$ obtained using a reference sample [Eq. (175)] maps any phasor to an easily interpretable phasor.

We shall now look at the two special cases discussed previously.

#### 2. Special case 1: Ungated PSEDs with single-exponential IRF

As seen in Sec. III C 3, the discrete phasor of ungated PSEDs with single-exponential IRF with time constant *τ*_×_ [Eq. (102)] can be rewritten in the form of Eq. (174) with *κ* = *e*^*iα*^ = *e*^*i*2*πfθ*^ since the IRF *I*_*T*_ is equal to the excitation function $\Lambda_{\tau_{\times },T}$,

$$z_{N}\left[\Psi_{\tau ,\tau_{\times },T}\right]=e^{i\alpha }z_{N}\left[I_{T}\right]z_{N}\left[\Lambda_{\tau ,T}\right].$$ (184)

The calibration factor $e^{i\alpha }\;z_{N}\left[I_{T}\right]$ can thus be obtained using any reference PSED with lifetime *τ*_*C*_,

$$e^{i\alpha }\;z_{N}\left[I_{T}\right]=\frac{z_{N}\left[\Psi_{\tau_{C},\tau_{\times },T}\right]}{z_{N}\left[\Lambda_{\tau_{C},T}\right]}=e^{i\alpha }z_{N}\left[\Lambda_{\tau_{\times },T}\right]=e^{i\alpha }\frac{1-x_{\times }}{1-x_{\times }e^{i\alpha }}.$$ (185)

#### 3. Special case 2: Square-gated PSEDs with Dirac IRF and gate width W proportional to the gate step *θ*

As discussed in Sec. III C 4, in the special cases where the gate width *W* is proportional to the gate step *θ*, *W* = *qθ*, the discrete phasor of square-gated PSEDs reads [Eq. (107)]

$$W=q\theta \;\Rightarrow \;z_{N\left[W\right]}\left[\Lambda_{\tau ,T}\right]=e^{i\alpha }\;z_{N}\left[I_{T,W}\right]\;z_{N}\left[\Lambda_{\tau ,T}\right],$$ (186)

where we have used the fact that the gated IRF, *I*_*T*,*W*_, is identical to the gate function ${\bar{\Pi }}_{W,nT}$ in that specific case. This relation is of the form of Eq. (174), with *κ* = *e*^*iα*^ = *e*^*i*2*πfθ*^, and the calibration factor $e^{i\alpha }\;z_{N}\left[I_{T,W}\right]$ is

$$e^{i\alpha }\;z_{N}\left[I_{T,W}\right]=\frac{z_{N\left[W\right]}\left[\Lambda_{\tau_{C},T}\right]}{z_{N}\left[\Lambda_{\tau_{C},T}\right]}=e^{-i\left(q-3\right)\alpha /2}\frac{\sin q\frac{\alpha }{2}}{q\sin\frac{\alpha }{2}},$$ (187)

where the last expression comes from Eq. (B40) in Appendix B.

#### 4. Cases where discrete phasor calibration does not map the SEPL to a known SEPL: Pseudo-calibration

Even in the simple case of a Dirac excitation and a square gate, as soon as the gate width is not proportional to the gate step, the discrete phasor of square-gated PSEDs takes a complex form [Eq. (103)], and no weak discrete phasor convolution rule applies.

Another way of describing this property is to note that the loci of the discrete phasor of square-gated PSEDs describe a SEPL ($L_{N\left[W\right]}$) distinct from an arc of circle [see Fig. 5(a)] and its discussion in Sec. III C 6) and cannot be mapped to another, simpler SEPL (for instance, $L_{N}$ or $L_{\infty }$, which are both arcs of circle).

Of course, this does not mean that the discrete phasors cannot be “pseudo-calibrated” by choosing a particular reference PSED with lifetime *τ*_*C*_ (or any other decay with known analytical form) and defining a *pseudo-calibrated phasor* ${\tilde{z}}_{N}\left[S_{T}\right]$ by an equation of the form of Eq. (174),

$${\tilde{z}}_{N}\left[S_{T}\right]≜\frac{z_{N}\left[S_{T}\right]}{z_{N}\left[{I_{T}}_{T}^{*}\Lambda_{\tau_{C,}T}\right]/\zeta_{f,N}\left(\tau_{C}\right)}\neq z_{N}\left[F_{0,T}\right].$$ (188)

However, as indicated by the ≠ symbol, the resulting pseudo-calibrated phasor of the measured decay *S*_*T*_ will in general be different from that of the underlying decay *f*_0,*T*_ obtained in the presence of a Dirac excitation function. We will note the corresponding pseudo-calibrated SEPL with a tilde as well, $\tilde{L}$.

In some cases, however, the SEPL does not differ much from an arc of circle, and therefore, such an attempt to map it back to another one (for instance, $L_{N}$ or $L_{\infty }$) might be justified if it simplifies phasor data interpretation. The Phasor Explorer software discussed in Appendix F allows fitting a calculated SEPL to an arc of circle, thereby enabling us to quantify the similarity of the SEPL to a circle (for instance, by the mean square error of the fit or a graphical comparison of both).

More generally, the SEPL might be reasonably close to an arc of circle for a range of lifetimes of interest $\left[\tau_{\min},\tau_{\max}\right]$, which means that using a relation such as Eq. (188), where the reference lifetime *τ*_*C*_ is chosen in the interval $\left[\tau_{\min},\tau_{\max}\right]$, will bring the discrete phasors of PSEDs with lifetime in this interval close to their “standard” locations on the chosen target SEPL (for instance, $L_{N}$ or $L_{\infty }$). For lifetimes outside that range, the pseudo-calibrated discrete phasors of PSEDs (and obviously of arbitrary decays) will depart from the discrete canonical discrete phasors $\zeta_{f,N}\left(\tau \right)$.

Because of the variety of experimental situations encountered in terms of excitation function, gate shape, width, and separation, it is impossible to provide quantitative or even qualitative rules to determine when such a pseudo-calibration may be useful or which reference lifetime might be appropriate. However, a simple metric reporting on the proximity (or lack thereof) of the resulting tentative mapping consists in comparing the phase lifetime of pseudo-calibrated PSED phasors (calculated using the appropriate formula for the phase lifetime, see Sec. VII) to their known PSED lifetimes. Small differences will be indicative of a reasonable calibration, while significant departure will indicate a poor approximation. In general, however, the best strategy is described in Sec. VIII C 5.

#### 5. Pseudo-calibration of the discrete phasor in the general case

Whenever no such relation as Eq. (173) exists, the locus of PSED phasors (SEPL) is a curve that, in general, will be complex (i.e., not an arc of circle) and dilated/rotated about the origin, in the sense that the phasor of the PSED with lifetime 0 will be different from 1 and its norm will generally be different from 1 too. However, except in the case of truncated decays discussed in Sec. V, for which the phasor of a PSED with infinite lifetime does not coincide with the origin when the phasor harmonic is not well-matched with the record duration *D*, in all other cases, the SEPL is anchored at the origin and it is possible to rotate and dilate it such that the phasor of a PSED with zero lifetime is mapped to *z* = 1 [as illustrated in Fig. 12(4)], using the following definition of the *pseudo-calibrated phasor*:

$${\tilde{z}}_{N}\left[I_{T}\underset{T}{*}\Lambda_{\tau ,T}\right]≜\frac{z_{N}\left[I_{T}\underset{T}{*}\Lambda_{\tau ,T}\right]}{z_{N}\left[I_{T}\underset{T}{*}\Lambda_{0,T}\right]}.$$ (189)

The pseudo-calibration factor used in this equation, $z_{N}\left[I_{T}\underset{T}{*}\Lambda_{0,T}\right]$, corresponds to the phasor of a PSED with 0 lifetime, i.e., the IRF. Although it might not always be simple to acquire such an IRF signal in the same conditions as other samples of interest, there is a strong motivation in attempting to do so: the uncalibrated phasor of the IRF can then be used as a pseudo-calibration factor [Eq. (189)] and the IRF decay itself, $I_{T}\underset{T}{*}\Lambda_{0,T}\left(t_{p}\right)=I_{T}\left(t_{p}\right),\;p=1,\ldots ,N$, can be used to compute the SEPL for this experiment, at least approximately (see Appendix E for details), providing a convenient reference curve for phasor interpretation.

After this pseudo-calibration operation, the pseudo-calibrated SEPL, $\tilde{L}$, will be a curve anchored at both points 0 (locus of pseudo-calibrated phasors with infinite lifetime) and 1 (locus of pseudo-calibrated phasors with zero lifetime) and can be labeled with indicators marking the location of PSEDs with specific lifetimes (e.g., 0.1 ns–0.9 ns, 1 ns–9 ns, etc., as shown, for instance, in Fig. 10).

## IX. APPLICATION TO EXPERIMENTAL DATA

### A. 4-Gate confocal FLIM

In a pioneering work examining the use of phasor analysis with time-gated fluorescence data, Fereidouni *et al.* used a slightly different formalism than that used here and limited their analysis to the case of adjacent gates (*W* = *θ*).^14^ As we have seen (Sec. III C 4), in this case, the discrete phasor of a square-gated PSED is identical to that of an ungated PSED [Eq. (109)]. The purpose of this section is to connect both works and clarify some differences.

In the theoretical part of Ref. 14, a phasor harmonic *f*_*n*_ = *n*/*D*, *D* = *Nθ* is used, but *D*, the total span of the *N* adjacent gates (width *W* = gate separation *θ*), is not assumed to cover the whole laser period *T* (the decay is truncated, as defined in Sec. V). Indeed, while they define *T* = *Nθ*, in their notation, *T* can be different from the laser period—and in fact is in this and the subsequent sub-section (Sec. IX B). We will replace it with the notation used throughout this article, Eq. (88) and (92), i.e., *D* = *Nθ*, to avoid confusion and reserve the notation *T* for the laser period.

Additionally, the first gate is assumed to start at *t*_1_ = 0 and the gate centers are used as arguments of the complex exponentials in Eq. (92), rather than the gate beginnings, *t*_*p*_,

$$e^{i2\pi t_{p}}\mapsto e^{i2\pi \left(t_{p}+W/2\right)},\;t_{p}=\left(p-1\right)\theta ,\;p\in \left[1,N\right].$$ (190)

Finally, while Ref. 14 deals nominally with square-gated PSEDs, which would call for the use of $\Lambda_{\tau ,T,W}\left(t_{p}\right)$ [defined by Eq. (47)] in Eq. (92), it is instead replaced by the simpler form, appropriate for ungated periodic signals,

$$\Lambda_{\tau ,T,W}\left(t_{p}\right)\underset{W=\theta }{\mapsto }\overset{t_{p}+W}{\underset{t_{p}}{\int }}e^{-t/\tau }dt=\tau \left(1-e^{-\theta /\tau }\right)e^{-t_{p}/\tau }.$$ (191)

While this replacement ignores the *T*-periodicity of the decay, in cases where the time argument *t*_*p*_ is smaller than *T* - *W*, this is an acceptable form [compare Eq. (17) and Eq. (47)]. This clearly requires that *truncated* decays are considered.

As discussed in Sec. III C, using the gate centers rather than their beginning results in a mere rotation of the SEPL calculated using the beginning of the gates (the choice made in this work) by an angle *πfθ*, where *f* is the phasor harmonic.

Using the replacement of Eq. (191) amounts to using Eq. (142) with *t*_1_ = *θ*/2 for the phasor, i.e., the discrete phasor of a truncated ungated PSED with a first gate starting at *t*_1_ = *θ*/2. The result can be rewritten in the case *D* = *Nθ* and *f* = *n*/*D* as

$${\overset{↔}{z}}_{N}\left[\Lambda_{\tau ,D}\right]=\frac{\sinh\theta /2\tau }{\sinh\left(\theta /2\tau -i\alpha /2\right)},$$ (192)

with *α* = 2*πfθ*, which is Eq. (10) of Ref. 14.

**FIG. 13.**
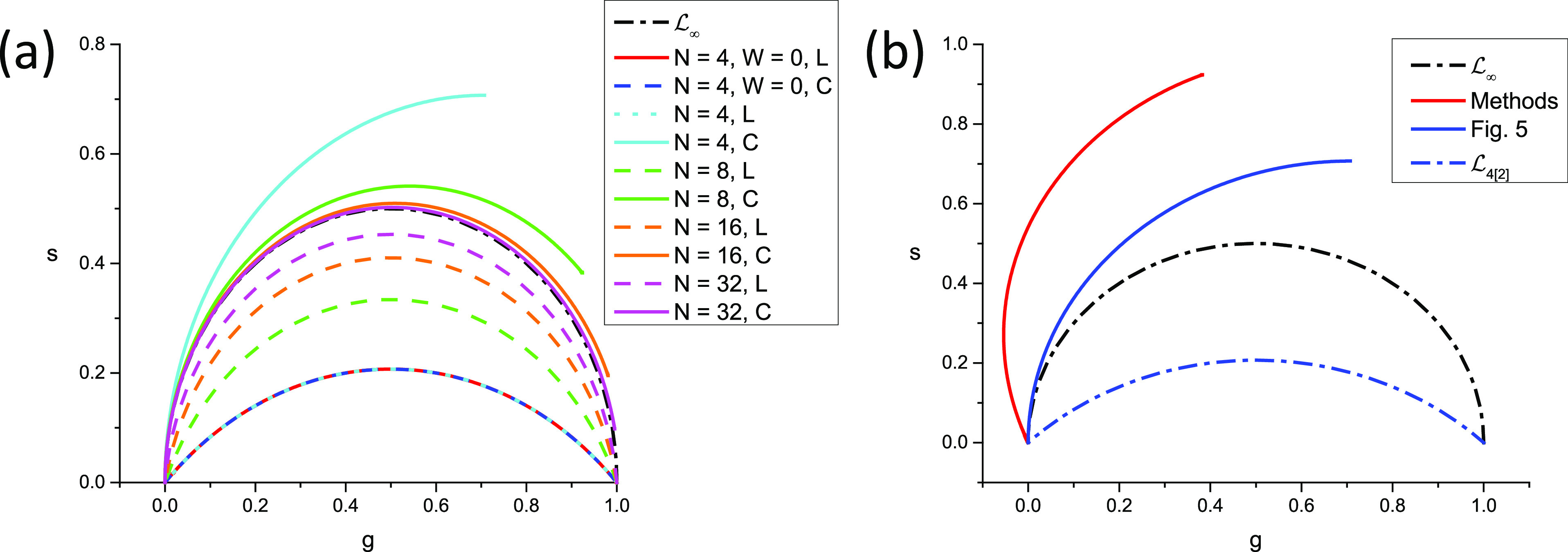
(a) Comparison of $L_{N\left[W\right]}$ computed for *T* = *Nθ*, *W* = *θ*, f = 1/*T* using the argument of the complex exponential terms equal to the beginning of each gate (L, dashed curves) or the center of each gate (C, plain curves). Both differ only by a rotation of *πfθ* = *π*/*N*. Note that when *W* = 0 (instead of *θ*), there is no difference between the two conventions (compare red and dashed blue curves). The plain curves are identical to those presented in Fig. 2 of Ref. 14. (b) $L_{N\left[W\right]}$computed as described in the experimental methods of Ref. 14, and as presented in Fig. 5 of that article [this latter curve is identical to the curve *N* = 4, C of panel (a)]. Also shown is $L_{N\left[W\right]}$ corresponding to *N* = 4 and *W* = 2 ns without offset (dotted-dashed blue curve).

The solid curves in Fig. 13(a), representing the locus of Eq. (192) for different number of gates *N*, are identical to those shown in Fig. 2 of Ref. 14, which assumes *D* = *T*. The $L_{N\left[W\right]}$ calculated using the convention used in this work, that is, with an exponential argument in Eq. (92) equal to *t*_*p*_, the location of the beginning of the *p*^th^ gate rather than its center, are shown as dashed curves on the same graph.

In the experimental section of Ref. 14, the authors used the discrete phasor to study fluorescent samples characterized by distinct lifetimes. The information provided is *T* = 20 ns, *W* = *θ* = 2 ns, *N* = 4 (imposed by the hardware) and that the first gate starts at *t*_1_ = 0.5 ns (= *θ*/4). Consistent with the definition of the phasor harmonic in terms of the total recording span, *f* = 1/*D* = 1/*Nθ* = 125 MHz is chosen. In this case, because the gates do not cover the full laser period, this frequency does not belong to the series of Fourier harmonic frequencies for a *T*-periodic decay (*f* = 50 MHz, 100 MHz, etc.). Using Eq. (143) and accounting for the additional rotation of *πfW* due to the choice of the gate centers as arguments of the complex exponential term (see Sec. III C), we expect

$$z=e^{i2\pi f\left(t_{1}+W/2\right)}z_{N}\left[\Lambda_{\tau ,T}\right]=e^{i\frac{3\pi }{8}}z_{N}\left[\Lambda_{\tau ,T}\right],$$ (193)

where we have used *t*_1_ = *θ*/4, *W* = *θ*, *f* = 1/4*θ*, as defined above. Equation (193) is identical to Eq. (192) but has the advantage to be easier to interpret since the locus of $z_{N}\left[\Lambda_{\tau ,T}\right]$ is an arc of circle, namely, $L_{N}$, and the prefactor amounts to a rotation angle of 3π/8. The corresponding curve is indicated in red in Fig. 13(b). This is a rotated version (by an angle π/8) of the curve obtained for adjacent gates covering the whole period [Fig. 13(b), blue curve], which is also shown in Fig. 13(a) as “*N* = 4, C” (solid light blue). Since the authors reported their calculated experimental phasors as falling on that latter curve, we have to conclude that, in practice, they used the replacement $t_{p}\mapsto t_{p}^{\prime }=\left(p-1\right)\theta$ in their phasor calculations. Assuming that the IRF location was *t*_0_ = 0 in the original time frame, this implies that the IRF was now located at $t_{0}^{\prime }=-\theta /4$. Computation of $L_{N\left[W\right]}$ with this parameter leads to a curve identical to that shown in the cited article. Note that this same curve is actually obtained for a large range of possible $t_{0}^{\prime }$ values, which makes the exact location of the IRF relatively irrelevant.

### B. Time-gated ICCD

In an article using a different type of detector (time-gated ICCD),^15^ Chen *et al.* reported the time-gated phasor analysis of NIR dye fluorescence with a short lifetime (*τ* ≤ 1 ns) using overlapping gates (nominally *θ* = 40 ps, *W* = 300 ps) and offset and truncated decays (*t*_0_ ∼ 1.5 ns, *T* = 12.5 ns, *D* ∼ 6 ns). Some of the calculations were also performed with non-overlapping gates (*θ* = 320 ps). The phasor harmonic used was *f* = 2/*T* = 160 MHz.

**FIG. 14.**
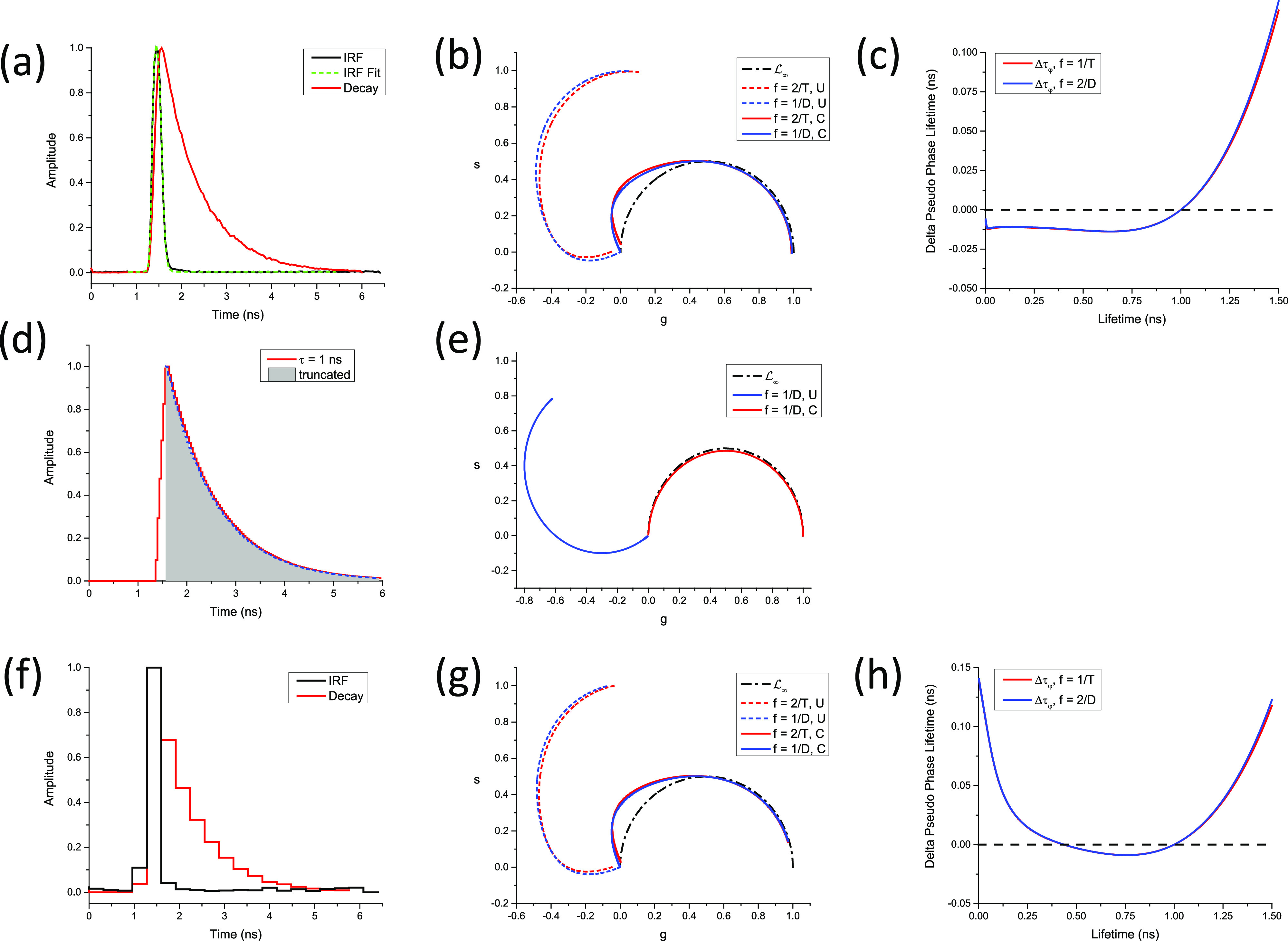
Time-gated ICCD imaging of NIR dyes (data from Ref. 15) studied using overlapping gates [(a)–(c) *N* = 150, *θ* = 40 ps] or non-overlapping gates [(f)–(h) *N* = 19. *θ* = 320 ps]. [(a) and (f)] Example of IRF and fluorescence decay. The laser period is *T* = 12.5 ns, but data were recorded only for the first 6 ns. The IRF is not square and instead is well approximated by a logistic-edge gate model with FWHM = 230 ps. [(b) and (g)] $L_{N\left[W\right]}$ computed for *W* = 230 ps and *t*_0_ = 1.57 ns are shown for two choices of phasor frequency, f = 2/*T* = 160 MHz (red) and f = 1/*D* = 167 MHz (blue) without (dashed curves) or with calibration (solid curves) using *τ* = 1 ns as reference. [(c) and (h)] Difference between pseudo-phase lifetime and true lifetime for calibrated phasors as in (b) (red: f = 2/*T*, blue: f = 1/*D*) for *τ* ≤ 1.5 ns. (d) Overlay of the 1 ns lifetime decay shown in (a) (red) and its truncated version (gray area) at *t*_0_ = 1.57 ns (duration *D* = 4.4 ns). (e) $L_{N\left[W\right]}$ for decays truncated as in (d), harmonic frequency f = 1/*D* = 227 MHz (blue: uncalibrated, red: calibrated using $L_{N}$). The phase lifetime of the calibrated phasor is obviously identical to the original lifetime since the calibrated SEPL is identical to $L_{N}$.

The first noteworthy feature of this work is that the gates used were not square due to their brevity and the finite rise and fall time of the gating electronics and microchannel plate (MCP) response: as shown in Fig. 14(a), the gated-IRF profile is well fitted by a 0-width logistic edge gate, i.e., the gate profile is dominated by the rising (*σ*_*R*_ = 21 ps) and falling (*σ*_*F*_ = 37 ps) times of the gating electronics plus MCP response (FWHM ∼ 230 ps). Because the gates are so short, this shape discrepancy is expected to have a negligible influence. The second noteworthy characteristic is that the decays are truncated (only the first ∼ 6 ns of the complete laser period are available). Finally, the IRF is offset, its peak being located at *t*_0_ ∼ 1.57 ns. Combined with the previous property, this means that only ∼ 4.5 ns of the actual decay part is available. The work used standard phasor calibration with a sample with known reference lifetime, *τ*_*C*_ = 1 ns.

Figure 14(b) shows the corresponding PSED phasor loci (dashed curves: uncalibrated $L_{N\left[W\right]}$; solid curves: calibrated or pseudo-SEPL, ${\tilde{L}}_{N\left[W\right]}$) for *f*_1_ = 2/*T* = 160 MHz (red), the phasor frequency used in Ref. 15, and for *f*_2_ = 1/*D* = 166.7 MHz, the suggested phasor frequency for a truncated decay. Due to the minor difference between these two frequencies, the results are unsurprisingly very similar. The most noteworthy feature of Fig. 14(b) is the increasing departure of ${\tilde{L}}_{N\left[W\right]}$ from $L_{\infty }$ for *τ* > *τ*_*C*_. On the other hand, the difference with $L_{\infty }$ for *τ* ≤ *τ*_*C*_ is minimal.

Figure 14(c) represents the corresponding pseudo-phase lifetime ${\tilde{\tau }}_{\varphi }$ as a function of *τ*. While unsurprisingly this difference increases for *τ* > *τ*_*C*_ (and in fact diverges for large lifetimes), the difference remains below 14 ps for *τ* ≤ *τ*_*C*_. Since that work was concerned with lifetimes shorter than 1 ns, this demonstrates that using a pseudo-phasor was appropriate.

While this analysis (already presented in an abridged form in the supplementary material of Ref. 15) justifies the use of a standard phasor calibration approach in this situation, a better solution can be found based on the present work. Namely, let us consider the decay shown in Fig. 14(a): truncating it by setting the first gate *t*_1_ = *t*_0_ and keeping all the other subsequent gates, we obtain a new recording span *D* ∼ 4.43 ns comprised of *N* = 110 gates [Fig. 14(d)]. Using *f* = 1/*D* as the phasor frequency, we find ourselves in the situation of the special case discussed in Sec. V C, for which we have shown that the locus of the phasors of PSEDs is a rotated arc of circle (in fact, it is identical to $L_{N,t_{0}}$, the SEPL for discrete, ungated PSEDs with offset *t*_0_). Using $L_{N}$ as the reference SEPL would produce calculated phase lifetimes closer to the real lifetimes (for PSEDs) than obtained in Ref. 15. However, as shown above, the discrepancy in the original work was minimal. Note also that, because the number of gates, *N* = 110, is rather large, $L_{N}$ is actually very similar to $L_{\infty }$, making it a valid reference SEPL as well for further analysis [Fig. 14(e)].

Chen *et al.* concluded by presenting results using a decimated subset of the original gates, using only 1 every 8 gates (*G* = 19, *θ* = 320 ps). The corresponding IRF and decays, illustrated in Fig. 14(f), are poorly resolved, yet the corresponding PSED phasors calculated with these new parameters [Fig. 14(g)] are quite similar to those obtained previously [compared with Fig. 14(b)]. The most notable difference is the fact that for *τ* → 0, the calibrated ${\tilde{L}}_{N\left[W\right]}$ stops short of the locus of *τ* = 0 on $L_{\infty }$, namely, the point *z* = 1. This results in a pseudo-phase lifetime ${\tilde{\tau }}_{\varphi }$ presenting a discrepancy as high as 150 ps for *τ* = 0 but below 30 ps for $\tau \in \left[0.166,\;1.217\right]$ ns [Fig. 14(h)], a range corresponding to that studied in Ref. 15.

In summary, the choice of *f* = 160 MHz and the use of standard phasor concepts and phasor calibration was justified in this work, but acquiring complete rather than truncated decays would have simplified everything.

### C. Wide-field time-gated single-photon avalanche diode array

Ulku *et al.* reported several examples of time-gated phasor analysis of visible dye fluorescence using a SPAD array imager (SwissSPAD 2)16,2^9^ characterized by long gates (*W* > 10 ns). Systematic studies of the influence of various parameters (*W*, *N*) on the calculated phase lifetime were presented in Ref. 16. The acquisition settings corresponded to discrete, non-truncated (*D* = *T* = *Nθ* = *f*^−1^), and offset (*t*_0_ = 15 ns) decays.

#### 1. Effect of gate number

**FIG. 15.**
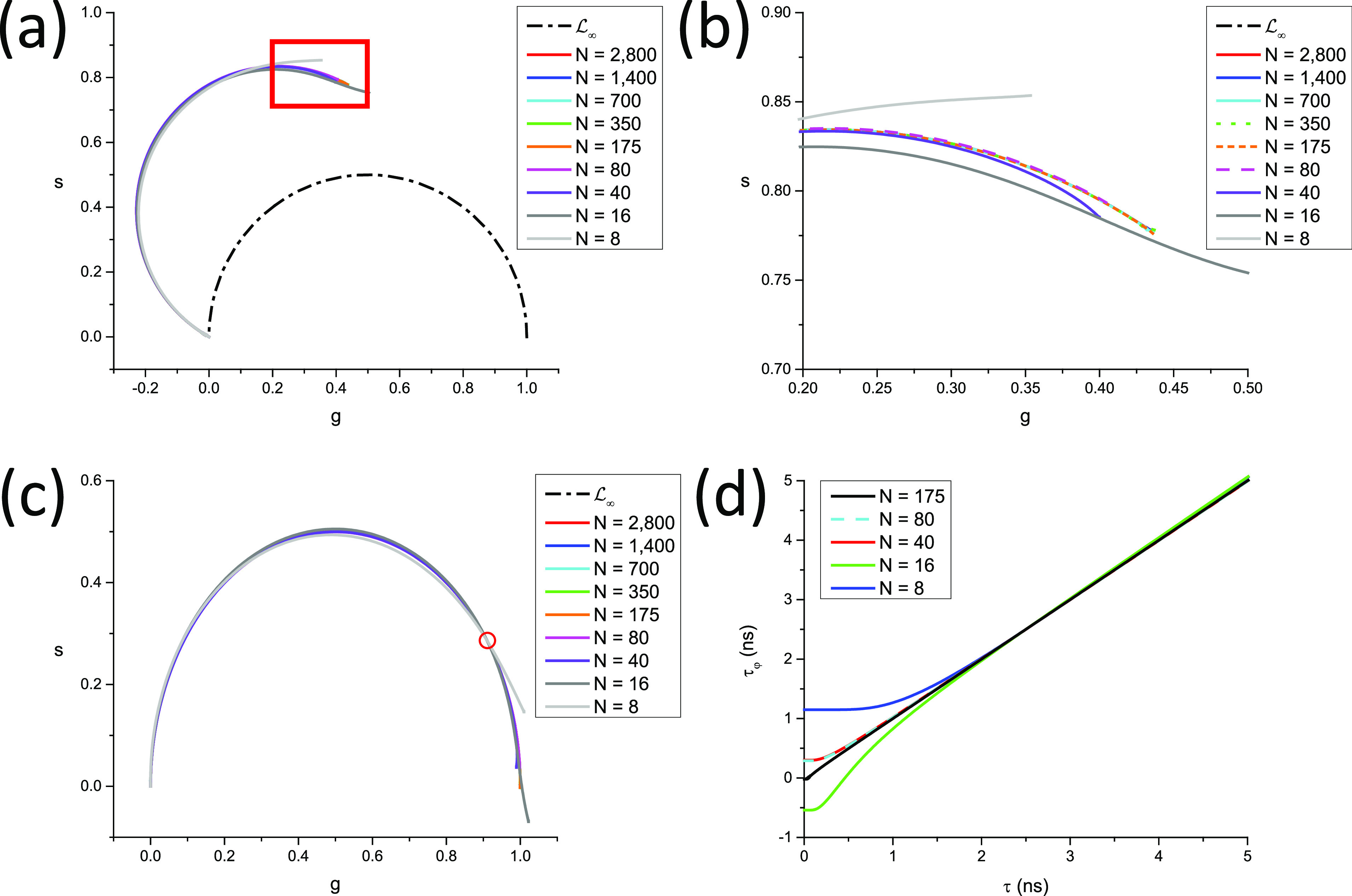
Discussion of Fig. 7 in Ref. 16. (a) Uncalibrated $L_{N\left[W\right]}$ for varying number of gates *N*. Laser period, *T* = 50 ns; phasor harmonic frequency, f = 1/*T* = 20 MHz; gate width, *W* = 13.1 ns; gate step, *θ* = *T*/*N*; IRF offset, *t*_0_ = 0. (b) Zoomed-in view of (a) in the boxed region. There are clear differences between the different curves for short lifetimes. (c) Calibrated $L_{N\left[W\right]}$ of panel (a), using *τ*_*C*_ = 2.5 ns. The curves are close to $L_{\infty }$, except for those computed with few gates (*N* < 40), which differ from them for short lifetimes. The red circle indicates the location of *τ* = *τ*_*C*_ on $L_{\infty }$. (d) Pseudophase lifetimes $\tilde{\tau }$_*φ*_ computed from the curves shown in panel (c) as a function of the known lifetime *τ*. The only major discrepancies occur for *N* < 40 and *τ* < 2 ns.

Figure 15(a) shows $L_{N\left[W\right]}$ computed with these parameters for different numbers *N* of gates (as used in Fig. 7 of Ref. 16). Due to the decay offset, the curves are rotated away from the $L_{\infty }$, and as shown in the zoomed region represented in Fig. 15(b), some discrepancies with $L_{\infty }$ are noticeable in the region of short lifetimes for *N* < 40.

After calibration using *τ*_*C*_ = 2.5 ns, as used in Ref. 16, these curves are rotated back toward $L_{\infty }$, but these discrepancies remain for short lifetimes. This can be quantified by plotting the pseudo-phase lifetime for these curves [Fig. 15(d)], which does indeed show that pseudo-phase lifetimes extracted for phasors on the calibrated $L_{N\left[W\right]}$ (*N* < 40) are significantly different from the real lifetimes. Fortunately, this effect is only significant for lifetimes *τ* < 2 ns, which are below the range of lifetimes considered in Ref. 16. Studies of shorter lifetimes would require using either a different calibration lifetime or a sufficient number of gates to avoid large discrepancies.

#### 2. Effect of gate parameter’s non-uniformity

In both Refs. 36 and 16, a local phasor calibration was used, the reason invoked being the non-uniformity of the detector’s response. This non-uniformity is detailed in Ref. 29, where the gate’s rising edge and falling edge positions, as well as the gate width, are shown to be multimodal and depend on the location of each SPAD within the array.

To analyze the effect of this non-uniformity of gate parameters on the calculated phasors, we can use the results derived here. While these works used a finite number of gates, their large number (from ∼100 in Ref. 36 to 2800 in Ref. 16) implies that the calculated phasors are close to the continuous phasor discussed in this article. Ignoring for a moment the effect of decay offset, we can use Eq. (75) for the continuous phasor of square-gated PSEDs to understand the effect of a non-uniform gate width on the calculated phasors.

The changes in the modulus prefactor *M*_*W*_ and in phase *φ*_*W*_ upon a small change *δW* in width are given by

$$\left\{\begin{array}{c} \delta M_{W}=M_{W}\left(\pi f\;W\cot\left(\pi fW\right)-1\right)\frac{\delta W}{W},\\ \delta \varphi_{W}=\varphi_{W}\frac{\delta W}{W}. \end{array}\right.$$ (194)

The effect on these variations on the calculated phase and modulus lifetimes can be computed from Eq. (156),

$$\left\{\begin{array}{c} \frac{\delta \tau_{\varphi }}{\tau_{\varphi }}=\frac{2\varphi_{W}}{\sin2\left(\varphi +\varphi_{W}\right)}\frac{\delta W}{W},\\ \frac{\delta \tau_{m}}{\tau_{m}}=\frac{{\left(M_{W}/m\right)}^{2}}{{\left(M_{W}/m\right)}^{2}-1}\left(\pi fW\cot\left(\pi fW\right)-1\right)\frac{\delta W}{W}. \end{array}\right.$$ (195)

These expressions show that phase and modulus lifetime relative changes are proportional to the relative gate width variation *δW*/*W*, with a prefactor depending on the phase *φ* or modulus *m* of the phasor under consideration. For instance, for *W* = 13.1 ns and *f* = 20 MHz (data of Fig. 7 in Ref. 16), we obtain *φ*_*W*_ = 0.823 and *M*_*W*_ = 0.891. Let us look at the influence of a *δW* = 250 ps width variation on width *W* = 13.1 ns (*δW*/*W* = 0.019) on both *τ*_*φ*_ and *τ*_*m*_ for a lifetime *τ* = 2.5 ns. Using Eq. (156), we obtain $\varphi +\varphi_{W}=\tan^{-1}\left(2\pi f\tau \right)=$ 0.304 rad and $M_{W}/m=\sqrt{1+{\left(2\pi f\tau \right)}^{2}}=$ 1.05, from which we get

$$\begin{array}{c} \frac{\delta \tau_{\varphi }}{\tau_{\varphi }}=2.88\frac{\delta W}{W}∼0.055,\\ \frac{\delta \tau_{m}}{\tau_{m}}=-2.55\frac{\delta W}{W}∼-0.049, \end{array}$$ (196)

i.e., approximately a 5% variation in phase and modulus lifetime (or ∼125 ps). This corresponds to the upper end of phase lifetime standard deviations observed in Fig. 7(b) of Ref. 16. In other words, without accounting for this systematic dispersion of phase lifetimes due to gate width variation by using local phasor calibration, smaller effects such as that of shot noise studied in Ref. 16 would have remained undetectable.

A similar analysis shows that the relative variation of phase and modulus lifetimes of a similar magnitude is induced by small gate offsets, as described in Ref. 29. Local phasor calibration solves this problem as well.

## X. CONCLUSION

In this article, we have examined the effect of several experimental parameters on the calculated phasor of periodic single-exponential decays (PSEDs), notably in cases where the traditional notion of the “universal circle” loses some of its relevance. In particular, extending the work of Ref. 14, we have provided analytical formulas for a number of practical cases encountered experimentally, not only in the analysis of data acquired with novel time-resolved approaches but also in the more traditional case of TCSPC data. Indeed, when such data are decimated or binned down to a small number of bins, one is formally in the case of discrete ungated decays and square-gated decays discussed in the text. Likewise, truncated or offset decays are encountered with all types of instrumentation and cause their own specific issues.

This study has shown that, in some cases, the resulting locus of the phasor of these PSEDs (the *single-exponential phasor locus* curve or *SEPL*, as referred to in this article) is a simple analytical curve, namely, an arc of circle, which can be mapped back to either the standard universal semicircle (noted $L_{\infty }$) or one of the discrete circular arcs, $L_{N}$, by a simple *phasor calibration* using a sample with known single lifetime. These cases are encountered when the gate width *W* is proportional to the gate step *θ*, which suggests that such a relation between width and step should be strived for whenever possible. In particular, as noted above, this situation applies to binned TCSPC data, which consist of contiguous bins (i.e., *W* = *θ*). The results presented in this work should therefore facilitate interpretation of such binned data, which have the advantage of requiring much less storage space and, accordingly, much less processing time, opening the prospect of extremely fast phasor analysis.

Even when the SEPL is not a simple curve and therefore no exact mapping to one of the simple SEPLs described in this work is possible, we have shown that calibration can be convenient to use nonetheless, provided that precautions are taken to interpret data in specific regions of the phasor plot. Such calibration, or *pseudo-calibration* in the general case, allows using conventional knowledge of the universal circle and the “canonical” phasor plot for phasor interpretation. Finally, in the general case, and when the IRF used to acquire the data can be measured, we have shown how a pseudo-calibrated SEPL can be used as a convenient reference to interpret discrete phasors.

## SUPPLEMENTARY MATERIAL

The supplementary material consists of 6 Appendixes (A–F) referred to in the text and 3 supplementary figures (S1–S3).

*Note added in proof.* Reference 39, which has recently come to my attention, takes a different approach to some of the questions discussed here.

## Data Availability

The data that support the findings of this study are available in a free online repository on Figshare at https://doi.org/10.6084/m9.figshare.11653182,^37^ and the software is available on GitHub at https://doi.org/10.5281/zenodo.3884101.^38^
